# *CDH13* promoter SNPs with pleiotropic effect on cardiometabolic parameters represent methylation QTLs

**DOI:** 10.1007/s00439-014-1521-6

**Published:** 2014-12-28

**Authors:** Margus Putku, Mart Kals, Rain Inno, Silva Kasela, Elin Org, Viktor Kožich, Lili Milani, Maris Laan

**Affiliations:** 1 Human Molecular Genetics Group, Institute of Molecular and Cell Biology, University of Tartu, Riia St. 23, 51010 Tartu, Estonia; 2 Estonian Genome Center, University of Tartu, 51010 Tartu, Estonia; 3 Institute of Mathematical Statistics, University of Tartu, 50409 Tartu, Estonia; 4 Department of Biotechnology, Institute of Molecular and Cell Biology, University of Tartu, 51010 Tartu, Estonia; 5 Department of Medicine/Division of Cardiology, David Geffen School of Medicine, UCLA, Los Angeles, CA 90095-1679 USA; 6 Institute of Inherited Metabolic Diseases, Charles University, First Faculty of Medicine, 128 08 Prague 2, Czech Republic

## Abstract

*CDH13* encodes T-cadherin, a receptor for high molecular weight (HMW) adiponectin and low-density lipoprotein, promoting proliferation and migration of endothelial cells. Genome-wide association studies have mapped multiple variants in *CDH13* associated with cardiometabolic traits (CMT) with variable effects across studies. We hypothesized that this heterogeneity might reflect interplay with DNA methylation within the region. Resequencing and EpiTYPER™ assay were applied for the HYPertension in ESTonia/Coronary Artery Disease in Czech (HYPEST/CADCZ; *n* = 358) samples to identify *CDH13* promoter SNPs acting as methylation Quantitative Trait Loci (meQTLs) and to investigate their associations with CMT. In silico data were extracted from genome-wide DNA methylation and genotype datasets of the population-based sample Estonian Genome Center of the University of Tartu (EGCUT; *n* = 165). HYPEST–CADCZ meta-analysis identified a rare variant rs113460564 as highly significant meQTL for a 134-bp distant CpG site (*P* = 5.90 × 10^−6^; β = 3.19 %). Four common SNPs (rs12443878, rs12444338, rs62040565, rs8060301) exhibited effect on methylation level of up to 3 neighboring CpG sites in both datasets. The strongest association was detected in EGCUT between rs8060301 and cg09415485 (false discovery rate corrected *P* value = 1.89 × 10^−30^). Simultaneously, rs8060301 showed association with diastolic blood pressure, serum high-density lipoprotein and HMW adiponectin (*P* < 0.005). Novel strong associations were identified between rare *CDH13* promoter meQTLs (minor allele frequency <5 %) and HMW adiponectin: rs2239857 (*P* = 5.50 × 10^−5^, β = −1,841.9 ng/mL) and rs77068073 (*P* = 2.67 × 10^−4^, β = −2,484.4 ng/mL). Our study shows conclusively that *CDH13* promoter harbors meQTLs associated with CMTs. It paves the way to deeper understanding of the interplay between DNA variation and methylation in susceptibility to common diseases.

**Electronic supplementary material:**

The online version of this article (doi:10.1007/s00439-014-1521-6) contains supplementary material, which is available to authorized users.

## Introduction

The relevance of the *Cadherin*-*13* gene (*CDH13*; 1.2 Mb) in a wide spectrum of biomedical fields—oncology, neurology, cardiovascular physiology—was recognized over a decade ago (Takeuchi and Ohtsuki 2001). *CDH13* encodes T-cadherin, which belongs to the cadherin gene family of cell adhesion molecules (Ranscht and Dours-Zimmermann 1991). Its expression was first described in the developing chicken embryo and shown to be widely distributed throughout the entire avian and mammalian nervous systems (Rivero et al. 2013). Subsequent studies in vascular tissue identified high expression of T-cadherin in endothelial and smooth muscle cells, as well as specifically in cardiac myocytes (Philippova et al. 2009). Localized in membrane lipid rafts, T-cadherin functions in promoting survival, proliferation and migration of endothelial cells and in protecting cells from oxidative stress-induced apoptosis (Philippova et al. 2009; Joshi et al. 2005). In cardiovascular metabolism, it exhibits ligand-binding ability uncommon to classical cadherins, acting as the third receptor for high molecular weight (HMW) adiponectin and also binding low-density lipoprotein (LDL) (Tkachuk et al. 1998; Hug et al. 2004). Low circulating adiponectin levels (hypoadiponectinemia: <4 μg/mL) are associated with not only various cardiovascular and metabolic phenotypes [e.g., type 2 diabetes (T2D), hypertension, dyslipidemia, atherosclerosis, coronary artery disease and stroke], but also with gastrointestinal diseases, osteoporosis and cancers (Kishida et al. 2014). A considerable number of human cancer genomes are characterized by hypermethylated *CDH13* promoter, and down-regulation of its transcription promotes tumor growth and invasiveness (Andreeva and Kutuzov 2010).

**Fig. 1:**
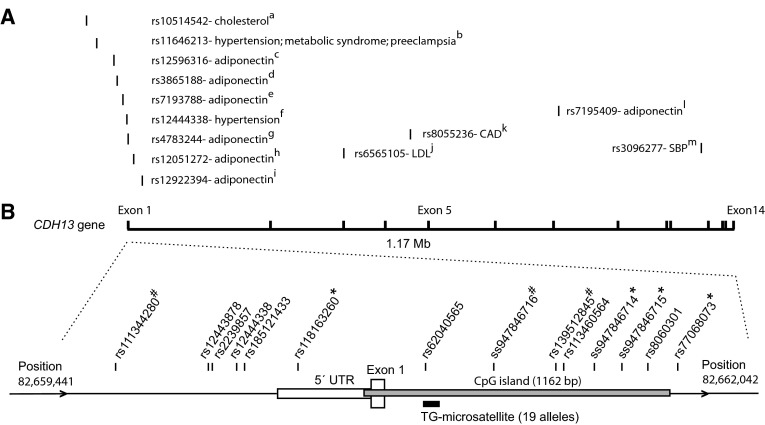
Resequencing of the *CDH13* promoter region in the HYPEST and CADCZ sample sets. **a** Illustrative map of previously identified genetic associations between cardiometabolic traits and SNPs in the *CDH13* genomic region (1.17 Mb; GRCh37/hg19 Chr.16: 82,660,399–83,830,215; exons 1–14) is shown at the relative genomic scale. *Numbers in superscript* indicate the respective publications in the Reference List. **b** The *CDH13* promoter region targeted for resequencing (2,602 bp; Chr.16: 82,659,441–82,662,042; bordered by *arrows* ‘>’) is zoomed, comprising the 5′ UTR (*horizontal box*), exon 1 (*vertical box*) and CpG island (*grey box*) of the gene. The location of SNPs and the polymorphic (TG)_n_ microsatellite detected in the current study is shown; population-specific SNPs are marked with ‘*’ (HYPEST) or ‘#’ (CADCZ). *CAD* coronary artery disease, *LDL* low-density lipoprotein, *SBP* systolic blood pressure. ^a^Wallace et al. (2008); ^b^Org et al. (2009), Fava et al. (2011), Wan et al. (2013); ^c^Jee et al. (2010); ^d^Jee et al. (2010), Wu et al. (2010), Jo et al. (2012); ^e^Jee et al. (2010), Chung et al. (2011); ^f^Lee et al. (2013); ^g^Chung et al. (2011), Morisaki et al. (2012), Gao et al. (2013), Uetani et al. (2014); ^h^Morisaki et al. (2012), Dastani et al. (2012); ^i^Dastani et al. (2012); ^j^Lee et al. (2013); ^k^Wellcome Trust Case Control Consortium (2007); ^l^Ling et al. (2009); ^m^Levy et al. (2007)

The era of genome-wide association studies (GWAS) has brought further evidence of pleiotropic effects attributed to *CDH13*. Genetic risk variants in *CDH13* have been identified for cancer (Thomas et al. 2008) and neuropsychiatric disorders such as attention-deficit/hyperactivity disorder (ADHD), autism and dependence on psychotic substances (Rivero et al. 2013; Redies et al. 2012). However, the most notable genetic association signals in the *CDH13* gene have been detected for a spectrum of cardiovascular and metabolic traits (Fig. 1). The strongest and the largest number of associations, mainly for a cluster of SNPs in the promoter region, have been reported for serum adiponectin levels and many of these findings have been replicated in diverse ethnic populations (Ling et al. 2009; Jee et al. 2010; Wu et al. 2010; Chung et al. 2011; Morisaki et al. 2012; Dastani et al. 2012; Gao et al. 2013). Decreased serum adiponectin levels have recently been showed for ADHD patients suggesting its possible involvement in the pathophysiology of ADHD (Mavroconstanti et al. 2014). SNPs in *CDH13* have been associated with total cholesterol and LDL levels (Dong et al. 2011; Lee et al. 2013), coronary artery disease (CAD) (Wellcome Trust Case Control Consortium 2007), hypertension and blood pressure (Org et al. 2009; Levy et al. 2007; Lee et al. 2013), hyperlipidemia and myocardial infarction (Shia et al. 2011), metabolic syndrome (Fava et al. 2011) and preeclampsia (Wan et al. 2013).

Despite convincing evidence of the contribution of genetic variation in *CDH13* to cardiometabolic traits, the primary causative SNP has not been identified and a multitude of contributing common variants with variable effects across studies has been reported (Fig. 1). DNA methylation has been suggested as a potential mediator of genetic risk for common diseases (Liu et al. 2013; Koestler et al. 2014). Low DNA methylation level in genomic regions associated with T2D in GWA studies was reported as an early marker of T2D suggesting early-onset, inter-individual methylation variation at isolated genomic sites that modify the predisposition to T2D (Toperoff et al. 2012). For the *CDH13* gene, seminal reports have shown significant inter-individual variation in DNA methylation and described SNPs in the *CDH13* gene that affect the methylation of nearby CpG sites (Flanagan et al. 2006, 2009; Zhi et al. 2013).

In the current study, we hypothesized that the heterogeneity of the identified genetic associations in *CDH13* with a number of cardiometabolic traits might reflect interplay of inter-individual differences in DNA methylation variation. The study aimed (i) to identify SNPs in the *CDH13* promoter region modulating DNA methylation of nearby CpG sites referred as methylation Quantitative Trait Loci (meQTLs); (ii) to investigate the genetic association of the identified meQTLs with serum adiponectin, lipids and blood pressure and (iii) to address the effect of DNA methylation level per se on the studied quantitative cardiometabolic parameters (Figure S1). We relied on the investigation of DNA extracted from whole blood, as the basic inter-individual variations in DNA methylation levels stemming from the difference in the genetic composition among study subjects are expected to be detectable across the majority of the cell types.

## Materials and methods

### HYPEST and CADCZ subjects for *CDH13* DNA methylation analysis and resequencing

Participants of HYPertension in ESTonia (HYPEST; full sample: *n* = 1,966) have been recruited across Estonia during 2004–2007 with the aim to analyze genetic-epidemiological risk factors for cardiovascular disease (CVD) (Org et al. 2011). Subjects of Coronary Artery Disease in Czech study (CADCZ; full sample: *n* = 869) have been recruited across Czech Republic during 1998–2000 with the aim to study genetic factors related to homocysteine metabolism in coronary artery disease (CAD) (Janosíková et al. 2003). The recruitment has been carried out in compliance with the Helsinki Declaration and all participants have given written informed consent. The HYPEST study has been approved by the Ethics Committee on Human Research of the University of Tartu (permissions 122/13, 22.12.2003; 137/20, 25.04.2005). The CADCZ study has been approved by the Ethics Committee of Charles University—First Faculty of Medicine (December 1996).

The current (epi)genetic study used a subset of middle-aged patients (total, *n* = 358) from HYPEST (*n* = 192; aged 34–62, mean 50.5 ± 5.2 years) and CADCZ (*n* = 166; aged 33–61, 50.1 ± 4.6 years) sample sets (Table 1). Study inclusion criteria, measurement of blood pressure and serum lipids and correction of the measured values prior to the genetic data analysis for the subjects under medication are described in the Supplemental Methods. Serum samples of HYPEST subjects were stored at −86 °C immediately after blood draw. The concentration of high molecular weight (HMW) adiponectin in serum was measured for the study subjects with available stored serum samples (*n* = 184) using the Human HMW Adiponectin/Acrp30 ELISA assay (R&D Systems) according to the manufacturer’s protocol (Supplemental Methods).

**Table 1:** Characteristics of study samples for (epi)genetic analysis

Parameter (mean ± SD)	CVD study participants		Population cohort
	HYPEST	CADCZ	EGCUT
No of individuals	192	166	165
Men/women	93/99	114/52^#^	72/93*
Age (years)	50.5 ± 5.2	50.1 ± 4.6	41.6 ± 22.7*
BMI (kg/m^2^)	30.2 ± 5.4	27.3 ± 4.1^#^	25.0 ± 4.8*
SBP (mmHg)	140.1 ± 18.7	127.1 ± 16.2^#^	123.1 ± 17.4*
DBP (mmHg)	88.6 ± 11.7	81.5 ± 9.5^#^	76.5 ± 10.6*
Total cholesterol (mmol/L)	5.6 ± 1.2	5.3 ± 0.9^#^	5.3 ± 1.1
LDL (mmol/L)	3.7 ± 1.0	3.1 ± 0.8^#^	3.2 ± 0.9*
HDL (mmol/L)	1.5 ± 0.4	1.3 ± 0.4^#^	1.6 ± 0.4*
Triglycerides (mmol/L)	1.7 ± 1.2	1.9 ± 1.2	1.4 ± 0.9*
HMW adiponectin (ng/mL)	4,564.3 ± 3,602.9^a^	NA	NA
Clinically diagnosed hypertension (%)	86.4	25.5^#^	11.5*
Coronary artery disease (%)	22.2	50^#^	6.1*
Early (<58 years) myocardial infarction (%)	14.6	44.6^#^	0.6*
Antihypertensive treatment (%)	86.4	25.5 ^#^	24.2*
Antilipidemic treatment (%)	15.6	32.5^#^	5.5*

*HMW* high molecular weight, *CADCZ* Coronary Artery Disease in Czech, *CVD* cardiovascular disease, *EGCUT* University of Tartu Estonian Genome Center, *HYPEST* HYPertension in ESTonia

^#^HYPEST versus CADCZ, * HYPEST + CADCZ versus EGCUT, *P* < 0.05; the Chi-square test for categorical and the Student’s *t* test or Mann–Whitney test for continuous variables that had a normal or skewed distribution, respectively

^a^Men 3,268.3 ± 2,811.5 ng/mL; women 5,701.6 ± 3,842.8 ng/mL

### EGCUT sample subjected to genome-wide DNA methylation profiling and genotyping

The population-based biobank of the Estonian Genome Center of the University of Tartu (EGCUT) has been recruited across Estonia in 2003–2010 (http://www.geenivaramu.ee/en/). EGCUT includes epidemiological–clinical datasets and DNA samples extracted from blood for Estonian adults across all age groups (*n* = 51,515; Leitsalu et al. 2014). Measurement of blood pressure and serum lipids in the EGCUT samples are provided in the Supplemental Methods. In this study, in silico data for the *CDH13* region were extracted and analyzed for the EGCUT samples with available datasets for both genome-wide DNA methylation and genome-wide imputed genotypes (*n* = 165; aged 18–84; 41.6 ± 22.7 years).

### EpiTYPER™ analysis of DNA methylation in the *CDH13* promoter in HYPEST and CADCZ samples

The suitability of DNA extracted from whole blood for the reliable CpG methylation profiling at the *CDH13* locus was assessed using a published genome-wide DNA methylation dataset of purified human blood cells (Reinius et al. 2012) (Supplemental Methods). All analyzed blood cell types demonstrated similar CpG methylation profile across the *CDH13* region (Figure S2).

The MassARRAY EpiTYPER™ assay (Sequenom, San Diego, CA, USA) was applied to measure DNA methylation in the *CDH13* promoter for the HYPEST/CADCZ subjects. The targeted CpG sites were located within the CpG island (1,162 bp; GRCh37/hg19, Chr16: 82,660,652–82,661,813) and in the flanking 361 bp 5′ upstream region (Figure S3). Four EpiTYPER™ assays were designed to cover 110 CpG sites (13–40 CpG sites/assay) within a ~1.5 kb target region using the EpiDesigner software as instructed by the manufacturer (Table S1; Figure S3). Measurement of DNA methylation at targeted CpG sites followed the established experimental and analytical protocols (Ehrich et al. 2005) (http://bioscience.sequenom.com/sites/bioscience.sequenom.com/files/EpiTYPER%20Application%20Note.pdf) and was implemented using MassARRAY Analyzer 4 (Sequenom Inc.). The analyzed sequence fragments (1–57 bp) containing >1 CpG sites were named as CpG units and the methylation value for a CpG unit was calculated as average methylation across the CpG sites forming a unit. Singleton CpG sites per fragment were assessed individually. Experimental details of the EpiTYPER™ assay and quality control (QC) steps of CpG methylation measurements performed prior to the statistical association testing are provided in the Supplemental Methods. After stringent QC, DNA methylation at 66 CpG sites in the *CDH13* promoter (33 CpG sites clustered in 13 CpG units and 33 individual CpG sites; Table S2) was subjected to the association testing with nearby SNPs and phenotypic traits.

### Resequencing of *CDH13* promoter region in HYPEST and CADCZ samples

The promoter region of *CDH13* was resequenced in HYPEST (*n* = 192) and CADCZ (*n* = 166) samples. The resequenced region spanned 2,602 bp (GRCh37/hg19, Chr16: 82,659,441–82,662,042) and included the entire CpG island of the *CDH13* gene (1,162 bp, Chr:16: 82,660,652–82,661,813) (Fig. 1, Figure S3). The region was amplified by long-range PCR (Supplemental Methods) and resequenced as described by Hallast et al. (2005) on both forward and reverse strands using 10 sequencing primers (Table S1). Sequences were assembled and SNPs were identified using CodonCode Aligner (http://www.codoncode.com/aligner/). Estimation of allele frequencies and conformance to Hardy–Weinberg Equilibrium (HWE; χ^2^, *P* > 0.05) were implemented in the PLINK v1.07 software (Purcell et al. 2007).

### Extraction of CpG methylation data for the *CDH13* promoter region from EGCUT genome-wide DNA methylation dataset

Genome-wide DNA methylation profiling (Infinium HumanMethylation450 BeadChips) of EGCUT samples (*n* = 165) was performed according to the manufacturer’s recommendations (Supplemental Methods). The original IDAT files were extracted from the HiScanSQ scanner. Data pre-processing and QC analyses were performed in R using the Bioconductor package *minfi* version 3.0.1 (Aryee et al. 2014) (Supplemental Methods). After QC steps, DNA methylation levels at the 57 CpG sites across the *CDH13* genic region (GRCh37/hg19, Chr16: 82,474,489–83,829,911) were tested in association with allelic profile of the SNPs at the *CDH13* promoter. Eight promoter CpG sites analyzed in EGCUT overlapped with the HYPEST–CADCZ dataset (EpiTYPER™ assays; Table S3).

### Extraction of *CDH13* genotyping data from EGCUT genome-wide dataset

Genomic DNA of EGCUT subjects (*n* = 165) was genotyped using HumanOmniExpress BeadChips (Illumina) according to the manufacturer’s instructions. The following QC filters were applied: sample call rate > 0.95, SNP call rate > 0.95, MAF > 0.01 and HWE *P* value > 0.00001. Genotype imputation is detailed in the Supplemental Methods.

In the current study, four SNPs in the *CDH13* promoter region, which overlapped between HYPEST/CADCZ and EGCUT datasets were targeted in the genetic association testing of CpG methylation levels and cardiometabolic parameters. One of these SNPs had been genotyped (rs12443878) and the other three imputed (rs12444338, rs62040565, rs8060301). EGCUT genotype data was also exploited to calculate linkage disequilibrium (LD; *r* ^2^) between these SNPs and previously reported SNPs associated with cardiometabolic traits (Table S4).

### Genetic association testing with CpG methylation levels: meQTL analysis

Seven *CDH13* promoter SNPs shared by HYPEST and CADCZ (Fig. 1c) were tested for association with DNA methylation levels at the 46 studied *ci*s-CpG sites/units (Figure S3). Prior to association analysis, the effect of age as a potential confounder of CpG methylation was assessed and no correlations remained significant after multiple testing correction (Table S5). Tests were performed using linear regression with an additive model (age, gender and experiment series as covariates) implemented in PLINK v1.07 (Purcell et al. 2007) and the combined meta-analysis was carried out using the inverse-variance method under a fixed-effects model implemented in R, ver. 3.0.2 (R Development Core Team 2014, http://www.r-project.org/). Meta-analysis of HYPEST and CADCZ results was used instead of joint analysis of the study subjects to eliminate confounding factors due to potential population stratification. Only those associations (nominal *P* value < 0.05) supported by enhanced statistical significance in the meta-analysis compared to separate tests for both HYPEST and CADCZ datasets were considered as potentially true results. Bonferroni threshold was calculated: *α* = 0.05/[2 (studies) × 7 (SNPs) × 46 (CpG sites/units)] = 7.76 × 10^−5^.

For meQTL confirmation analysis in the EGCUT sample set, the SNPs in the *CDH13* promoter region overlapping with the HYPEST/CADCZ dataset (rs12443878, rs12444338, rs62040565, rs8060301) were assessed for the effect on the DNA methylation level at the CpG sites measured across the *CDH13* gene (*n* = 57) in EGCUT. Linear regression coefficients were calculated to detect association between SNPs and the variation in the methylation levels. To correct for multiple testing, Benjamin–Hochberg false discovery rate (FDR) was estimated at 5 %, implemented using the p.adjust package in R.

### Genetic association testing with cardiometabolic parameters

The effect of genotypes on cardiometabolic traits [total cholesterol, low- and high-density lipoproteins (LDL, HDL), triglycerides, systolic and diastolic blood pressure (SBP, DBP)] was tested in HYPEST, CADCZ and ECGUT meta-analysis using linear regression implemented in PLINK v1.07 (Purcell et al. 2007), and models were adjusted for age and gender. Multiple testing threshold was estimated: *α* = 0.05/[3 (studies) × 4 (SNPs) × 5 (SBP, DBP, HDL, LDL and triglycerides] = 8.33 × 10^−4^.

Genetic association testing with serum HMW adiponectin levels was applicable for 11 SNP detected in resequencing the HYPEST samples. In addition to gender and age, BMI [obesity affects adiponectin level (Arita et al. 1999)] and plate batch in the ELISA assay (to minimize the inter-assay effect) were incorporated into the model as covariates. Tests were implemented with natural logarithm-transformed adiponectin values.

### Analysis of association between DNA methylation levels and cardiometabolic traits

The effect of methylation levels at the 46 *CDH13* CpG sites/units on serum lipids and BP in HYPEST and CADCZ subjects was tested using linear regression in R incorporating age, gender and experiment series in the model. The results were combined in a meta-analysis as described above and the Bonferroni threshold was estimated: *α* = 0.05/[2 × (studies) × 5 (parameters) × 46 (CpG sites/units)] = 1.09 × 10^−4^.

## Results

### *CDH13* promoter meQTLs

The methylation profiling of the *CDH13* promoter in the HYPEST/CADCZ (HYPertension in ESTonia/Coronary Artery Disease in Czech) study sample (*n* = 358) resulted in the methylation levels of 46 CpG sites/units within 1,162 bp of its CpG island and flanking 361 bp 5′ upstream region (Fig. 1, Figure S3). Resequencing of the *CDH13* promoter (2,602 bp) resulted in 14 SNPs, seven shared among the HYPEST and CADCZ samples (Fig. 1; Table S6). The rest of the SNPs were population-specific, including one and two novel variants for CADCZ and HYPEST, respectively. In addition, a highly polymorphic TG microsatellite (19 length variants) within the CpG island was detected with similar allelic distribution in the two sample sets (Supplemental Methods, Figure S4). One SNP, rs62040565 was located inside the TG microsatellite (Fig. 1).

**Fig. 2:**
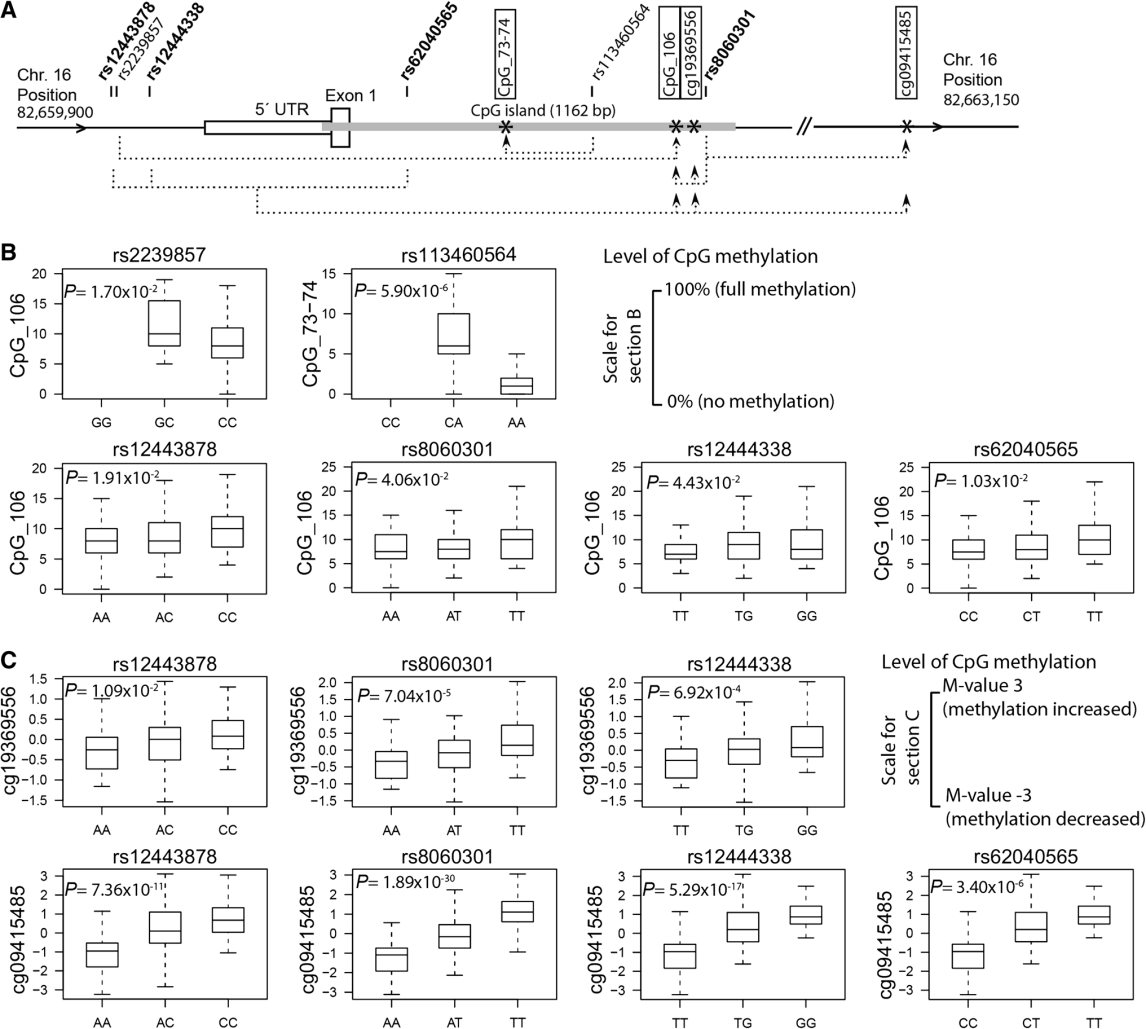
Identified *CDH13* promoter meQTLs modulating methylation levels in neighboring CpG sites. **a** *Dotted lines* connect meQTLs and CpG sites (*boxed*) showing significantly modulated methylation (*) in HYPEST–CADCZ (CpG_106, CpG_73–74) and EGCUT (cg19369556, cg09415485). Common SNPs are marked in *bold*. **b** Genotype effects of identified mQTLs on DNA methylation at selected CpG sites in HYPEST–CADCZ meta-analysis. *Y*-axis depicts measured DNA methylation level (%; 0 % no methylation, 100 % full methylation). *P* value was calculated using linear regression, including age, gender and experiment series as covariates in the model. **c** Genotype effects of tested mQTLs on DNA methylation variation in the EGCUT dataset. *Y*-axis represents relative DNA methylation level on transformed scale (*M* value). FDR-corrected *P* values were calculated using linear regression

Association testing was performed between the seven shared SNPs and DNA methylation levels at the 46 CpG sites/units measured in HYPEST/CADCZ. A rare variant rs113460564 (MAF: HYPEST 0.8 %, CADCZ 2.1 %) within the CpG island was identified as a statistically significant meQTL located 134 bp from the major modulated CpG site (HYPEST: *P* = 4.25 × 10^−4^; CADCZ: *P* = 2.64 × 10^−3^; meta-analysis: *P* = 5.90 × 10^−6^, resistant to multiple testing correction; Table 2; Fig. 2a, b). Carrying one copy of the rs113460564 minor allele accounted for 2.8 or 5.5 % increased methylation at the CpG unit CpG_73–74 in HYPEST and CADCZ, respectively [meta-analysis: β (SE) = 3.19 (0.70)]. Five additional SNPs showed a non-significant trend for acting as meQTLs, potentially affecting DNA methylation levels of up to 3 CpG sites located 74–1,737 bp from the respective SNPs (Table 3). The length of the TG microsatellite did not show any evidence of association with DNA methylation levels at the tested CpG sites (data not presented).

**Table 2:** Results of HYPEST–CADCZ meta-analysis for genetic association tests between SNPs and DNA methylation at the CpG sites within the *CDH13* promoter region

SNP/tested allele	CpG site/unit: its position at chr. 16^a^	Distance between SNP and CpG (bp)^b^	HYPEST		CADCZ		Meta-analysis^d^	
			β (SE)^c^ 95 % CI	*P* value	β (SE) 95 % CI	*P* value	β (SE) 95 % CI	*P* value
rs113460564 C	CpG_73–74 82,661,285	134	2.77 (0.77) [1.27, 4.27]	4.25 × 10^−4^	5.46 (1.78) [1.96, 8.95]	2.64 × 10^−3^	3.19 (0.70) [1.81, 4.56]	5.90 × 10^−6^
rs2239857 G	CpG_13 82,660,554	611	−1.01 (0.38) [−1.76, −0.26]	9.56 × 10^−3^	−0.97 (1.58) [−4.07, 2.14]	5.42 × 10^−1^	−1.01 (0.37) [−1.74, −0.27]	7.04 × 10^−3^
	CpG_106 82,661,670	1,727	3.12 (2.04) [−0.88, 7.12]	1.30 × 10^−1^	5.57 (2.84) [0.01, 11.13]	5.17 × 10^−2^	3.96 (1.66) [0.71, 7.20]	1.70 × 10^−2^
	CpG_3 82,660,376	433	−3.28 (1.61) [−6.43, −0.12]	4.37 × 10^−2^	−3.68 (4.78) [−13.05, 5.69]	4.43 × 10^−1^	−3.32 (1.53) [−6.31, −0.33]	2.97 × 10^−2^
rs12444338 T	CpG_116 82,661,812	1,657	3.11 (1.50) [−6.04, −0.17]	4.10 × 10^−2^	1.35 (0.70) [−0.02, 2.73]	5.60 × 10^−2^	1.67 (0.64) [0.42, 2.91]	8.61 × 10^−3^
	CpG_106 82,661,670	1,515	−0.92 (0.75) [−0.55, 2.39]	2.23 × 10^−1^	−0.74 (0.46) [−1.64, 0.16]	1.11 × 10^−1^	−0.79 (0.39) [−1.56, −0.02]	4.43 × 10^−2^
rs62040565^e^ C	CpG_106 82,661,670	724	−1.18 (0.70) [−0.19, 2.54]	9.59 × 10^−2^	−0.94 (0.48) [−1.88, 0.01]	5.28 × 10^−2^	−1.02 (0.40) [−1.79, −0.24]	1.03 × 10^−2^
	CpG_3 82,660,376	570	0.82 (0.64) [−2.07, 0.44]	2.04 × 10^−1^	1.50 (0.71) [0.10, 2.90]	3.70 × 10^−2^	1.12 (0.48) [0.19, 2.05]	1.85 × 10^−2^
rs12443878 A	CpG_106 82,661,670	1,737	−1.10 (0.69) [−0.25, 2.45]	1.14 × 10^−1^	−0.83 (0.48) [−1.76, 0.10]	8.35 × 10^−2^	−0.92 (0.39) [−1.68, −0.15]	1.91 × 10^−2^
rs8060301 A	CpG_106 82,661,670	74	−1.07 (0.73) [−0.35, 2.50]	1.43 × 10^−1^	−0.73 (0.49) [−1.69, 0.24]	1.44 × 10^−1^	−0.84 (0.41) [−1.64, −0.04]	4.06 × 10^−2^

*SE* standard error, *CI* confidence interval

^a^Numbering and the precise localization of CpG sites/units within the *CDH13* promoter region are provided in Figure S3. Promoter CpG sites 1–15 are located upstream of the CpG island, sites 16–116 are within the CpG island and sites 18–26 are in the first exon. Genomic position on chromosome 16 is according to GRCh37/hg19. In case of CpG units, the position of the first CpG site in the unit is given

^b^In case of CpG units, distance between SNP and the closest CpG site in the unit is given

^c^Tested allele effect on DNA methylation measured on a scale from 0 to 100 units; *P* values are calculated using linear regression, including age, gender and experiment series as covariates in the model

^d^Only associations, which demonstrated enhanced statistical significance in meta-analysis compared to both sample-specific tests, are shown; results were combined using the inverse-variance method under a fixed-effects model; underlined *P*-value denotes statistically significant association after Bonferroni correction (*α* = 7.76 × 10^−5^)

^e^Located within a polymorphic TG microsatellite and polymorphic substitution T to C creates an additional CpG site

**Table 3:** Genetic association between four overlapping common SNPs identified in the HYPEST and CADCZ study groups and DNA methylation in the EGCUT sample set

SNP	Tested allele	CpG site^a^	CpG position at chr. 16^b^	Distance between SNP and CpG (bp)	β (SE)^c^	95 % CI	*P* value	FDR^c^
rs8060301	A	cg09415485	82,663,111	1,367	−0.68 (0.06)	[−0.79, −0.57]	1.53 × 10^−32^	1.89 × 10^−30^
		cg19369556	82,661,725	19	−0.35 (0.07)	[−0.49, −0.20]	2.84 × 10^−6^	7.04 × 10^−5^
rs12444338	T	cg09415485	82,663,111	2,956	−0.57 (0.06)	[−0.70, −0.45]	8.53 × 10^−19^	5.29 × 10^−17^
		cg19369556	82,661,725	1,570	−0.31 (0.07)	[−0.46, −0.16]	3.35 × 10^−5^	6.92 × 10^−4^
rs12443878	A	cg09415485	82,663,111	3,178	−0.49 (0.07)	[−0.62, −0.35]	1.78 × 10^−12^	7.36 × 10^−11^
		cg19369556	82,661,725	1,792	−0.26 (0.08)	[−0.41, −0.11]	6.15 × 10^−4^	1.09 × 10^−2^
		cg09044981	82,827,677	167,744	0.26 (0.08)	[0.11, 0.41]	7.53 × 10^−4^	1.17 × 10^−2^
rs62040565	C	cg09415485	82,663,111	2,165	−0.39 (0.07)	[−0.53, −0.24]	1.10 × 10^−7^	3.40 × 10^−6^

*SE* standard error, *CI* confidence interval

^a^CpG site ID on Infinium HumanMethylation450 BeadChip

^b^Genomic position on chromosome 16 (GRCh37/hg19)

^c^False discovery rate (FDR) corrected *P* value; only associations, significant after FDR correction, are given. Effects (tested allele effect on DNA methylation on transformed scale, see “Materials and methods”) and *P* values are calculated using linear regression

Four SNPs (rs12443878, rs12444338, rs62040565 and rs8060301) showing evidence to act as meQTLs in HYPEST and CADCZ analysis overlapped with SNPs genotyped or imputed in the EGCUT (Estonian Genome Center of the University of Tartu) sample set. These SNPs were tested for association with methylation levels at 57 CpG sites measured across the *CDH13* genic region in the EGCUT sample (Table S7). All four SNPs were confirmed as meQTLs and showed significant effects (FDR < 0.05) on the methylation levels of up to 3 CpG sites located 19–167,744 bp from the respective SNPs. The strongest association was detected between rs8060301 located within the CpG island and a CpG site cg09415485 at a distance of 1.3 kb (FDR 1.89 × 10^−30^; Table 3; Fig. 2a, c).

### Associations between *CDH13* promoter meQTLs and cardiometabolic parameters

**Fig. 3:**
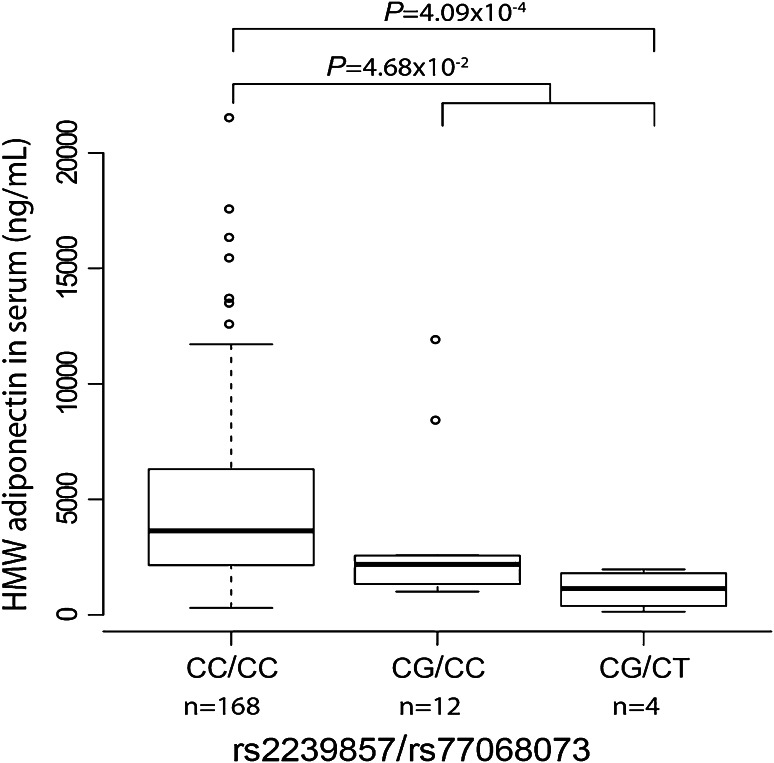
Novel genetic variants rs2239857 and rs77068073 associated with adiponectin level in blood serum. HMW adiponectin levels are shown for analyzed HYPEST subjects stratified based on their genotypes: heterozygotes for rs2239857 minor allele (*n* = 12, CG/CC), ‘double’ heterozygotes for both, rs2239857 and rs77068073 (*n* = 4, CG/CT) and homozygotes for major alleles of both SNPs (*n* = 168, CC/CC). The *boxes* represent the 25th and 75th percentiles. The median is denoted as the *line* that bisects the *boxes*. The *whiskers* are lines extending from each end of the box covering the extent of the data on 1.5× interquartile range. *Circles* represent the outlier values. *P* values from the *t* test comparing adiponectin levels of CC/CC group with CG/CT group and combined group of CG/CC and CG/CT are shown

Next, genetic associations of identified meQTLs with cardiometabolic parameters were investigated. As the strongest associations of the *CDH13* promoter region have been published for serum adiponectin, we firstly tested association with HMW adiponectin levels measured in HYPEST blood samples (unavailable for CADCZ, EGCUT). The strongest association was detected for two rare variants flanking the CpG island [rs2239857, C/G: MAF = 4.2 %, *P* = 5.50 × 10^−5^, β (SE) = −1,841.9 (711.9) ng/mL; rs77068073, C/T: MAF = 1 %, *P* = 2.67 × 10^−4^, β (SE) = −2,484.4 (1,463.1) ng/mL; Table 4; Fig. 1]. The lowest adiponectin levels (mean 1,095.1 ng/mL) were detected among the individuals who were heterozygous for both rs77068073 T-allele and rs2239857 G-allele (*n* = 4; Fig. 3). This represents approximately 4.5-fold lower adiponectin concentration compared to the study subjects carrying neither of these variants (mean 4,680.2 ng/mL). Subjects heterozygous for only rs2239857 (*n* = 12) also had reduced adiponectin levels (mean 3,356.8 ng/mL). In the SNP–CpG methylation association testing, rs2239857 had exhibited potential as a meQTL for 3 CpG sites (Table 2).

**Table 4:** Association testing of *CDH13* promoter SNPs with HMW adiponectin level in blood serum (ng/mL) in HYPEST

SNP	Major/minor allele^a^	Tested allele	Tested allele frequency	β (SE) [ng/mL]	95 % CI	*P* value^b^
rs2239857	C/G	G	0.042	−1,841.9 (711.7)	[−2,318.3, −1,147.9]	5.50 × 10^−5^
rs77068073	C/T	T	0.010	−2,484.4 (1,463.1)	[−2,928.4, −1,582.3]	2.67 × 10^−4^
rs8060301	T/A	A	0.542	−955.2 (273.2)	[−1,669.9, −342.2]	1.63 × 10^−3^
rs12444338	G/T	T	0.599	−682.1 (278.3)	[−1,364.3, −98.4]	2.13 × 10^−2^
rs113460564	A/C	C	0.008	6,492.0 (3,684.4)	[−1,051.7, 38,677.6]	1.48 × 10^−1^
rs62040565	T/C	C	0.536	−321.2 (264.9)	[−911.5, 187.6]	2.31 × 10^−1^
rs12443878	C/A	A	0.549	−231.7 (267.2)	[−812.7, 268.5]	3.85 × 10^−1^
rs185121433	G/T	T	0.018	558.8 (1,107.1)	[−1,117.2, 3,488.5]	5.90 × 10^−1^
ss947846715	C/G	G	0.005	−649.2 (2,362.3)	[−2,405.0, 4,336.8]	6.87 × 10^−1^
rs118163260	A/T	T	0.003	−764.3 (3,777.2)	[−2,766.2, 8,006.3]	7.32 × 10^−1^
ss947846714	A/G	G	0.003	−664.9 (3,715.4)	[−2,733.3, 8,235.9]	7.68 × 10^−1^

HMW adiponectin in serum was measured for the study subjects with available stored HYPEST serum samples (*n* = 184) using Human HMW Adiponectin/Acrp30 ELISA assay (R&D Systems)

*SE* standard error, *CI* confidence interval

^a^Major/minor alleles were defined according to UCSC Genome Browser (GRCh37/hg19)

^b^Effects and *P* values are calculated using linear regression, additive model including age, gender, BMI and ELISA plate as covariates in the model. Estimated significance threshold after correction for multiple testing *P* < 4.55 × 10^−3^

Additionally, two common *CDH13* SNPs (*r* ^2^ = 0.65) were associated with significantly lower adiponectin level: rs8060301 [T/A, A-allele = 54.2 %, *P* = 1.63 × 10^−3^, β (SE) = −955.2 (273.2) ng/mL] and rs12444338 [G/T, T-allele = 59.9 %, *P* = 2.13 × 10^−2^, β (SE) = −682.1 (278.3) ng/mL; Table 2]. These two SNPs are in strong LD (*r* ^2^ = 0.67–0.98; Table S4) with rs3865188 (9.7 kb upstream of *CDH13*) and rs4783244 SNPs (455 bp from CpG island) previously associated with serum adiponectin (Jee et al. 2010; Chung et al. 2011). Both rs8060301 and rs12444338 were shown to affect methylation of neighboring CpG sites in the HYPEST–CADCZ and EGCUT datasets (Tables 2, 3).

Four identified meQTLs in the *CDH13* promoter represented common SNPs (rs8060301, rs1244438, rs62040565, rs12443878) with genotype and phenotype data available in HYPEST, EGCUT and CADCZ and enabled us to perform a meta-analysis (*n* = 523) of association tests for serum lipids (total cholesterol, LDL, HDL, triglycerides) and blood pressure (BP). The meQTL rs8060301 exhibited a suggestive pleiotropic effect on HDL and DBP (nominal *P* < 0.005; Table 5). It is noteworthy, that all SNPs showed a trend for association with serum HDL.

**Table 5:** Results of HYPEST–CADCZ–EGCUT meta-analysis for genetic association tests between common SNPs within the *CDH13* promoter region and cardiometabolic traits

SNP	Test allele	Phenotype	Unit	HYPEST		CADCZ		EGCUT		Meta-analysis^a^	
				β (SE) [95 % CI]	*P* value	β (SE) [95 % CI]	*P* value	β (SE) [95 % CI]	*P* value	β (SE) [95 % CI]	*P* value
rs8060301	A	HDL	mmol/L	0.06 (0.04) [−0.15, 0.03]	1.77 × 10^−1^	0.09 (0.04) [0.01, 0.16]	2.54 × 10^−2^	0.07 (0.04) [−0.01, 0.15]	5.82 × 10^−2^	0.07 (0.02) [0.03, 0.12]	1.25 × 10^−3^
		DBP	mmHg	0.60 (1.65) [−3.84, 2.64]	7.17 × 10^−1^	−3.45 (1.29) [−6.00, −0.92]	8.34 × 10^−3^	−2.31 (1.10) [−4.47, −0.14]	3.69 × 10^−2^	−2.10 (0.75) [−3.56, −0.63]	4.97 × 10^−3^
		SBP	mmHg	−1.18 (2.52) [−3.75, 6.12]	6.39 × 10^−1^	−3.87 (2.17) [−8.12, 0.37]	7.58 × 10^−2^	−1.71 (1.60) [−4.86, 1.45]	2.87 × 10^−1^	−2.20 (1.15) [−4.45, 0.04]	5.43 × 10^−2^
rs12444338	T	HDL	mmol/L	0.04 (0.04) [−0.13, 0.05]	3.67 × 10^−1^	0.09 (0.04) [0.02, 0.16]	1.44 × 10^−2^	0.08 (0.04) [0.00, 0.15]	4.99 × 10^−2^	0.07 (0.02) [0.03, 0.12]	1.51 × 10^−3^
rs62040565	C	HDL	mmol/L	0.06 (0.04) [−0.14, 0.02]	1.41 × 10^−1^	0.07 (0.04) [−0.01, 0.14]	8.88 × 10^−2^	0.03 (0.05) [−0.06, 0.12]	5.10 × 10^−1^	0.06 (0.02) [0.01, 0.10]	2.36 × 10^−2^
rs12443878	A	HDL	mmol/L	0.06 (0.04) [−0.14, 0.03]	1.94 × 10^−1^	0.05 (0.04) [−0.03, 0.12]	2.24 × 10^−1^	0.03 (0.04) [−0.05, 0.11]	4.13 × 10^−1^	0.04 (0.02) [−0.01, 0.09]	5.48 × 10^−2^

*SE* standard error, *CI* confidence

^a^Only associations, which demonstrated enhanced significance in meta-analysis compared to all sample-specific tests, are given. Effects and *P* values are calculated using linear regression, including age and gender as covariates in the model; results were combined using the inverse-variance method under a fixed-effects model. Estimated significance threshold after correction for multiple testing *P* < 8.33 × 10^−4^

### No significant effect of *CDH13* promoter CpG methylation level on cardiometabolic traits

The effect of methylation levels at the individual CpG sites within the *CDH13* promoter (HYPEST, CADCZ: *n* = 46 CpG sites/units) was assessed on serum lipid and BP levels. Although 16 of 276 conducted tests reached nominal *P* < 0.05 (Table S8), no associations remained significant after multiple testing correction and none of the involved CpG sites were identified to be modulated by meQTLs.

## Discussion

We hypothesized that the pleiotropy and heterogeneity of genetic associations in *CDH13* with a number of cardiometabolic traits might reflect interplay of inter-individual differences in DNA methylation variation. It has recently been shown that genetic variability extensively impacts DNA methylation (Shi et al. 2014). Consistent with the study hypothesis, several SNPs in the *CDH13* promoter region were significantly associated with the level of DNA methylation at nearby CpG sites (Tables 2, 3, 6; Fig. 3). These genetic variants showed simultaneous association signals with serum HMW adiponectin and lipid levels and were in LD with previously reported GWAS hits (Fig. 1; Tables 4, 5, 6).

**Table 6:** Summary results of the study

Analysis/trait	Study group	Significance level after correction for multiple testing	SNP showing statistically significant or suggestive for association
***A*** *. meQTL identification*			
CpG methylation	HYPEST–CADCZ meta-analysis^a^	*P* < 7.76 × 10^−5^	rs113460564^d^, rs12443878, rs12444338, rs62040565, **rs8060301**, rs2239857
	EGCUT^b^	*P* < 7.53 × 10^−4^	rs12443878^d^, rs12444338^d^, rs62040565^d^, **rs8060301** ^d^
***B*** *. SNP and trait association testing*			
Adiponectin^c^	HYPEST	*P* < 4.55 × 10^−3^	**rs8060301** ^d^, rs2239857^d^, rs77068073^d^, rs12444338
HDL	HYPEST–CADCZ–EGCUT meta-analysis	*P* < 8.33 × 10^−4^	rs12443878, rs12444338, rs62040565, **rs8060301**
SBP, DBP	HYPEST–CADCZ–EGCUT meta-analysis	*P* < 8.33 × 10^−4^	**rs8060301**
***C*** *. DNA methylation*-*trait association*			
Methylation level of individual *CDH13* promoter CpGs was not significantly modulating cardiometabolic traits			

SNP rs8060301 overlapping between the analysis results, is highlighted in bold

^a^Genotype data: targeted resequencing of the *CDH13* promoter region; CpG methylation data: EpiTYPER™ assay covering majority of the CpG sites

^b^Genotype data: HumanOmniExpress BeadChips (Illumina); CpG methylation data: selected CpG sites at the Infinium HumanMethylation450 BeadChips

^c^HMW adiponectin measurements were only available for HYPEST

^d^Significant after multiple testing correction

Among the identified meQTLs, rs8060301 located at the edge of the *CDH13* CpG island within intron 1 modulated significantly DNA methylation in both study samples HYPEST/CADCZ and EGCUT (Table 6). Simultaneously, it exhibited the strongest genetic associations among the tested *CDH13* SNPs with HDL (*P* = 1.25 × 10^−3^) and blood pressure levels (DBP = 4.97 × 10^−3^; SBP = 5.43 × 10^−2^) in the HYPEST–CADCZ–EGCUT meta-analysis. Association testing with serum HMW adiponectin in HYPEST highlighted rs8060301 as a common SNP (tested A-allele frequency 54.2 %) with the most significant and notable effect on this trait [*P* = 1.63 × 10^−3^, β (SE) = −955.2 (273.2) ng/mL]. Previously mapped serum adiponectin GWAS hit in Han Chinese (Chung et al. 2011) and Filipinos (Wu et al. 2010) rs4783244 is located only 524 bp from rs8060301 and these SNPs are in strong LD (Fig. 1; Table S4). However, only the rs8060301 (and not rs4783244) is located in the middle of the strongest binding site of RNA polymerase II (Pol2) within the *CDH13* promoter (ENCODE Project Consortium 2012) and can thus exhibit direct effect on gene expression. The effect on the gene expression level of *CDH13* may lead to altered protein expression and the affected sufficiency/abundance of T-cadherin molecules to bind adiponectin and lipids. Further functional studies need to be conducted to directly address its potential effect on *CDH13* transcription level.

We used a complementary approach to identify meQTLs, which has both advantages and limitations. The HYPEST/CADCZ cardiovascular diseases samples were targeted to fine scale analysis of CpG sites at the *CDH13* promoter using resequencing and the EpiTYPER™ assay. Using more dense assays provides additional value enabling to identify SNP–CpG associations that are not included in genome-wide assays. Data for a population-based cohort (EGCUT) were extracted from DNA methylation and genotyping arrays with highly standardized experimental and analytical methods. However, DNA methylation and genotyping chips do not cover dense runs of CpG sites within the CpG islands and rare SNPs, respectively. Thus, it was not possible to directly replicate the identified HYPEST/CADCZ top SNP–CpG site association pairs in EGCUT, although the same SNPs were confirmed as meQTLs in both study samples. An additional limitation in our study design was the not perfectly matched sample sets. Although both derived from Eastern/Central Europe, HYPEST (Estonia)/CADCZ (Czech) subjects have been recruited based on CVD (characterized by hypertension, CAD, MI), but EGCUT (Estonia) is a population-based cohort. This may introduce SNP-independent effects on the DNA methylation profiles and weaken the meQTL analysis.

GWAS studies have mapped the strongest genetic associations in the *CDH13* gene with serum adiponectin levels. In the current study, we measured serum HMW adiponectin levels only for 184 HYPEST subjects and identified significant associations with rs8060301 and rs12444338 (Table 4), which are in strong LD with previously detected SNPs in GWASs [rs3865188 (Jee et al. 2010); rs4783244 (Chung et al. 2011)]. Additionally, our resequencing approach allowed identification of two novel rare (MAF < 5 %) variants (rs2239857 and rs77068073) with a highly significant, strong effect on serum HMW adiponectin (Fig. 3). Notably, rs2239857 also exhibited potential as a meQTL for 3 CpG sites (Table 2).

We did not identify any significant associations between DNA methylation levels in the *CDH13* promoter and blood pressure or lipid levels. Alternative reasons (each one separately and also cumulatively), which may have affected achieving sufficient statistical power in testing these associations could be cross-sectional (whole blood cells) measurement of DNA methylation and its narrow range of variability within the promoter region, heterogenous study sample with regard to other variables modulating DNA methylation (e.g., age) and also possibly insufficient sample size failing to detect real correlations. In addition, investigation of direct effect of DNA methylation on cardiovascular phenotype trait would be more relevant using tissues with high and specific expression of *CDH13*, such as endothelium. In general, our study indicates that effects of SNPs on studied cardiometabolic traits could be primary, but due to strong meQTLs in the region, we suggest that these effects are dependent on DNA methylation levels, which further modulate the SNP trait effects.

DNA methylation changes in the *CDH13* promoter region are well characterized in the development of various cancers. In perspective, the meQTLs identified in this study in the context of CVD could be further subjected to studies of cancer patients. We speculate that meQTLs in the *CDH13* promoter may serve as potential prognostic markers for an increased risk to trigger more extensive changes in the DNA methylation pattern. Recently, a comprehensive meQTL catalog was published containing DNA methylation associations for 21 % of interrogated cancer risk polymorphisms (Heyn et al. 2014).

In summary, our study shows conclusively that the *CDH13* promoter harbors meQTLs associated with cardiometabolic traits. It paves the way to deeper understanding of the interplay between DNA variation and methylation in susceptibility to common diseases.

## Electronic supplementary material

Below is the link to the electronic supplementary material.

Supplementary material 1 (PDF 1423 kb)
