# Supplementary material for: CDH13 promoter SNPs with pleiotropic effect on cardiometabolic parameters represent methylation QTLs
Source: Hum Genet. 2014 Dec 28;134(3):291–303. doi: 10.1007/s00439-014-1521-6 (PMC4318987; doi:10.1007/s00439-014-1521-6)
Supplement: Supplementary file 1 — Supplementary material 1 (PDF 1423 kb) [file 439_2014_1521_MOESM1_ESM.pdf]

SUPPLEMENTAL MATERIAL

Title: *CDH13 promoter SNPs with pleiotropic effect on cardio-metabolic parameters represent methylation-QTLs*

Authors: Margus Putku[1], Mart Kals[2,3], Rain Inno[1], Silva Kasela[2,4], Elin Org[1,5], Viktor Kožich[6], Lili Milani[2], Maris Laan[1]

Affiliations:

[1]Human Molecular Genetics Group, Institute of Molecular and Cell Biology, University of Tartu, Tartu, Estonia; [2]Estonian Genome Center, University of Tartu, Tartu, Estonia; [3]Institute of Mathematical Statistics, University of Tartu, Tartu, Estonia; [4]Department of Biotechnology, Institute of Molecular and Cell Biology, University of Tartu, Tartu, Estonia; [5]Department of Medicine/Division of Cardiology, David Geffen School of Medicine, UCLA, Los Angeles,USA; [6]Institute of Inherited Metabolic Diseases, Charles University – First Faculty of Medicine, Prague, Czech Republic

Correspondence and request for reprints:

Maris Laan, Institute of Molecular and Cell Biology, University of Tartu, Riia St. 23, Tartu 51010, Estonia; Tel: +372-7375008; Fax: +372-7420286; E-mail: maris.laan@ut.ee

List of Supplemental Material:

Supplemental Methods

Study inclusion criteria, measurement of blood pressure and serum lipids in HYPEST/CADCZ participants

Measurement of serum HMW adiponectin in HYPEST

Data collection, blood pressure and lipid measurements in EGCUT participants

Assessment of suitability of DNA from whole blood for CpG-methylation profiling of *CDH13*

Quality control of CpG methylation measurements in HYPEST/ CADCZ

Long-range PCR for CDH13 promoted region amplification

Data pre-processing and QC of genome-wide DNA methylation arrary dataset of EGCUT samples

Assessment of age as a confounder of DNA methylation level in *CDH13* promoter region

Genotyping and association testing of the GT-microsatellite located within the *CDH13* promoter CpG island

Supplemental Table S1 Sequences of oligonucleotide primers

Supplemental Table S2 CpG-sites targeted for methylation measurement with Sequenom MassARRAY EpiTYPER^{TM} application

Supplemental Table S3 CpG sites overlapping in HYPEST, CADCZ and EGCUT datasets

Supplemental Table S4 LD (r^2) between common SNPs identified by resequencing in HYPEST and CADCZ and previously known SNPs associated with cardio-metabolic traits using EGCUT genotype data

Supplemental Table S5 Assessment of age as a potential covariate affecting DNA methylation level - association testing between *CDH13* DNA methylation at 46 CpG sites/units with age among HYPEST and CADCZ study subjects

Supplemental Table S6 SNP-s identified by promoter region resequencing

Supplemental Table S7 Genetic association between four overlapping common SNPs identified in HYPEST -CADCZ study groups and DNA methylation in the ECGUT sample-set

Supplemental Table S8 Results of HYPEST-CADCZ meta-analysis for association tests between the DNA methylation level at targeted *CDH13* promoter CpG sites/units and cardio-metabolic traits

Supplemental Figures and legends

Supplemental Figure S1 Overview of the study data and analysis

Supplemental Figure S2 *CDH13* DNA methylation in different blood cell types

Supplemental Figure S3 *CDH13* promoter region targeted to DNA methylation analysis

Supplemental Figure S4 Distribution of microsatellite length variants in HYPEST and CADCZ

References to Supplemental Methods

SUPPLEMENTAL METHODS

Study inclusion criteria, measurement of blood pressure and serum lipids in HYPEST/CADCZ participants

HYPEST subjects (full sample: n=1,966; aged 18-85 yrs) have been recruited across Estonia during 2004-2007 by North Estonia Medical Center (Cardiology and Blood Centres), and by Tartu University Hospital (Cardiology Clinic, Blood Centre) with the main aim to analyze genetic-epidemiological risk factors for cardiovascular disease (CVD) in Estonian population (Org et al. 2011). The CADCZ subjects (full sample: n=869; 18-67 yrs) have been recruited across Czech Republic during 1998-2000 by the Cardiology Department of the 2nd Clinic of Internal Medicine, Faculty Hospital Královské Vinohrady in Prague with the main aim to study genetic factors relating to homocysteine metabolism in coronary artery disease (CAD) (Janosíková et al. 2003). For the current study, the HYPEST and CADCZ patients were selected based on the following criteria: (i) clinically diagnosed CVD (MI, CAD, hypertension) or (ii) determined metabolic risk-factors CVD (serum LDL >3 mmol/L, total cholesterol >5 mmol/L; blood pressure >140/90 mmHg).

For all HYPEST and CADCZ subjects resting blood pressure (BP) had been measured by trained clinicians during recruitment. In both studies three BP measurements per subject were obtained after a rest in a sitting position using a standard mercury column sphygmomanometer and size-adjusted cuffs and the median value was recorded. Although longitudinal clinical data for past BP measurements is available for HYPEST subjects (Org et al. 2011), the current study used BP values documented at the recruitment prior the blood draw for DNA extractions. This allowed correct incorporation of subject's age as a potential confounder for methylation level of some CpG sites (Bell et al. 2012; Gomes et al. 2012).

Biochemical parameters (total cholesterol, LDL, HDL and triglycerides) from fasting serum of each participant were measured at the recruitment. Clinical chemistry assays for HYPEST samples were performed using standardized assays (Cobas Integra 800® analytical platform, Roche Diagnostics, Inc. USA) applied at the accredited hospital laboratories in Tartu (United Laboratories, Tartu University Clinics) and in Tallinn (Diagnostic Division Laboratory, North Estonia Medical Center). For CADCZ, serum lipids were measured by routine diagnostics assays at Faculty Hospital Královské Vinohrady, Institute of Clinical Chemistry.

Among 192 HYPEST and 166 CADCZ subjects 15.6% and 32.5% received lipid-lowering medications, and 86.4% and 25.5% antihypertensive treatment, respectively. For the subjects under treatment, corrected values for blood lipids and for BP were used in subsequent genetic data analysis. Serum lipid levels were corrected according to Wu et al. (2007) (total cholesterol +1.31 mmol/L; HDL -0.059 mmol/L; LDL +1.243 mmol/L; triglycerides +0.222 mmol/L) and BP values according to Tobin et al. (2005) (SBP +15 mmHg; DBP +10 mmHg).

Measurement of serum HMW adiponectin in HYPEST

Serum samples of HYPEST subjects were stored at -86 ^{o}C immediately after blood draw. The concentration of high molecular weight (HMW) adiponectin in serum was measured for the study subjects with available stored serum samples (n=184) using Human HMW Adiponectin/Acrp30 ELISA assay (R&D Systems) protocol as recommended by the manufacturer. HMW Adiponectin concentration in serum samples was measured in duplicate on one ELISA reaction plate and the average of two measurements was calculated and used in the following association analysis. Intra- and interassay coefficients of variation were < 5% and < 15%, respectively.

Data collection, blood pressure and lipid measurements in EGCUT participants

The population-based biobank of the Estonian Genome Center of the University of Tartu (EGCUT, http://www.geenivaramu.ee/en/) includes epidemiological-clinical datasets and DNA samples extracted from blood for Estonian adults across all age groups (n=51,515; Leitsalu et al. 2014). Each participant filled out a Computer Assisted Personal interview (http://www.geenivaramu.ee/sites/default/files/geenivaramu/ksimustiku_tutvustus.pdf) during 1-2 hours at a recruitment office, including personal data, genealogical data, lifestyle data and medical history (diseases recorded in ICD-10 systems and the use of medicaments). Height, weight and BP phenotypes (SBP and DBP) were measured at the recruitment office according to standard medical procedures. BP was measured three times per subject after a rest in a sitting position and the median values were used in the analysis. Serum lipid parameters (total cholesterol, LDL, HDL and triglycerides) were assessed from fresh blood at the United Laboratories, Tartu University Clinics using identical assays and platforms as for the HYPEST samples. The whole project is conducted according to the Estonian Genes Research Act (http://www.geenivaramu.ee/for-scientists/human-genes-research-act.html; Dec 13th, 2000) and all participants have signed the informed consent.

In the current study, *in silico* data for the *CDH13* region was extracted and analyzed for the EGCUT samples (n=165; aged 18-84; 41.6±22.7 years) with available datasets for both, genome-wide DNA methylation (Infinium HumanMethylation450 BeadChip, Illumina) and genome-wide imputed genotypes (HumanOmniExpress BeadChips, Illumina). Among the included 165 EGCUT subjects 5.5% received lipid-lowering and 24.2% antihypertensive treatment. In the statistical analyses, serum lipid and BP values of these individuals were corrected using the identical approach as described for the HYPEST and CADCZ subjects.

Assessment of suitability of DNA from whole blood for CpG-methylation profiling of *CDH13*

Whole blood consists of many different cell types, which may be characterized by differential CpG-methylation at several loci (Adalsteinsson et al. 2012). In order to assess the suitability of DNA extracted from whole blood for the reliable CpG methylation profiling at the *CDH13* locus, a publicly available genome-wide DNA methylation dataset for purified human blood cells was used (Reinius et al. 2012; http://publications.scilifelab.se/kere_j/methylation). This dataset comprises of DNA methylation profiles of six healthy male subjects determined for whole blood, mononuclear cells, granulocytes, and cells from seven selected purified lineages. DNA methylation analysis was performed with the Infinium Human Methylation 450K bead chip technology (Illumina, USA). For the current study DNA methylation values for 69 CpG sites present at the 450K chip for the *CDH13* genic region (GRCh37/hg19, Chr16:82,638,798 – 83,838,786) were comparatively assessed in different blood cell types and whole blood (**Figure S2**). All analyzed blood cell types demonstrated similar CpG methylation profile across the entire *CDH13* promoter and genic region, supporting the suitability of using whole blood cells for the inter-individual DNA methylation studies of *CDH13*.

Experimental and analytical details, and quality control of CpG methylation measurements in HYPEST/ CADCZ

Four EpiTYPER^{TM} assays were designed to cover 110 CpG sites (13-40 CpG-sites/assay) within a ~1.5 kb target region (1162 bp; GRCh37/hg19, Chr16: 82,660,652 – 82,661,813; **Figure S3**) using the EpiDesigner software as instructed by the manufacturer. The sequence context (e.g. presence of GT-microsatellite repeats) did not allow the inclusion of six CpG-sites. Genomic DNA (400 ng) extracted from whole blood was bisulfite treated using the EZ-

96 DNA Methylation™ kit (Zymo Research). Converted DNA was amplified (length of PCR products 289-470 bp) with primers (**Table S1)** containing T7-promoter tag and treated with shrimp alkaline phosphatase (Sequenom Inc.). Purified products were *in vitro* transcribed and fragmented using T-specific cleavage. Fragments (1-57 bp) were analyzed with MassARRAY Analyzer 4 (Sequenom Inc.), and methylation values were calculated by EpiTYPER Analyzer v.1.0 (Sequenom Inc.). Fragments containing >1 CpG sites were named as CpG-units (60 CpG-sites formed 21 CpG-units with 2-6 CpG sites/unit) and the methylation value for a CpG-unit was calculated as average methylation across the CpG-sites forming a unit. The remaining 50 CpG-sites within the targeted region contained 1 CpG-site per fragment and were assessed individually.

EpiTYPER Analyzer assesses the quality for every measured mass spectrum representing one EpiTYPER™ assay, and confidence values (CV; 0 = low and 5 = high confidence, respectively) for the spectrums are calculated. Confidence is calculated by analyzing the recalibration of the spectrum, the number of missing signals, and the number of additional signals. After empirical estimation, only assays with CV≥3 (88.4% across all assays and analyzed individuals) were included for subsequent analysis. The challenge of the EpiTYPER™ assay method is reliable measurement of CpG-methylation for the genomic regions with a high density of CpG sites, such as *CDH13* promoter CpG-island (**Figure S3**). During Quality Control (QC), EpiTYPER™ Analyzer program automatically excluded from methylation calculations the fragments with too low (<1500 Da) or too high (>7000 Da) mass for technically reliable in mass-spectrometry measuring range (20 CpG-sites). Fragments with indistinguishable mass or low CpG-methylation call rate were excluded from statistical tests (24 CpG-sites).

Protocol for DNA methylation detection by the EpiTYPER^{TM} assay is detailed in (http://bioscience.sequenom.com/sites/bioscience.sequenom.com/files/EpiTYPER%20Application%20Note.pdf). Its principles are described by Ehrich et al (2005).

Long-range PCR for *CDH13* promoted region amplification

Long-range PCR (2,602 bp) was carried out in a 40 µl reaction mixture containing 0.25 mM of each dNTPs (Solis BioDyne, Estonia), 1X Long PCR buffer, 1.5 mM $MgCl_2$ and 1.25 units of Long PCR enzyme mix (Thermo Fisher Scientific, Lithuania), 0.125 µM of both primers (forward: 5'-TGGTCCATTGCTCAGTGTCCTGC-3', reverse 5'-TGCACCAGTCTCCCCATCCCA-3'), 2.5% dimethyl sulfoxide and 100 ng of template DNA. The amplification was performed in GeneAmp 2700 (Applied Biosciencies, USA) instruments under following conditions: initial denaturation at 95°C for 3 min, followed by 10 'touchdown' cycles of 95°C for 15s, and annealing from 66°C to 57°C for 30s, and 68°C for 2.5 min, then an additional 25 cycles of 95°C for 15s, 56°C for 30s and 68°C for 2.5 min. Starting from the 11th cycle the extension time was prolonged by 1s for each remaining cycle. The reaction was ended by final incubation of 68°C for 10 min.

Genome-wide DNA methylation array dataset of EGCUT samples

For genome-wide DNA methylation profiling of EGCUT samples (n=165), 500 ng of each genomic DNA was bisulfite modified using the EZ DNA Methylation kit (Zymo Research, Cat. # D5004) according to the manufacturer's recommendations for the Illumina Infinium Assay. After purification, 4 µl of each bisulfite-converted DNA sample was used for amplification and hybridization on Infinium HumanMethylation450 BeadChips (Illumina Infinium HD Methylation protocol). The original IDAT files were extracted from the HiScanSQ scanner. Data pre-processing and QC analyses were performed in R using the Bioconductor package *minfi* version 3.0.1 (Aryee et al. 2014;

http://bioconductor.org/packages/release/bioc/vignettes/minfi/inst/doc/minfi.pdf). 'Raw' pre-processing was used to convert the intensities from the red and the green channels into methylated and unmethylated signals. Beta values were computed using Illumina's formula [beta = M/(M+U+100)]. The difference in the distribution of beta values for type I and type II probes was corrected for using the SWAN normalization method (Maksimovic et al. 2012). Detection *P*-values were obtained for every genomic position in every sample. Failed positions were defined as signal levels lower than background from both the methylated and unmethylated channels. Samples with non-significant detection p-values (> 0.01) in more than 10% of the probes were discarded. Using MixupMapper we also corrected for mixups in the methylation data (Westra et al. 2011). After SWAN normalization Beta-values were transformed to M-values: M = $\log_2$(beta/1-beta). Further, we applied a principal component analysis (PCA) on the methylation correlation matrix to correct for physiological or environmental variation (e.g. phenotype difference) as well as systematic experimental variation (e.g. batch and technical effect). In order to target the difference in the genetic variation of methylation, we removed the global variation in methylation by using the residual methylation for each probe after removing the optimal number of PCs. This optimum was determined based on the maximum number of *cis*-meQTLs.

Imputation of genome-wide genotyping data in EGCUT

Imputing of unobserved genotypes was implemented in IMPUTE v2 (Howie et al. 2009) using The 1000 Genomes Project sequencing data (Phase 1) as a reference panel (Abecasis et al. 2012; http://www.1000genomes.org/). In the EGCUT dataset, the directly genotyped SNPs were coded as 0, 1 or 2, while the imputed SNP dosage values were called at a 0.95 confidence level, ranging between 0 and 2. SNPs with an imputation quality <0.4.

Assessment of age as a confounder of DNA methylation level in *CDH13* promoter region

Prior association testing with genetic and cardio-metabolic parameters, the subject's age was assessed as a potential covariate modulating DNA methylation at the studied 46 CpG sites/unites in HYPEST and CADCZ. Results of association testing with age (linear regression; gender, experiment series as cofactors) were combined in a meta-analysis using the inverse-variance method under a fixed-effects model implemented in R, ver. 3.0.2 (R Development Core Team 2014, http://www.r-project.org/). None of the CpG methylation – age nominal associations (P<0.05) remained significant after multiple testing correction (α=5.4x10^{-4}) (**Table S5**).

Analysis of the GT-microsatellite located within the *CDH13* promoter CpG island

The CpG island of *CDH13* harbors a polymorphic GT-microsatellite (hg19, Chr16: 82,660,943-82,660,986). Fragment analysis was performed for the accurate determination of the length of microsatellite alleles for the resequenced HYPEST and CADCZ subjects (n=358). PCR reaction (15 μl) contained 0.25 mM of each dNTPs, 1X B1 PCR buffer, 2.5 mM $MgCl_2$ and 0.375 units of HOT FIREPol DNA Polymerase (Solis BioDyne, Estonia), 0.25 μM of both primers (forward: 5'-6-FAM-CCCGTTTCTGCATTCGGATC-3', reverse 5'-TTGATGTGGGCGAGAATGTC-3') and 75 ng of template DNA. The microsatellite amplification was performed under following conditions: initial denaturation at 95°C for 13 min, followed by 10 'touchdown' cycles of 95°C for 30s, and annealing from 60°C to 51°C for 30s, and 72°C for 1 min, then an additional 25 cycles of 95°C for 30s, 50°C for 30s and 72°C for 1 min. The reaction was ended by final incubation of 72°C for 10 min. GeneAmp 2700 (Applied Biosciencies, USA) instruments were used. PCR products were diluted in formamide, mixed with the GeneScan™ 600 LIZ® size standard and analyzed for the fragment length with 3730xl DNA Analyzer (Applied Biosystems).

In subsequent data analysis the study subjects were divided into two groups based on the determined length of their microsatellite alleles (**Figure S4**). Individuals carrying one or two extremely long (≥299 bp; HYPEST, n=18; CADCZ, n=29) alleles formed the 'long allele' group, subjects with two <299 bp alleles formed the 'normal allele' group. The effect of the microsatellite length on methylation level on neighboring CpG sites/unites was tested using logistic regression implemented in R software. Age and gender were used as covariates in all analyses. Length of the microsatellite showed no evidence of association with DNA methylation (data not presented).

Table S1. Sequences of oligonucleotide primers

	Primer ID	Sequence 5´- 3´[a]	PCR product length (bp)	CpG-site coverage
**A**. DNA methylation assays	CDH13_assay1_F	aggaagagagTTTTTTTGTATTAGGTTGGAAGTGG	289	CpG_1-13
	CDH13_assay1_R	cagtaatacgactcactatagggagaaggctCCAAATAAATCAACAACAACATCAC		
	CDH13_assay2_F	aggaagagagGGGAGGAGGAGAGAGTTTTTAG	470	CpG_51-90
	CDH13_assay2_R	cagtaatacgactcactatagggagaaggctAAACTCCAACTAACTAAACAATAAAAATA		
	CDH13_assay3_F	aggaagagagGTTAGAGTGGGAGTTTGTGTGTGG	339	CpG_15-45
	CDH13_assay3_R	cagtaatacgactcactatagggagaaggctACAAAACCTCTCCTCAAAACCTAAC		
	CDH13_assay4_F	aggaagagagGGTTTTGTTTTGTTTTGGTTTTTATT	438	CpG_91-116
	CDH13_assay4_R	cagtaatacgactcactatagggagaaggctCACCCCTATTATCCAACCAACTAA		
**B**. Sequencing primers	CDH13_F1	TGGTCCATTGCTCAGTGTCCTGC[b]	2602	NA
	CDH13_R1	TGCACCAGTCTCCCCATCCCA[b]		
	CDH13_seqF2	CAGCTTTGGTGCCAGATGCACA		
	CDH13_seqF3	GCCATTGGTCCTGGTCATCAGCC		
	CDH13_seqF4	GGGTAACTTTGGTTCCCTCCTCCG		
	CDH13_seqF5	CTGCTGCCTTCCCTGAGCGG		
	CDH13_seqR2	TTTCTCTGCGCTCCGCTCCC		
	CDH13_seqR3	GCCCCGATCTGTCTTCGCCG		
	CDH13_seqR4	AATTGCACGGGGGACTGGCG		
	CDH13_seqR5	ACCTCCAGGTACTCAGAACAGCAG		

Methylation assays were designed using EpiDesigner software as instructed by the manufacturer. Sequencing primers were designed with MPrimer3 software (http://bioinfo.ut.ee/mprimer3/).

[a]Tags in lower case letters, forward 10mer tag to balance the PCR; reverse T7-promoter tag for in vitro transcription and 8bp insert to prevent abortive cycling

[b]Initial long-range PCR primers

Table S2. CpG-sites targeted for methylation measurement with Sequenom MassARRAY EpiTYPER™ application

CpG site/unit	Position on chr.16 GRCh37/hg19[a]	Final association testing	Reason for exclusion[b]
CpG_1	82,660,317	+	
CpG_2	82,660,328	+	
CpG_3	82,660,376	+	
CpG_4-8	82,660,432-82,660,450	-	Fragment with too high mass
CpG_9	82,660,464	+	
CpG_10	82,660,482	-	Fragment with too low mass
CpG_11	82,660,490	+	
CpG_12	82,660,505	+	
CpG_13	82,660,554	+	
CpG_14	82,660,596	-	Not covered by assays
CpG_15	82,660,630	+	
CpG_16-17	82,660,652-82,660,654	+	
CpG_18-21	82,660,670-82,660,680	-	Overlapping mass with CpG_33-35
CpG_22	82,660,690	+	
CpG_23	82,660,705	+	
CpG_24	82,660,714	-	Removed after QC
CpG_25	82,660,718	-	Fragment with too low mass
CpG_26	82,660,727	-	Fragment with too low mass
CpG_27-29	82,660,761-82,660,767	+	
CpG_30-31	82,660,774-82,660,779	+	
CpG_32	82,660,791	+	
CpG_33-35	82,660,796-82,660,805	-	Overlapping mass with CpG_18-21
CpG_36	82,660,826	+	
CpG_37	82,660,834	+	
CpG_38	82,660,843	+	

CpG_39-42	82,660,847-82,660,855	+	
CpG_43	82,660,866	+	
CpG_44	82,660,873	-	Fragment with too low mass
CpG_45	82,660,890	+	
CpG_46-50	82,660,938-82,660,998	-	Not covered by assays
CpG_51	82,661,041	-	Removed after QC
CpG_52	82,661,050	+	
CpG_53	82,661,087	+	
CpG_54	82,661,095	+	
CpG_55	82,661,125	-	Removed after QC
CpG_56	82,661,134	-	Fragment with too low mass
CpG_57-58	82,661,139-82,661,141	+	
CpG_59	82,661,159	-	Duplicate mass with CpG_82
CpG_60-61	82,661,166-82,661,170	-	Overlapping mass with CpG_65
CpG_62-64	82,661,175-82,661,188	+	
CpG_65	82,661,195	-	Overlapping mass with CpG_60-61
CpG_66	82,661,202	+	
CpG_67	82,661,217	+	
CpG_68	82,661,231	-	Fragment with too low mass
CpG_69-70	82,661,244-82,661,248	+	
CpG_71-72	82,661,267-82,661,275	+	
CpG_73-74	82,661,285-82,661,290	+	
CpG_75-76	82,661,313-82,661,326	+	
CpG_77-81	82,661,336-82,661,349	+	
CpG_82	82,661,355	-	Duplicate mass with CpG_59
CpG_83	82,661,360	+	
CpG_84-85	82,661,384-82,661,390	+	
CpG_86	82,661,399	+	

CpG_87-88	82,661,404-82,661,413	+	
CpG_89	82,661,421	+	
CpG_90	82,661,438	+	
CpG_91	82,661,469	+	
CpG_92	82,661,480	+	
CpG_93	82,661,488	-	Removed after QC
CpG_94-96	82,661,510-82,661,521	-	Fragment with too high mass
CpG_97	82,661,550	-	Removed after QC
CpG_98-103	82,661,580-82,661,622	-	Fragment with too high mass
CpG_104	82,661,633	+	
CpG_105	82,661,638	-	Removed after QC
CpG_106	82,661,670	+	
CpG_107	82,661,696	+	
CpG_108	82,661,701	+	
CpG_109	82,661,725	+	
CpG_110-111	82,661,730-82,661,739	-	Duplicate mass with CpG_113-114
CpG_112	82,661,746	-	Removed after QC
CpG_113-114	82,661,769-82,661,781	-	Duplicate mass with CpG_110-111
CpG_115	82,661,797	-	Removed after QC
CpG_116	82,661,812	+	

[a]In case of CpG-units, the positions of the first and last CpG-sites in the unit
[b]Described in Materials and methods

Table S3. CpG sites overlapping in HYPEST, CADCZ and EGCUT datasets

Position on chr.16 GRCh37/hg19	CpG code in HYPEST/CADCZ	CpG code in EGCUT
82,660,317	CpG_1	cg02069674
82,660,328	CpG_2	cg01880569
82,660,376	CpG_3	cg00423443
82,660,464	CpG_9	cg07301944
82,660,490	CpG_11	cg08977371
82,660,505	CpG_12	cg09825093
82,660,630	CpG_15	cg01301138
82,661,725	CpG_109	cg19369556

Table S4. LD (r^2) between common SNPs identified by resequencing in HYPEST and CADCZ and previously known SNPs associated with cardio-

SNP A	Position of SNP A[a]	SNP B	Position of SNP B	Trait SNP B has been associated with	r^2
rs12443878	82,659,933	rs10514542	82,557,684	total cholesterol level	0.00112203
rs12443878	82,659,933	rs11646213	82,642,651	hypertension, metabolic syndrome, pre-eclampsia	0.666202
rs12443878	82,659,933	rs12444338	82,660,155	hypertension	0.775768
rs12443878	82,659,933	rs12922394	82,672,327	adiponectin level	8.70E-06
rs12443878	82,659,933	rs4783244	82,662,268	adiponectin level	0.490769
rs12443878	82,659,933	rs62040565	82,660,946		0.554124
rs12443878	82,659,933	rs6565105	83,065,664	LDL level	0.00913971
rs12443878	82,659,933	rs7195409	83,527,592	adiponectin level	0.00484313
rs12443878	82,659,933	rs8055236	83,212,398	coronary artery disease	2.69E-05
rs12443878	82,659,933	rs8060301	82,661,744		0.473274
rs12443878	82,659,933	rs12596316	82,646,152	adiponectin level	0.663666
rs12443878	82,659,933	rs3852724	82,646,094	adiponectin level	0.223743
rs12443878	82,659,933	rs3865185	82,646,462	adiponectin level	0.223743
rs12443878	82,659,933	rs3865186	82,646,972	adiponectin level	0.215396
rs12443878	82,659,933	rs3865188	82,650,717	adiponectin level	0.797744
rs12443878	82,659,933	rs7193788	82,656,160	adiponectin level	0.268075
rs12443878	82,659,933	rs8053728	82,485,251	adiponectin level	0.000300612
rs12444338	82,660,155	rs10514542	82,557,684		0.0478803
rs12444338	82,660,155	rs11646213	82,642,651		0.485736
rs12444338	82,660,155	rs12922394	82,672,327		0.0338514
rs12444338	82,660,155	rs4783244	82,662,268		0.676577
rs12444338	82,660,155	rs62040565	82,660,946		0.439904
rs12444338	82,660,155	rs6565105	83,065,664		0.0132675
rs12444338	82,660,155	rs7195409	83,527,592		0.000325375
rs12444338	82,660,155	rs8055236	83,212,398		4.90E-07
rs12444338	82,660,155	rs8060301	82,661,744		0.651728
rs12444338	82,660,155	rs12596316	82,646,152		0.803393
rs12444338	82,660,155	rs3852724	82,646,094		0.0502016
rs12444338	82,660,155	rs3865185	82,646,462		0.0502016
rs12444338	82,660,155	rs3865186	82,646,972		0.0455023
rs12444338	82,660,155	rs3865188	82,650,717		0.950897

rs12444338	82,660,155	rs7193788	82,656,160	0.11109
rs12444338	82,660,155	rs8053728	82,485,251	0.00146287
rs62040565	82,660,946	rs10514542	82,557,684	0.000706188
rs62040565	82,660,946	rs11646213	82,642,651	0.392202
rs62040565	82,660,946	rs12596316	82,646,152	0.329218
rs62040565	82,660,946	rs3852724	82,646,094	0.141232
rs62040565	82,660,946	rs3865185	82,646,462	0.141232
rs62040565	82,660,946	rs3865186	82,646,972	0.128909
rs62040565	82,660,946	rs3865188	82,650,717	0.439904
rs62040565	82,660,946	rs12922394	82,672,327	0.00673134
rs62040565	82,660,946	rs4783244	82,662,268	0.276115
rs62040565	82,660,946	rs6565105	83,065,664	0.000287672
rs62040565	82,660,946	rs7195409	83,527,592	0.00190484
rs62040565	82,660,946	rs8055236	83,212,398	0.000379176
rs62040565	82,660,946	rs8060301	82,661,744	0.269388
rs62040565	82,660,946	rs7193788	82,656,160	0.128349
rs62040565	82,660,946	rs8053728	82,485,251	0.00247931
rs8060301	82,661,744	rs10514542	82,557,684	0.00781923
rs8060301	82,661,744	rs11646213	82,642,651	0.325643
rs8060301	82,661,744	rs12596316	82,646,152	0.528015
rs8060301	82,661,744	rs3852724	82,646,094	0.0214908
rs8060301	82,661,744	rs3865185	82,646,462	0.0214908
rs8060301	82,661,744	rs3865186	82,646,972	0.0194557
rs8060301	82,661,744	rs3865188	82,650,717	0.671102
rs8060301	82,661,744	rs7193788	82,656,160	0.0903346
rs8060301	82,661,744	rs8053728	82,485,251	6.74E-05
rs8060301	82,661,744	rs12922394	82,672,327	0.0482887
rs8060301	82,661,744	rs4783244	82,662,268	0.97755
rs8060301	82,661,744	rs6565105	83,065,664	0.00459503
rs8060301	82,661,744	rs7195409	83,527,592	0.00125748
rs8060301	82,661,744	rs8055236	83,212,398	0.0031027

[a] Genomic position on chromosome 16 (GRCh37/hg19).

Table S5. Assessment of age as a potential covariate affecting DNA methylation level - association testing between *CDH13* DNA methylation at 46 CpG sites/units with age among HYPEST and CADCZ study subjects.

		HYPEST				
CpG site/unit[a]	Position at chr.16[b]	Beta	SE	CI_2.5%	CI_97.5%	*P* -value[c]
CpG_1	82,660,317	0.123530606	0.111189983	-0.094397757	0.341458969	0.268363584
CpG_2	82,660,328	-0.075080105	0.113001526	-0.296559026	0.146398816	0.507464508
CpG_3	82,660,376	-0.01199093	0.086487661	-0.18150363	0.15752177	0.889921817
CpG_9	82,660,464	0.108083079	0.059695514	-0.008917978	0.225084137	0.072208292
CpG_11	82,660,490	0.094646396	0.121185098	-0.14287203	0.332164823	0.436107752
CpG_12	82,660,505	-0.007599618	0.042869718	-0.091622721	0.076423485	0.859531991
CpG_13	82,660,554	0.000882758	0.020352111	-0.039006647	0.040772162	0.965460882
CpG_15	82,660,630	0.053754357	0.024103988	0.006511409	0.100997305	0.027245789
CpG_16-17	82,660,652-82,660,654	-0.006805114	0.028139905	-0.061958314	0.048348086	0.80924552
CpG_22	82,660,690	0.071276043	0.036052175	0.000615079	0.141937007	0.049896832
CpG_23	82,660,705	0.024095765	0.018462135	-0.012089355	0.060280886	0.193868924
CpG_27-29	82,660,761-82,660,767	-0.023273906	0.032389211	-0.086755592	0.040207781	0.473578544
CpG_30-31	82,660,774-82,660,779	-0.005066314	0.007728085	-0.020213082	0.010080455	0.513117529
CpG_32	82,660,791	-0.060440848	0.036412146	-0.131807342	0.010925647	0.099306635
CpG_36	82,660,826	0.03578648	0.047330864	-0.056980308	0.128553268	0.450911463
CpG_37	82,660,834	-0.001127509	0.010483729	-0.021675241	0.019420223	0.914499514
CpG_38	82,660,843	0.044910455	0.056521296	-0.065869249	0.155690159	0.428186264
CpG_39-42	82,660,847-82,660,855	0.035404015	0.032413865	-0.028125992	0.098934023	0.276535068
CpG_43	82,660,866	-0.05743231	0.08742592	-0.228783965	0.113919346	0.5122491
CpG_45	82,660,890	-0.088744573	0.076630541	-0.238937672	0.061448527	0.248695321
CpG_52	82,661,050	-0.010720618	0.05295688	-0.114514196	0.09307296	0.83987268
CpG_53	82,661,087	-0.042906088	0.092189295	-0.223593786	0.137781611	0.643002611
CpG_54	82,661,095	-0.012946121	0.064825533	-0.14000183	0.114109589	0.842010141
CpG_57-58	82,661,139-82,661,141	0.02637258	0.108986585	-0.187237201	0.239982362	0.809176922
CpG_62-64	82,661,175-82,661,188	0.049730328	0.073710917	-0.094740415	0.19420107	0.50117436
CpG_66	82,661,202	0.117449905	0.062352684	-0.00475911	0.239658919	0.061749011
CpG_67	82,661,217	0.046485501	0.02523284	-0.002969956	0.095940958	0.067614777

CpG_69-70	82,661,244-82,661,248	-0.013126538	0.036979401	-0.085604833	0.059351757	0.723183715
CpG_71-72	82,661,267-82,661,275	-0.003647646	0.039489504	-0.081045652	0.07375036	0.926544098
CpG_73-74	82,661,285-82,661,290	0.00557398	0.023600005	-0.040681179	0.051829139	0.813652038
CpG_75-76	82,661,313-82,661,326	0.047108978	0.03702009	-0.025449065	0.119667022	0.205511581
CpG_77-81	82,661,336-82,661,349	0.056317666	0.036046039	-0.014331273	0.126966605	0.120556764
CpG_83	82,661,360	-0.011799148	0.127533568	-0.261760349	0.238162052	0.926425721
CpG_84-85	82,661,384-82,661,390	-0.075329113	0.113002855	-0.296810638	0.146152413	0.506201056
CpG_86	82,661,399	0.220611227	0.218574264	-0.207786459	0.649008913	0.314640596
CpG_87-88	82,661,404-82,661,413	-0.051502037	0.088167101	-0.224306379	0.121302305	0.560114775
CpG_89	82,661,421	-0.034253899	0.056669748	-0.145324564	0.076816766	0.546813769
CpG_90	82,661,438	-0.126867568	0.057456355	-0.239479954	-0.014255183	0.028902462
CpG_91	82,661,469	-0.076579185	0.14964965	-0.369887109	0.216728739	0.610220156
CpG_92	82,661,480	0.019486136	0.030777593	-0.040836838	0.07980911	0.528412307
CpG_104	82,661,633	0.109957763	0.128097688	-0.141109092	0.361024618	0.393274147
CpG_106	82,661,670	-0.087158964	0.096080766	-0.275473805	0.101155877	0.366989477
CpG_107	82,661,696	-0.070606906	0.058136514	-0.18455238	0.043338567	0.228128904
CpG_108	82,661,701	-0.463092994	0.2904138	-1.032293584	0.106107595	0.115182989
CpG_109	82,661,725	0.039740901	0.039539897	-0.037755874	0.117237675	0.317813354
CpG_116	82,661,812	0.005455072	0.194959513	-0.376658552	0.387568695	0.977745661

CpG site/unit[a]	Position at chr.16[b]	CADCZ Beta	SE	CI_2.5%	CI_97.5%	*P* -value
CpG_1	82,660,317	0.329957615	0.173750449	-0.010587008	0.670502238	0.059442587
CpG_2	82,660,328	0.139969192	0.169078201	-0.191417991	0.471356376	0.409052201
CpG_3	82,660,376	0.09231098	0.145642926	-0.193143909	0.377765869	0.527146586
CpG_9	82,660,464	0.062443943	0.223030098	-0.374687017	0.499574904	0.779868648
CpG_11	82,660,490	-0.060770043	0.154208542	-0.363013231	0.241473145	0.694087593
CpG_12	82,660,505	0.112517277	0.075742398	-0.035935095	0.26096965	0.139448841
CpG_13	82,660,554	-0.028860607	0.047894343	-0.122731795	0.065010582	0.547668408
CpG_15	82,660,630	-0.059502274	0.077367385	-0.211139562	0.092135015	0.443058894
CpG_16-17	82,660,652-82,660,654	-0.050058202	0.09285742	-0.232055402	0.131938997	0.590631604
CpG_22	82,660,690	0.013214173	0.097155727	-0.177207553	0.203635898	0.891996778
CpG_23	82,660,705	-0.06659232	0.118485131	-0.298818909	0.165634269	0.574939372
CpG_27-29	82,660,761-82,660,767	-0.160926048	0.066915554	-0.292078124	-0.029773971	0.017455547
CpG_30-31	82,660,774-82,660,779	-0.074858244	0.076950604	-0.225678657	0.075962168	0.332235473
CpG_32	82,660,791	-0.160373062	0.073567632	-0.304562971	-0.016183153	0.031022025
CpG_36	82,660,826	-0.062933298	0.077391099	-0.214617065	0.08875047	0.417463733
CpG_37	82,660,834	-0.026063414	0.023791734	-0.072694356	0.020567528	0.275073142
CpG_38	82,660,843	-0.265776556	0.103696875	-0.469018697	-0.062534416	0.011464392
CpG_39-42	82,660,847-82,660,855	-0.039816188	0.151539138	-0.336827441	0.257195064	0.793190122
CpG_43	82,660,866	-0.300541686	0.341168072	-0.96921882	0.368135447	0.379780007
CpG_45	82,660,890	-0.05364061	0.100071535	-0.249777215	0.142495996	0.592747579
CpG_52	82,661,050	-0.038764559	0.049636953	-0.136051198	0.058522081	0.436089474
CpG_53	82,661,087	0.145320805	0.087943907	-0.027046086	0.317687696	0.100771662
CpG_54	82,661,095	-0.046329359	0.050997963	-0.146283529	0.053624812	0.365134959
CpG_57-58	82,661,139-82,661,141	0.029236276	0.063153351	-0.094542018	0.153014569	0.644120354
CpG_62-64	82,661,175-82,661,188	0.023705278	0.057973856	-0.089921392	0.137331948	0.683259164
CpG_66	82,661,202	0.141924752	0.100254685	-0.05457082	0.338420324	0.159024647
CpG_67	82,661,217	0.07269912	0.08564236	-0.095156822	0.240555062	0.397373134

CpG_69-70	82,661,244-82,661,248	0.00852209	0.042217322	-0.074222341	0.09126652	0.840323735
CpG_71-72	82,661,267-82,661,275	-0.130233183	0.069281228	-0.266021895	0.00555553	0.062216707
CpG_73-74	82,661,285-82,661,290	-0.119861162	0.107391391	-0.33034442	0.090622095	0.266284832
CpG_75-76	82,661,313-82,661,326	0.049607158	0.07274165	-0.092963856	0.192178172	0.496439028
CpG_77-81	82,661,336-82,661,349	0.049828726	0.051970828	-0.052032224	0.151689677	0.339321126
CpG_83	82,661,360	0.061167682	0.109378999	-0.153211216	0.275546581	0.576875567
CpG_84-85	82,661,384-82,661,390	-0.06306398	0.098800648	-0.256709691	0.130581731	0.524309598
CpG_86	82,661,399	0.017066168	0.124375053	-0.226704457	0.260836794	0.891049609
CpG_87-88	82,661,404-82,661,413	0.014530789	0.096604858	-0.174811253	0.203872832	0.880649414
CpG_89	82,661,421	-0.056198673	0.077584798	-0.208262082	0.095864737	0.470067354
CpG_90	82,661,438	0.134279972	0.088100092	-0.038393036	0.306952979	0.129628148
CpG_91	82,661,469	-0.034654652	0.057472935	-0.147299534	0.077990231	0.547523256
CpG_92	82,661,480	-0.072778392	0.068709262	-0.20744607	0.061889287	0.291376104
CpG_104	82,661,633	-0.067476459	0.206436962	-0.472085469	0.337132552	0.744301801
CpG_106	82,661,670	-0.047909198	0.078000546	-0.200787459	0.104969062	0.540098602
CpG_107	82,661,696	-0.046729874	0.076916661	-0.197483759	0.10402401	0.544569568
CpG_108	82,661,701	0.102156128	0.189005497	-0.268287838	0.472600095	0.58978198
CpG_109	82,661,725	-0.230513844	0.13687443	-0.498782797	0.037755108	0.094451004
CpG_116	82,661,812	0.118161297	0.13672997	-0.14982452	0.386147114	0.389002666

		Meta-analysis[d]				
CpG site/unit[a]	Position at chr.16[b]	Beta	SE	CI_2.5%	CI_97.5%	*P* -value
CpG_1	82,660,317	0.183506079	0.093654667	-5.37E-05	0.367065853	0.050067055
CpG_2	82,660,328	-8.68E-03	0.093950367	-0.192820707	0.175457965	0.926377221
CpG_3	82,660,376	0.015201056	0.074364118	-0.130549937	0.160952049	0.838030105
CpG_9	82,660,464	0.105032078	0.05766564	-0.007990499	0.218054655	0.068546386
CpG_11	82,660,490	0.035310538	0.095283679	-0.151442041	0.222063117	0.710947937
CpG_12	82,660,505	0.021543367	0.037308359	-0.051579673	0.094666408	0.563641741
CpG_13	82,660,554	-0.003666376	0.018731086	-0.04037863	0.033045878	0.844815648
CpG_15	82,660,630	0.043733798	0.023012985	-0.001370824	0.08883842	0.057381097
CpG_16-17	82,660,652-82,660,654	-0.010447666	0.026930467	-0.063230411	0.04233508	0.698053286
CpG_22	82,660,690	0.064248667	0.033800126	-0.001998362	0.130495696	0.057322834
CpG_23	82,660,705	0.021946155	0.018241978	-0.013807464	0.057699775	0.228953912
CpG_27-29	82,660,761-82,660,767	-0.049402302	0.029153613	-0.106542333	0.007737729	0.090160509
CpG_30-31	82,660,774-82,660,779	-0.005767199	0.007691291	-0.020841852	0.009307455	0.453354135
CpG_32	82,660,791	-0.080104454	0.032633658	-0.144065248	-0.01614366	0.014101886
CpG_36	82,660,826	0.008913556	0.040378159	-0.070226181	0.088053293	0.825285611
CpG_37	82,660,834	-0.005184098	0.009593605	-0.023987218	0.013619022	0.588941792
CpG_38	82,660,843	-0.026250947	0.049627974	-0.123519989	0.071018095	0.59683692
CpG_39-42	82,660,847-82,660,855	0.032113069	0.031696906	-0.030011725	0.094237862	0.310998333
CpG_43	82,660,866	-0.072412715	0.084689481	-0.238401047	0.093575617	0.392530168
CpG_45	82,660,890	-0.075768905	0.060841152	-0.195015371	0.043477562	0.213000852
CpG_52	82,661,050	-0.025649144	0.036215482	-0.096630185	0.045331897	0.478798101
CpG_53	82,661,087	0.055641041	0.063633658	-0.069078637	0.180360719	0.381902426
CpG_54	82,661,095	-0.033567185	0.040081528	-0.112125536	0.044991166	0.402326921
CpG_57-58	82,661,139-82,661,141	0.02851645	0.054642457	-0.078580797	0.135613698	0.601758408
CpG_62-64	82,661,175-82,661,188	0.033651472	0.045568458	-0.055661065	0.12296401	0.460221787
CpG_66	82,661,202	0.124276494	0.052947604	0.020501096	0.228051891	0.018917134
CpG_67	82,661,217	0.048579263	0.02420412	0.00114006	0.096018466	0.044742646

CpG_69-70	82,661,244-82,661,248	-0.003728668	0.02781701	-0.058249006	0.05079167	0.893368784
CpG_71-72	82,661,267-82,661,275	-0.034690568	0.034307747	-0.101932517	0.03255138	0.311940597
CpG_73-74	82,661,285-82,661,290	-0.000208403	0.023049985	-0.045385543	0.044968737	0.992786133
CpG_75-76	82,661,313-82,661,326	0.047622935	0.032993155	-0.017042461	0.11228833	0.148902577
CpG_77-81	82,661,336-82,661,349	0.054210034	0.029619068	-0.003842272	0.11226234	0.067213899
CpG_83	82,661,360	0.0302431	0.083025981	-0.132484832	0.192971032	0.715663361
CpG_84-85	82,661,384-82,661,390	-0.068377806	0.07438012	-0.214160162	0.077404551	0.357937544
CpG_86	82,661,399	0.066852374	0.108099392	-0.145018542	0.278723289	0.536289083
CpG_87-88	82,661,404-82,661,413	-0.021494779	0.065122668	-0.149132863	0.106143305	0.741350086
CpG_89	82,661,421	-0.041888591	0.045762166	-0.131580788	0.047803605	0.360005706
CpG_90	82,661,438	-0.048939295	0.048126136	-0.143264789	0.045386198	0.30920271
CpG_91	82,661,469	-0.040043505	0.053652239	-0.145199962	0.065112951	0.455454254
CpG_92	82,661,480	0.00406708	0.028088374	-0.050985122	0.059119281	0.884872066
CpG_104	82,661,633	0.060631066	0.108845363	-0.152701926	0.273964057	0.577501076
CpG_106	82,661,670	-0.063501072	0.060557371	-0.182191339	0.055189195	0.294357601
CpG_107	82,661,696	-0.061925696	0.046378931	-0.152826731	0.028975338	0.181807034
CpG_108	82,661,701	-0.066025567	0.158411475	-0.376506352	0.244455218	0.676826251
CpG_109	82,661,725	0.018925199	0.037986661	-0.055527287	0.093377686	0.618338535
CpG_116	82,661,812	0.081004307	0.111943845	-0.138401598	0.300410212	0.469301795

[a] Numbering and the precise localization of CpG sites/units within CDH13 promoter region is provided in Figure S2. CpG sites 1-15 are located upstream from CpG island, sites 16-116 are within the CpG island and sites 18-26 are in the first exon.

[b] Genomic position on chromosome 16 (GRCh37/hg19).

[c] Effects and P-values are calculated using linear regression, including gender and experiment series as covariates to the model. Effect of age is determined as increase or decrease in CpG site methylation level in percentile scale (1-100) per annum (0= no methylation; 100= full methylation; positive beta-values= gain of methylation; negative beta-values= loss of methylation).

[d] Results were combined using the inverse-variance method under fixed-effects model.

SE, standard error; CI, confidence interval.

Table S6. SNP-s identified by promoter region resequencing

				HYPEST		CADCZ	
SNP[a]	Position on chr.16 GRCh37/hg19	Alleles[b]	Effect allele[c]	Effect allele frequency	HWE[d]	Effect allele frequency	HWE[d]
rs12443878	82,659,933	C/A	A	0.549	0.56	0.488	0.76
rs2239857	82,659,943	C/G	G	0.042	1	0.006	1
rs12444338	82,660,155	G/T	T	0.599	0.88	0.44	0.21
rs185121433	82,660,173	G/T	T	0.018	1	0.015	1
rs62040565	82,660,946	T/C	C	0.536	0.47	0.484	0.87
rs113460564	82,661,424	A/C	C	0.008	1	0.021	1
rs8060301	82,661,744	T/A	A	0.542	0.38	0.413	0.87
ss947846714	82,661,524	A/G	G	0.003	1	nd	
ss947846715	82,661,651	C/G	G	0.005	1	nd	
rs77068073	82,661,877	C/T	T	0.010	1	nd	
rs118163260	82,660,452	A/T	T	0.003	1	nd	
rs139512845	82,661,409	C/A	A	nd		0.006	1
rs111344280	82,659,615	G/A	A	nd		0.009	1
ss947846716	82,661,268	G/C	C	nd		0.006	1

[a]For novel variants ss numbers are given.

[b]Major/minor allele according to UCSC Genome Browser. For novel variants major/minor allele according to population where it was identified.

[c]Effect allele in genetic association analysis.

[d]HWE, *P*-value of the X^2-test for Hardy-Weinberg-Equilibrium

nd, not defined

Table S7. Genetic association between rs8060301, rs12444338, rs62040565, rs12443878 genotypes and DNA methylation in the EGCUT sample-set

SNP	SNP position[a]	CpG site[b]	CpG site position[a]	SNPType	Tested allele	BETA	SE	CI_2.5%	CI_97.5%	*P* -value[c]	FDR[d]
rs8060301	82,661,744	cg09415485	82,663,111	A/T	A	-0.6816	0.0574	-0.794	-0.56912	1.53E-32	1.89E-30
rs12444338	82,660,155	cg09415485	82,663,111	T/G	T	-0.572	0.0646	-0.6986	-0.44535	8.53E-19	5.29E-17
rs12443878	82,659,933	cg09415485	82,663,111	A/C	A	-0.4856	0.0689	-0.6205	-0.35059	1.78E-12	7.36E-11
rs62040565	82,660,946	cg09415485	82,663,111	C/T	C	-0.386	0.0727	-0.5285	-0.24354	1.10E-07	3.40E-06
rs8060301	82,661,744	cg19369556	82,661,725	A/T	A	-0.346	0.0739	-0.4909	-0.20117	2.84E-06	7.04E-05
rs12444338	82,660,155	cg19369556	82,661,725	T/G	T	-0.3102	0.0748	-0.4568	-0.16365	3.35E-05	0.0006915
rs12443878	82,659,933	cg19369556	82,661,725	A/C	A	-0.2604	0.076	-0.4094	-0.11137	0.000615	0.0108978
rs12443878	82,659,933	cg09044981	82,827,677	A/C	A	0.25661	0.0762	0.10734	0.40588	0.000753	0.0116788
rs8060301	82,661,744	cg09044981	82,827,677	A/T	A	0.20308	0.0764	0.05336	0.3528	0.007848	0.108131
rs12444338	82,660,155	cg09044981	82,827,677	T/G	T	0.19923	0.0771	0.04807	0.35039	0.009787	0.1213644
rs62040565	82,660,946	cg09044981	82,827,677	C/T	C	0.18489	0.0774	0.03314	0.33664	0.016939	0.1909515
rs12444338	82,660,155	cg09825093	82,660,505	T/G	T	0.17768	0.0776	0.02567	0.32969	0.021965	0.2269741
rs12443878	82,659,933	cg02069674	82,660,317	A/C	A	0.15141	0.0776	-0.0008	0.3036	0.051174	0.4250755
rs12443878	82,659,933	cg09825093	82,660,505	A/C	A	0.15083	0.0777	-0.0015	0.30321	0.05237	0.4250755
rs12443878	82,659,933	cg08271366	82,816,457	A/C	A	0.15111	0.0772	-0.0003	0.3025	0.050423	0.4250755
rs12443878	82,659,933	cg01880569	82,660,328	A/C	A	0.14536	0.0776	-0.0067	0.29741	0.060973	0.4250755
rs62040565	82,660,946	cg09825093	82,660,505	C/T	C	0.1463	0.0779	-0.0064	0.29895	0.060325	0.4250755
rs62040565	82,660,946	cg02069674	82,660,317	C/T	C	0.14565	0.078	-0.0071	0.29843	0.061705	0.4250755
rs62040565	82,660,946	cg19369556	82,661,725	C/T	C	-0.1401	0.0778	-0.2927	0.01248	0.07191	0.4693103
rs62040565	82,660,946	cg07301944	82,660,464	C/T	C	0.13534	0.0779	-0.0174	0.2881	0.082488	0.487074
rs8060301	82,661,744	cg07128660	82,474,489	A/T	A	-0.1348	0.0772	-0.2861	0.01644	0.080651	0.487074
rs8060301	82,661,744	cg02168291	82,671,520	A/T	A	-0.1293	0.078	-0.2822	0.02358	0.097366	0.5487897
rs12444338	82,660,155	cg08747377	82,660,670	T/G	T	-0.1257	0.0775	-0.2776	0.02625	0.104967	0.5659076
rs12444338	82,660,155	cg16777782	82,671,333	T/G	T	0.12447	0.078	-0.0283	0.27726	0.110342	0.5701002
rs12443878	82,659,933	cg16777782	82,671,333	A/C	A	0.11501	0.0778	-0.0375	0.26755	0.139483	0.5801549
rs12443878	82,659,933	cg08747377	82,660,670	A/C	A	-0.1155	0.0768	-0.2661	0.03508	0.132749	0.5801549
rs62040565	82,660,946	cg01880569	82,660,328	C/T	C	0.12202	0.078	-0.0309	0.27497	0.117913	0.5801549
rs62040565	82,660,946	cg00423443	82,660,376	C/T	C	0.11891	0.0782	-0.0343	0.27212	0.128235	0.5801549

rs62040565	82,660,946	cg02263260	82,658,496	C/T	C	0.11495	0.078	-0.0379	0.26776	0.14036	0.5801549
rs8060301	82,661,744	cg09825093	82,660,505	A/T	A	0.11991	0.0782	-0.0334	0.27317	0.125173	0.5801549
rs12444338	82,660,155	cg02495250	82,735,595	T/G	T	0.11177	0.0783	-0.0417	0.2652	0.153359	0.6134344
rs12443878	82,659,933	cg00806490	82,660,873	A/C	A	-0.1032	0.0779	-0.256	0.04951	0.185277	0.6433374
rs12443878	82,659,933	cg02495250	82,735,595	A/C	A	0.09682	0.0784	-0.0569	0.25054	0.217008	0.6433374
rs12443878	82,659,933	cg02263260	82,658,496	A/C	A	0.09474	0.0785	-0.059	0.24851	0.227203	0.6433374
rs12443878	82,659,933	cg07301944	82,660,464	A/C	A	0.08456	0.0783	-0.0689	0.23802	0.280163	0.6433374
rs12444338	82,660,155	cg02069674	82,660,317	T/G	T	0.10616	0.0783	-0.0473	0.25959	0.175095	0.6433374
rs12444338	82,660,155	cg00806490	82,660,873	T/G	T	-0.1072	0.0779	-0.2598	0.04546	0.168752	0.6433374
rs12444338	82,660,155	cg06341397	82,640,290	T/G	T	0.09201	0.0781	-0.0611	0.24513	0.238867	0.6433374
rs12444338	82,660,155	cg02168291	82,671,520	T/G	T	-0.0872	0.0784	-0.2409	0.06656	0.266394	0.6433374
rs12444338	82,660,155	cg08271366	82,816,457	T/G	T	0.08891	0.0778	-0.0636	0.24138	0.253113	0.6433374
rs12444338	82,660,155	cg07301944	82,660,464	T/G	T	0.08685	0.0781	-0.0662	0.23991	0.266076	0.6433374
rs12444338	82,660,155	cg01880569	82,660,328	T/G	T	0.08469	0.0784	-0.0689	0.23831	0.279899	0.6433374
rs62040565	82,660,946	cg08497530	82,660,434	C/T	C	-0.1009	0.0783	-0.2543	0.05248	0.197234	0.6433374
rs62040565	82,660,946	cg09109133	82,688,759	C/T	C	0.10047	0.0783	-0.053	0.25399	0.199555	0.6433374
rs62040565	82,660,946	cg08271366	82,816,457	C/T	C	0.09472	0.0779	-0.058	0.24741	0.224084	0.6433374
rs62040565	82,660,946	cg00806490	82,660,873	C/T	C	-0.0858	0.0785	-0.2396	0.06794	0.273942	0.6433374
rs8060301	82,661,744	cg16777782	82,671,333	A/T	A	0.10493	0.0784	-0.0487	0.25853	0.180565	0.6433374
rs8060301	82,661,744	cg08497530	82,660,434	A/T	A	-0.0983	0.0784	-0.2519	0.05532	0.209804	0.6433374
rs8060301	82,661,744	cg01090433	82,673,506	A/T	A	-0.0972	0.0784	-0.2509	0.05654	0.215321	0.6433374
rs8060301	82,661,744	cg16494747	82,660,206	A/T	A	-0.0934	0.0783	-0.2469	0.06	0.232638	0.6433374
rs8060301	82,661,744	cg02495250	82,735,595	A/T	A	0.09142	0.078	-0.0614	0.24425	0.241005	0.6433374
rs8060301	82,661,744	cg26709234	82,868,296	A/T	A	0.08958	0.0784	-0.0641	0.24327	0.253322	0.6433374
rs8060301	82,661,744	cg02263260	82,658,496	A/T	A	0.08891	0.0783	-0.0645	0.24233	0.255979	0.6433374
rs8060301	82,661,744	cg07301944	82,660,464	A/T	A	0.08793	0.0776	-0.0641	0.23996	0.256951	0.6433374
rs12443878	82,659,933	cg08497530	82,660,434	A/C	A	-0.0824	0.0785	-0.2363	0.07153	0.294069	0.6560091
rs12443878	82,659,933	cg02738205	82,741,062	A/C	A	-0.079	0.0784	-0.2328	0.07472	0.313699	0.6560091
rs12444338	82,660,155	cg02263260	82,658,496	T/G	T	0.07854	0.0786	-0.0754	0.2325	0.317424	0.6560091
rs62040565	82,660,946	cg08747377	82,660,670	C/T	C	-0.079	0.078	-0.2319	0.07383	0.310965	0.6560091
rs8060301	82,661,744	cg08271366	82,816,457	A/T	A	0.07945	0.0785	-0.0745	0.2334	0.311761	0.6560091

rs8060301	82,661,744	cg06341397	82,640,290	A/T	A	0.08035	0.0779	-0.0723	0.23303	0.302319	0.6560091
rs12443878	82,659,933	cg00423443	82,660,376	A/C	A	0.06879	0.0786	-0.0852	0.2228	0.381338	0.705648
rs12443878	82,659,933	cg16494747	82,660,206	A/C	A	0.06684	0.0785	-0.0871	0.22076	0.394673	0.705648
rs12443878	82,659,933	cg06341397	82,640,290	A/C	A	0.06569	0.0785	-0.0882	0.21957	0.40272	0.705648
rs12444338	82,660,155	cg05374412	82,660,727	T/G	T	-0.0697	0.0786	-0.2237	0.08428	0.374991	0.705648
rs12444338	82,660,155	cg09109133	82,688,759	T/G	T	0.06249	0.0783	-0.091	0.216	0.424914	0.705648
rs12444338	82,660,155	cg05949171	82,660,596	T/G	T	-0.0611	0.0784	-0.2147	0.09247	0.435495	0.705648
rs12444338	82,660,155	cg07128660	82,474,489	T/G	T	-0.0608	0.078	-0.2136	0.09204	0.435649	0.705648
rs62040565	82,660,946	cg16777782	82,671,333	C/T	C	0.07077	0.0786	-0.0833	0.22483	0.367916	0.705648
rs62040565	82,660,946	cg02168291	82,671,520	C/T	C	-0.0666	0.0786	-0.2207	0.08749	0.396817	0.705648
rs62040565	82,660,946	cg02755131	82,673,726	C/T	C	0.06579	0.0781	-0.0872	0.21878	0.399335	0.705648
rs62040565	82,660,946	cg07128660	82,474,489	C/T	C	0.06274	0.0786	-0.0914	0.21688	0.424973	0.705648
rs62040565	82,660,946	cg16494747	82,660,206	C/T	C	0.06268	0.0784	-0.091	0.2164	0.424143	0.705648
rs62040565	82,660,946	cg02495250	82,735,595	C/T	C	0.06179	0.0786	-0.0923	0.21594	0.432022	0.705648
rs62040565	82,660,946	cg05852523	82,659,960	C/T	C	0.06061	0.0782	-0.0926	0.21384	0.438185	0.705648
rs8060301	82,661,744	cg08977371	82,660,490	A/T	A	-0.0736	0.0785	-0.2274	0.08025	0.348493	0.705648
rs8060301	82,661,744	cg08747377	82,660,670	A/T	A	-0.0649	0.0782	-0.2182	0.08842	0.406778	0.705648
rs8060301	82,661,744	cg01301138	82,660,630	A/T	A	-0.0628	0.0786	-0.2168	0.09128	0.424461	0.705648
rs12444338	82,660,155	cg08497530	82,660,434	T/G	T	-0.0594	0.0786	-0.2134	0.09459	0.449592	0.7147357
rs12444338	82,660,155	cg00423443	82,660,376	T/G	T	0.05596	0.0787	-0.0982	0.21017	0.476904	0.7485589
rs62040565	82,660,946	cg02738205	82,741,062	C/T	C	-0.0527	0.0787	-0.207	0.10153	0.502957	0.7795827
rs12443878	82,659,933	cg05852523	82,659,960	A/C	A	0.04898	0.0785	-0.1049	0.2029	0.532822	0.807578
rs8060301	82,661,744	cg02069674	82,660,317	A/T	A	0.04892	0.0787	-0.1054	0.2032	0.534313	0.807578
rs8060301	82,661,744	cg19854301	82,671,450	A/T	A	-0.0482	0.0787	-0.2025	0.10611	0.540556	0.807578
rs12443878	82,659,933	cg09109133	82,688,759	A/C	A	0.04583	0.0786	-0.1082	0.19986	0.559811	0.8263872
rs12443878	82,659,933	cg01090433	82,673,506	A/C	A	-0.0448	0.0787	-0.1991	0.10942	0.568909	0.8299378
rs12444338	82,660,155	cg08977371	82,660,490	T/G	T	-0.0433	0.0786	-0.1973	0.11072	0.58173	0.8387735
rs12443878	82,659,933	cg02168291	82,671,520	A/C	A	-0.0403	0.0787	-0.1947	0.11399	0.608369	0.8517527
rs12443878	82,659,933	cg05374412	82,660,727	A/C	A	-0.04	0.0787	-0.1943	0.11431	0.611339	0.8517527
rs62040565	82,660,946	cg05949171	82,660,596	C/T	C	0.04075	0.0782	-0.1125	0.19399	0.602187	0.8517527
rs12443878	82,659,933	cg01301138	82,660,630	A/C	A	-0.0388	0.0786	-0.1929	0.1153	0.62154	0.8563441

rs12443878	82,659,933	cg19854301	82,671,450	A/C	A	0.03374	0.0788	-0.1206	0.18811	0.668427	0.8640861
rs12444338	82,660,155	cg05852523	82,659,960	T/G	T	-0.0355	0.0782	-0.1888	0.11774	0.64959	0.8640861
rs12444338	82,660,155	cg01090433	82,673,506	T/G	T	-0.034	0.0787	-0.1883	0.12031	0.665723	0.8640861
rs12444338	82,660,155	cg01301138	82,660,630	T/G	T	-0.0336	0.0787	-0.1879	0.12059	0.668982	0.8640861
rs12444338	82,660,155	cg26709234	82,868,296	T/G	T	0.03293	0.0786	-0.1211	0.18694	0.67514	0.8640861
rs8060301	82,661,744	cg04029645	82,665,755	A/T	A	-0.0361	0.0787	-0.1905	0.11821	0.646332	0.8640861
rs8060301	82,661,744	cg02755131	82,673,726	A/T	A	-0.0329	0.0787	-0.1871	0.12131	0.675938	0.8640861
rs12443878	82,659,933	cg07128660	82,474,489	A/C	A	-0.0311	0.0785	-0.1849	0.12277	0.69228	0.8759467
rs8060301	82,661,744	cg00806490	82,660,873	A/T	A	-0.03	0.078	-0.1828	0.12286	0.700736	0.87769
rs12444338	82,660,155	cg02738205	82,741,062	T/G	T	0.02553	0.0787	-0.1287	0.1798	0.745664	0.8985533
rs12444338	82,660,155	cg02755131	82,673,726	T/G	T	0.02538	0.0785	-0.1284	0.17922	0.746379	0.8985533
rs62040565	82,660,946	cg19854301	82,671,450	C/T	C	0.02665	0.0787	-0.1277	0.18099	0.73507	0.8985533
rs62040565	82,660,946	cg01090433	82,673,506	C/T	C	-0.0258	0.0788	-0.1802	0.12859	0.743136	0.8985533
rs12443878	82,659,933	cg08977371	82,660,490	A/C	A	-0.021	0.0785	-0.1748	0.13279	0.788858	0.9154936
rs12444338	82,660,155	cg19854301	82,671,450	T/G	T	-0.0215	0.0788	-0.1759	0.13294	0.785316	0.9154936
rs62040565	82,660,946	cg05374412	82,660,727	C/T	C	-0.0227	0.0788	-0.1771	0.13166	0.772948	0.9154936
rs62040565	82,660,946	cg08977371	82,660,490	C/T	C	0.02096	0.0787	-0.1333	0.17521	0.789982	0.9154936
rs62040565	82,660,946	cg26709234	82,868,296	C/T	C	0.01868	0.0787	-0.1356	0.17298	0.812431	0.9327914
rs12443878	82,659,933	cg04029645	82,665,755	A/C	A	0.01554	0.0784	-0.1381	0.16916	0.842837	0.9487525
rs12443878	82,659,933	cg26709234	82,868,296	A/C	A	0.01164	0.0784	-0.142	0.16523	0.881944	0.9487525
rs12444338	82,660,155	cg16494747	82,660,206	T/G	T	0.0103	0.0788	-0.1441	0.16472	0.896025	0.9487525
rs62040565	82,660,946	cg01301138	82,660,630	C/T	C	-0.012	0.0785	-0.1659	0.1419	0.878385	0.9487525
rs62040565	82,660,946	cg04029645	82,665,755	C/T	C	-0.0118	0.0783	-0.1654	0.14174	0.880126	0.9487525
rs8060301	82,661,744	cg09109133	82,688,759	A/T	A	0.01639	0.0787	-0.1378	0.17059	0.834936	0.9487525
rs8060301	82,661,744	cg00423443	82,660,376	A/T	A	0.01016	0.0787	-0.1441	0.16443	0.897315	0.9487525
rs8060301	82,661,744	cg01880569	82,660,328	A/T	A	0.0094	0.0786	-0.1446	0.16343	0.904841	0.9487525
rs8060301	82,661,744	cg02738205	82,741,062	A/T	A	-0.0095	0.0786	-0.1636	0.14465	0.903861	0.9487525
rs8060301	82,661,744	cg05949171	82,660,596	A/T	A	-0.0092	0.0787	-0.1635	0.1451	0.907003	0.9487525
rs8060301	82,661,744	cg05852523	82,659,960	A/T	A	-0.0088	0.0783	-0.1623	0.1447	0.910496	0.9487525
rs12443878	82,659,933	cg05949171	82,660,596	A/C	A	-0.0056	0.0782	-0.159	0.14772	0.942538	0.9739559
rs12444338	82,660,155	cg04029645	82,665,755	T/G	T	0.00437	0.0787	-0.1498	0.15853	0.955658	0.9793522

rs12443878	82,659,933	cg02755131	82,673,726	A/C	A	0.00216	0.0782	-0.1511	0.15541	0.977934	0.9819966
rs62040565	82,660,946	cg06341397	82,640,290	C/T	C	0.00265	0.0786	-0.1514	0.15673	0.973085	0.9819966
rs8060301	82,661,744	cg05374412	82,660,727	A/T	A	-0.0018	0.0788	-0.1561	0.15259	0.981997	0.9819966

[a] Genomic position on chromosome 16 (GRCh37/hg19)

[b] CpG-site code on Infinium HumanMethylation450 BeadChip

[c] Effects (tested allele effect on DNA methylation on transformed scale (Material and Methods)) and *P* -values are calculated using linear regression.

[d] The distribution of observed *P* -values was used to calculate the Benjamin-Hochberg false-discovery rate (FDR).

SE, standard error; CI, confidence interval.

Table S8. Results of HYPEST-CADCZ meta-analysis for association tests between the DNA methylation level at targeted CDH13 promoter CpG sites/units and cardio-metabolic traits

			HYPEST				
CpG site/unit[a]	Position at chr.16[b]	Trait	Beta	SE	CI_2.5%	CI_97.5%	*P* -value[c]
CpG_1	82,660,317	SBP	-0.409413	0.290489	-0.978761	0.159935	0.162127
CpG_1	82,660,317	DBP	-0.047645	0.184833	-0.40991	0.314621	0.797165
CpG_1	82,660,317	Total cholesterol	0.001368	0.014208	-0.02648	0.029215	0.923429
CpG_1	82,660,317	HDL	0.003945	0.004493	-0.00486	0.01275	0.381319
CpG_1	82,660,317	LDL	-0.00378	0.01081	-0.024967	0.017406	0.727041
CpG_1	82,660,317	Triglycerides	0.019272	0.013937	-0.008044	0.046588	0.168831
CpG_2	82,660,328	SBP	-0.666632	0.277628	-1.210773	-0.122491	0.018424
CpG_2	82,660,328	DBP	-0.334633	0.177344	-0.682222	0.012955	0.062432
CpG_2	82,660,328	Total cholesterol	0.008025	0.014203	-0.019812	0.035862	0.572915
CpG_2	82,660,328	HDL	0.000779	0.00451	-0.00806	0.009617	0.863167
CpG_2	82,660,328	LDL	-0.003234	0.010801	-0.024402	0.017935	0.765056
CpG_2	82,660,328	Triglycerides	0.001183	0.014122	-0.026496	0.028863	0.933333
CpG_3	82,660,376	SBP	-0.01577	0.395999	-0.791914	0.760374	0.968322
CpG_3	82,660,376	DBP	0.152466	0.249646	-0.336831	0.641763	0.542919
CpG_3	82,660,376	Total cholesterol	-0.005109	0.018581	-0.041526	0.031308	0.783747
CpG_3	82,660,376	HDL	-0.003115	0.005889	-0.014657	0.008427	0.59763
CpG_3	82,660,376	LDL	-0.013207	0.014087	-0.040817	0.014403	0.350028
CpG_3	82,660,376	Triglycerides	-0.002068	0.018301	-0.037937	0.033801	0.910189
CpG_9	82,660,464	SBP	-0.256034	0.516289	-1.267943	0.755874	0.621152
CpG_9	82,660,464	DBP	0.027498	0.325522	-0.610514	0.66551	0.932865
CpG_9	82,660,464	Total cholesterol	0.062576	0.025318	0.012953	0.112198	0.014578
CpG_9	82,660,464	HDL	0.007292	0.00816	-0.0087	0.023285	0.372932
CpG_9	82,660,464	LDL	0.04993	0.019284	0.012133	0.087727	0.010575
CpG_9	82,660,464	Triglycerides	0.026084	0.025361	-0.023623	0.075791	0.305392
CpG_11	82,660,490	SBP	0.064863	0.248467	-0.422124	0.55185	0.794699
CpG_11	82,660,490	DBP	0.181621	0.155089	-0.122349	0.48559	0.244921
CpG_11	82,660,490	Total cholesterol	0.019152	0.013452	-0.007215	0.045518	0.156769
CpG_11	82,660,490	HDL	0.002721	0.004319	-0.005745	0.011187	0.529758

CpG_11	82,660,490	LDL	0.003605	0.010367	-0.016715	0.023924	0.728586
CpG_11	82,660,490	Triglycerides	-0.015005	0.013549	-0.041561	0.011551	0.270002
CpG_12	82,660,505	SBP	-0.314046	0.774733	-1.832496	1.204404	0.686164
CpG_12	82,660,505	DBP	-0.097326	0.488166	-1.054114	0.859462	0.842418
CpG_12	82,660,505	Total cholesterol	0.020246	0.03572	-0.049764	0.090256	0.571702
CpG_12	82,660,505	HDL	-0.010215	0.011368	-0.032496	0.012065	0.370299
CpG_12	82,660,505	LDL	0.03586	0.027185	-0.01742	0.089141	0.189132
CpG_12	82,660,505	Triglycerides	-0.032437	0.035193	-0.101414	0.036539	0.358171
CpG_13	82,660,554	SBP	0.78653	1.487392	-2.128705	3.701765	0.598233
CpG_13	82,660,554	DBP	0.455762	0.936798	-1.380329	2.291853	0.627775
CpG_13	82,660,554	Total cholesterol	0.009189	0.075765	-0.139307	0.157686	0.903628
CpG_13	82,660,554	HDL	0.031434	0.023859	-0.015328	0.078196	0.189688
CpG_13	82,660,554	LDL	-0.01827	0.057803	-0.131561	0.095021	0.752388
CpG_13	82,660,554	Triglycerides	0.007631	0.074641	-0.138662	0.153924	0.918706
CpG_15	82,660,630	SBP	-0.648617	1.281787	-3.160874	1.86364	0.614049
CpG_15	82,660,630	DBP	-1.229007	0.815737	-2.827821	0.369808	0.135334
CpG_15	82,660,630	Total cholesterol	0.102997	0.077033	-0.047985	0.253979	0.183272
CpG_15	82,660,630	HDL	0.017195	0.026124	-0.034006	0.068397	0.511418
CpG_15	82,660,630	LDL	0.093191	0.058487	-0.021441	0.207824	0.113226
CpG_15	82,660,630	Triglycerides	-0.077664	0.077733	-0.230018	0.07469	0.319404
CpG_16-17	82,660,652-82,660,654	SBP	0.676379	1.414151	-2.095305	3.448064	0.633576
CpG_16-17	82,660,652-82,660,654	DBP	0.597217	0.908743	-1.183887	2.37832	0.5127
CpG_16-17	82,660,652-82,660,654	Total cholesterol	0.036757	0.066316	-0.093219	0.166733	0.580229
CpG_16-17	82,660,652-82,660,654	HDL	-0.002169	0.022409	-0.04609	0.041752	0.923008
CpG_16-17	82,660,652-82,660,654	LDL	-0.026591	0.050482	-0.125534	0.072352	0.599163
CpG_16-17	82,660,652-82,660,654	Triglycerides	0.065576	0.066706	-0.065165	0.196316	0.327215
CpG_22	82,660,690	SBP	-0.053947	0.912504	-1.842422	1.734528	0.952985
CpG_22	82,660,690	DBP	-0.374057	0.585741	-1.522089	0.773974	0.524667
CpG_22	82,660,690	Total cholesterol	0.054329	0.051621	-0.046847	0.155505	0.294318
CpG_22	82,660,690	HDL	0.025428	0.017365	-0.008607	0.059464	0.145249
CpG_22	82,660,690	LDL	0.038156	0.039314	-0.038898	0.11521	0.33337
CpG_22	82,660,690	Triglycerides	-0.032681	0.051927	-0.134457	0.069095	0.530105
CpG_23	82,660,705	SBP	-2.138152	1.752134	-5.572272	1.295968	0.225465

CpG_23	82,660,705	DBP	-1.78984	1.120831	-3.986628	0.406949	0.11372
CpG_23	82,660,705	Total cholesterol	0.060096	0.101062	-0.137982	0.258173	0.552997
CpG_23	82,660,705	HDL	0.00803	0.03415	-0.058904	0.074963	0.814435
CpG_23	82,660,705	LDL	0.009831	0.077013	-0.141111	0.160773	0.898598
CpG_23	82,660,705	Triglycerides	-0.012796	0.101364	-0.211466	0.185873	0.899715
CpG_27-29	82,660,761-82,660,767	SBP	-0.167132	1.066495	-2.257424	1.92316	0.875823
CpG_27-29	82,660,761-82,660,767	DBP	-0.275889	0.690554	-1.629351	1.077572	0.690458
CpG_27-29	82,660,761-82,660,767	Total cholesterol	-0.032198	0.060339	-0.15046	0.086064	0.594435
CpG_27-29	82,660,761-82,660,767	HDL	-0.059473	0.019775	-0.098231	-0.020715	0.003116
CpG_27-29	82,660,761-82,660,767	LDL	0.032156	0.045887	-0.05778	0.122092	0.484589
CpG_27-29	82,660,761-82,660,767	Triglycerides	0.045085	0.060219	-0.072942	0.163113	0.4553
CpG_30-31	82,660,774-82,660,779	SBP	1.553888	4.175282	-6.629514	9.73729	0.710627
CpG_30-31	82,660,774-82,660,779	DBP	1.889831	2.680808	-3.364457	7.144118	0.482623
CpG_30-31	82,660,774-82,660,779	Total cholesterol	-0.124797	0.241504	-0.598137	0.348543	0.606109
CpG_30-31	82,660,774-82,660,779	HDL	-0.007604	0.081597	-0.167532	0.152323	0.925875
CpG_30-31	82,660,774-82,660,779	LDL	-0.221675	0.18308	-0.580505	0.137156	0.227913
CpG_30-31	82,660,774-82,660,779	Triglycerides	0.692354	0.235449	0.230883	1.153826	0.003814
CpG_32	82,660,791	SBP	-1.491522	0.912342	-3.27968	0.296635	0.106064
CpG_32	82,660,791	DBP	-0.343956	0.606234	-1.532153	0.844241	0.572075
CpG_32	82,660,791	Total cholesterol	-0.022061	0.060895	-0.141412	0.097291	0.71773
CpG_32	82,660,791	HDL	0.037846	0.017938	0.002688	0.073005	0.036779
CpG_32	82,660,791	LDL	-0.04527	0.045294	-0.134043	0.043504	0.319411
CpG_32	82,660,791	Triglycerides	-0.038511	0.06032	-0.156735	0.079713	0.524313
CpG_36	82,660,826	SBP	0.003047	0.703881	-1.376534	1.382629	0.996556
CpG_36	82,660,826	DBP	-0.080303	0.454064	-0.970253	0.809647	0.860065
CpG_36	82,660,826	Total cholesterol	0.01809	0.046517	-0.073081	0.109261	0.697976
CpG_36	82,660,826	HDL	0.00359	0.01379	-0.023438	0.030618	0.795015
CpG_36	82,660,826	LDL	0.035555	0.03492	-0.032887	0.103997	0.310428
CpG_36	82,660,826	Triglycerides	0.001542	0.045913	-0.088446	0.09153	0.97326
CpG_37	82,660,834	SBP	-3.152186	3.557163	-10.1241	3.819725	0.377846
CpG_37	82,660,834	DBP	-1.873476	2.289803	-6.361407	2.614455	0.415367
CpG_37	82,660,834	Total cholesterol	0.067677	0.178099	-0.281391	0.416745	0.704498
CpG_37	82,660,834	HDL	-0.073289	0.059847	-0.190586	0.044009	0.222682

CpG_37	82,660,834	LDL	-0.069966	0.135506	-0.335553	0.195621	0.606398
CpG_37	82,660,834	Triglycerides	0.129214	0.179199	-0.222009	0.480438	0.472029
CpG_38	82,660,843	SBP	-0.153907	0.598221	-1.326399	1.018585	0.797581
CpG_38	82,660,843	DBP	0.06865	0.385968	-0.687833	0.825132	0.859248
CpG_38	82,660,843	Total cholesterol	-0.014635	0.034271	-0.081806	0.052536	0.670007
CpG_38	82,660,843	HDL	0.011951	0.011708	-0.010997	0.034899	0.309123
CpG_38	82,660,843	LDL	-0.013536	0.025906	-0.064311	0.037238	0.602127
CpG_38	82,660,843	Triglycerides	-0.046883	0.034495	-0.114491	0.020726	0.176308
CpG_39-42	82,660,847-82,660,855	SBP	-1.468101	1.099573	-3.623224	0.687023	0.185156
CpG_39-42	82,660,847-82,660,855	DBP	-0.720146	0.710728	-2.113148	0.672855	0.313627
CpG_39-42	82,660,847-82,660,855	Total cholesterol	0.055692	0.059624	-0.061169	0.172552	0.35184
CpG_39-42	82,660,847-82,660,855	HDL	0.026395	0.01996	-0.012726	0.065515	0.188139
CpG_39-42	82,660,847-82,660,855	LDL	0.008662	0.045473	-0.080463	0.097788	0.849191
CpG_39-42	82,660,847-82,660,855	Triglycerides	-0.005455	0.059884	-0.122826	0.111916	0.927548
CpG_43	82,660,866	SBP	0.249827	0.566202	-0.859908	1.359562	0.660079
CpG_43	82,660,866	DBP	-0.210245	0.363971	-0.923616	0.503125	0.564917
CpG_43	82,660,866	Total cholesterol	-0.011318	0.021347	-0.053157	0.030522	0.596794
CpG_43	82,660,866	HDL	0.001736	0.007212	-0.012398	0.015871	0.810061
CpG_43	82,660,866	LDL	-0.007308	0.016253	-0.039163	0.024547	0.653621
CpG_43	82,660,866	Triglycerides	-0.017513	0.021339	-0.059338	0.024311	0.413158
CpG_45	82,660,890	SBP	0.702245	0.44767	-0.175172	1.579662	0.120159
CpG_45	82,660,890	DBP	0.076151	0.29171	-0.495591	0.647893	0.794637
CpG_45	82,660,890	Total cholesterol	-0.018929	0.024327	-0.06661	0.028752	0.437766
CpG_45	82,660,890	HDL	0.004832	0.00822	-0.011278	0.020942	0.557556
CpG_45	82,660,890	LDL	0.013062	0.018524	-0.023244	0.049368	0.481845
CpG_45	82,660,890	Triglycerides	-0.019777	0.024679	-0.068146	0.028592	0.424227
CpG_52	82,661,050	SBP	0.736498	0.586593	-0.413203	1.886199	0.212801
CpG_52	82,661,050	DBP	0.682355	0.424364	-0.149383	1.514093	0.111644
CpG_52	82,661,050	Total cholesterol	0.029957	0.030275	-0.029381	0.089296	0.32418
CpG_52	82,661,050	HDL	0.008423	0.011661	-0.014433	0.031278	0.471365
CpG_52	82,661,050	LDL	0.022312	0.026355	-0.029342	0.073966	0.398708
CpG_52	82,661,050	Triglycerides	-0.007839	0.024519	-0.055896	0.040217	0.749672
CpG_53	82,661,087	SBP	0.817954	0.718592	-0.590461	2.226369	0.260656

CpG_53	82,661,087	DBP	0.588908	0.508921	-0.408559	1.586374	0.252929
CpG_53	82,661,087	Total cholesterol	0.003506	0.029986	-0.055267	0.062278	0.907258
CpG_53	82,661,087	HDL	-0.022098	0.011408	-0.044456	0.000261	0.056609
CpG_53	82,661,087	LDL	0.00406	0.026176	-0.047243	0.055364	0.877155
CpG_53	82,661,087	Triglycerides	0.043707	0.022802	-0.000984	0.088399	0.059179
CpG_54	82,661,095	SBP	0.312273	0.480914	-0.6303	1.254847	0.517916
CpG_54	82,661,095	DBP	-0.183379	0.350291	-0.869936	0.503179	0.602019
CpG_54	82,661,095	Total cholesterol	0.003811	0.025134	-0.045452	0.053073	0.879724
CpG_54	82,661,095	HDL	-0.009866	0.009675	-0.028829	0.009098	0.309722
CpG_54	82,661,095	LDL	-0.001808	0.021803	-0.04454	0.040925	0.93405
CpG_54	82,661,095	Triglycerides	0.008401	0.020178	-0.031148	0.047949	0.677846
CpG_57-58	82,661,139-82,661,141	SBP	0.093404	0.284919	-0.465028	0.651836	0.74389
CpG_57-58	82,661,139-82,661,141	DBP	0.241416	0.204063	-0.15854	0.641373	0.240251
CpG_57-58	82,661,139-82,661,141	Total cholesterol	0.007132	0.015538	-0.023321	0.037585	0.646993
CpG_57-58	82,661,139-82,661,141	HDL	-0.001162	0.005581	-0.0121	0.009775	0.835328
CpG_57-58	82,661,139-82,661,141	LDL	0.00581	0.013514	-0.020677	0.032297	0.667961
CpG_57-58	82,661,139-82,661,141	Triglycerides	0.004972	0.012156	-0.018854	0.028798	0.683243
CpG_62-64	82,661,175-82,661,188	SBP	-1.048639	0.51651	-2.060981	-0.036297	0.045927
CpG_62-64	82,661,175-82,661,188	DBP	-0.953686	0.360908	-1.661053	-0.246319	0.010039
CpG_62-64	82,661,175-82,661,188	Total cholesterol	-0.024401	0.024019	-0.071477	0.022676	0.31173
CpG_62-64	82,661,175-82,661,188	HDL	0.006209	0.008589	-0.010625	0.023043	0.471156
CpG_62-64	82,661,175-82,661,188	LDL	-0.032607	0.020955	-0.073677	0.008463	0.122324
CpG_62-64	82,661,175-82,661,188	Triglycerides	0.002153	0.018935	-0.034959	0.039264	0.909681
CpG_66	82,661,202	SBP	-0.473685	0.469462	-1.393814	0.446444	0.31591
CpG_66	82,661,202	DBP	-0.353677	0.341537	-1.023077	0.315723	0.303422
CpG_66	82,661,202	Total cholesterol	0.003469	0.02598	-0.047451	0.054389	0.893978
CpG_66	82,661,202	HDL	-0.005483	0.009959	-0.025003	0.014037	0.582857
CpG_66	82,661,202	LDL	0.003757	0.022612	-0.040562	0.048075	0.868302
CpG_66	82,661,202	Triglycerides	0.012794	0.020924	-0.028216	0.053805	0.541929
CpG_67	82,661,217	SBP	-0.851558	1.191733	-3.187311	1.484195	0.476914
CpG_67	82,661,217	DBP	-0.897245	0.868018	-2.598528	0.804039	0.304329
CpG_67	82,661,217	Total cholesterol	-0.105661	0.063595	-0.230306	0.018983	0.098941
CpG_67	82,661,217	HDL	0.004387	0.024435	-0.043504	0.052278	0.857785

CpG_67	82,661,217	LDL	-0.121451	0.054856	-0.228966	-0.013935	0.028507
CpG_67	82,661,217	Triglycerides	-0.03141	0.051746	-0.132829	0.07001	0.544876
CpG_69-70	82,661,244-82,661,248	SBP	-0.960038	0.870306	-2.665806	0.745731	0.273331
CpG_69-70	82,661,244-82,661,248	DBP	-0.507182	0.610307	-1.703362	0.688997	0.408462
CpG_69-70	82,661,244-82,661,248	Total cholesterol	-0.088813	0.0454	-0.177795	0.000168	0.052579
CpG_69-70	82,661,244-82,661,248	HDL	-0.00707	0.015773	-0.037985	0.023844	0.654707
CpG_69-70	82,661,244-82,661,248	LDL	-0.092348	0.038824	-0.168442	-0.016254	0.018832
CpG_69-70	82,661,244-82,661,248	Triglycerides	-0.023973	0.036578	-0.095665	0.04772	0.51339
CpG_71-72	82,661,267-82,661,275	SBP	-0.207865	0.844873	-1.863787	1.448056	0.806288
CpG_71-72	82,661,267-82,661,275	DBP	-0.128396	0.6132	-1.330245	1.073453	0.834678
CpG_71-72	82,661,267-82,661,275	Total cholesterol	-0.076275	0.04168	-0.157966	0.005416	0.069521
CpG_71-72	82,661,267-82,661,275	HDL	0.021892	0.016034	-0.009534	0.053317	0.174486
CpG_71-72	82,661,267-82,661,275	LDL	-0.08362	0.035925	-0.154031	-0.013209	0.021463
CpG_71-72	82,661,267-82,661,275	Triglycerides	-0.034363	0.033943	-0.100891	0.032165	0.313241
CpG_73-74	82,661,285-82,661,290	SBP	-4.175494	1.622271	-7.355087	-0.995901	0.011879
CpG_73-74	82,661,285-82,661,290	DBP	-2.792638	1.181531	-5.108397	-0.476879	0.020495
CpG_73-74	82,661,285-82,661,290	Total cholesterol	-0.032156	0.072105	-0.173479	0.109167	0.656354
CpG_73-74	82,661,285-82,661,290	HDL	0.02323	0.024941	-0.025654	0.072114	0.353346
CpG_73-74	82,661,285-82,661,290	LDL	-0.040242	0.061604	-0.160984	0.0805	0.514741
CpG_73-74	82,661,285-82,661,290	Triglycerides	0.003369	0.057619	-0.109562	0.116301	0.953457
CpG_75-76	82,661,313-82,661,326	SBP	-1.051914	1.020897	-3.052836	0.949008	0.306142
CpG_75-76	82,661,313-82,661,326	DBP	-0.219325	0.744263	-1.678053	1.239403	0.769047
CpG_75-76	82,661,313-82,661,326	Total cholesterol	-0.032326	0.04564	-0.121779	0.057128	0.480086
CpG_75-76	82,661,313-82,661,326	HDL	0.028735	0.016055	-0.002732	0.060202	0.075889
CpG_75-76	82,661,313-82,661,326	LDL	-0.067953	0.039421	-0.145217	0.009311	0.0872
CpG_75-76	82,661,313-82,661,326	Triglycerides	-0.002108	0.036271	-0.073197	0.068981	0.953747
CpG_77-81	82,661,336-82,661,349	SBP	-0.540071	0.741984	-1.994334	0.914191	0.468816
CpG_77-81	82,661,336-82,661,349	DBP	-0.388717	0.52739	-1.422383	0.644949	0.463244
CpG_77-81	82,661,336-82,661,349	Total cholesterol	-0.093802	0.045073	-0.182144	-0.00546	0.039343
CpG_77-81	82,661,336-82,661,349	HDL	-0.01472	0.01753	-0.049079	0.019638	0.402577
CpG_77-81	82,661,336-82,661,349	LDL	-0.073995	0.039244	-0.150912	0.002922	0.061542
CpG_77-81	82,661,336-82,661,349	Triglycerides	-0.028113	0.036942	-0.100518	0.044292	0.448012
CpG_83	82,661,360	SBP	-0.060796	0.340415	-0.727997	0.606406	0.858709

CpG_83	82,661,360	DBP	-0.316178	0.243201	-0.792842	0.160487	0.19731
CpG_83	82,661,360	Total cholesterol	-0.013828	0.013239	-0.039776	0.01212	0.298156
CpG_83	82,661,360	HDL	0.006107	0.004588	-0.002886	0.0151	0.185468
CpG_83	82,661,360	LDL	-0.015899	0.011285	-0.038018	0.00622	0.161241
CpG_83	82,661,360	Triglycerides	-0.019406	0.010527	-0.040039	0.001227	0.067523
CpG_84-85	82,661,384-82,661,390	SBP	-0.017177	0.289267	-0.58413	0.549777	0.952799
CpG_84-85	82,661,384-82,661,390	DBP	0.111195	0.208933	-0.298306	0.520696	0.596078
CpG_84-85	82,661,384-82,661,390	Total cholesterol	-0.002247	0.015216	-0.032069	0.027576	0.882855
CpG_84-85	82,661,384-82,661,390	HDL	0.000691	0.005835	-0.010745	0.012126	0.905919
CpG_84-85	82,661,384-82,661,390	LDL	0.002236	0.013204	-0.023643	0.028116	0.865773
CpG_84-85	82,661,384-82,661,390	Triglycerides	-0.001697	0.012147	-0.025504	0.022109	0.889077
CpG_86	82,661,399	SBP	-0.062355	0.125583	-0.308493	0.183782	0.62085
CpG_86	82,661,399	DBP	-0.056515	0.091753	-0.236348	0.123319	0.539638
CpG_86	82,661,399	Total cholesterol	-0.003928	0.007677	-0.018975	0.011119	0.609756
CpG_86	82,661,399	HDL	0.000879	0.002953	-0.004909	0.006667	0.76643
CpG_86	82,661,399	LDL	-0.004078	0.006642	-0.017096	0.00894	0.540282
CpG_86	82,661,399	Triglycerides	-0.006732	0.006161	-0.018808	0.005343	0.276501
CpG_87-88	82,661,404-82,661,413	SBP	-0.185451	0.350062	-0.871559	0.500657	0.597722
CpG_87-88	82,661,404-82,661,413	DBP	0.059474	0.254515	-0.439366	0.558315	0.815825
CpG_87-88	82,661,404-82,661,413	Total cholesterol	0.026463	0.019212	-0.011191	0.064118	0.170704
CpG_87-88	82,661,404-82,661,413	HDL	0.002005	0.007471	-0.012637	0.016648	0.788788
CpG_87-88	82,661,404-82,661,413	LDL	0.01892	0.01664	-0.013695	0.051534	0.257605
CpG_87-88	82,661,404-82,661,413	Triglycerides	0.003338	0.015643	-0.027322	0.033998	0.831371
CpG_89	82,661,421	SBP	-0.704706	0.723908	-2.123541	0.714128	0.333926
CpG_89	82,661,421	DBP	-0.488188	0.504813	-1.477603	0.501227	0.337095
CpG_89	82,661,421	Total cholesterol	-0.034302	0.038402	-0.109569	0.040965	0.373736
CpG_89	82,661,421	HDL	-0.015403	0.014461	-0.043746	0.012941	0.289236
CpG_89	82,661,421	LDL	-0.011605	0.033955	-0.078156	0.054946	0.733191
CpG_89	82,661,421	Triglycerides	-0.030432	0.030181	-0.089586	0.028722	0.315576
CpG_90	82,661,438	SBP	-0.2733	0.572219	-1.394829	0.848229	0.63418
CpG_90	82,661,438	DBP	-0.382342	0.414882	-1.195496	0.430812	0.359425
CpG_90	82,661,438	Total cholesterol	0.008889	0.027994	-0.045979	0.063756	0.751338
CpG_90	82,661,438	HDL	-0.001597	0.010768	-0.022702	0.019507	0.882284

CpG_90	82,661,438	LDL	0.000657	0.024355	-0.047077	0.048391	0.978523
CpG_90	82,661,438	Triglycerides	0.000508	0.022657	-0.043899	0.044915	0.982139
CpG_91	82,661,469	SBP	0.094573	0.323877	-0.540215	0.729361	0.771627
CpG_91	82,661,469	DBP	0.11018	0.240197	-0.360597	0.580957	0.648652
CpG_91	82,661,469	Total cholesterol	-0.008272	0.020724	-0.048889	0.032346	0.690841
CpG_91	82,661,469	HDL	-0.001634	0.006507	-0.014387	0.01112	0.802375
CpG_91	82,661,469	LDL	0.002395	0.016105	-0.029169	0.03396	0.882136
CpG_91	82,661,469	Triglycerides	-0.005855	0.012997	-0.031329	0.019618	0.653556
CpG_92	82,661,480	SBP	0.282549	2.263617	-4.15406	4.719157	0.901221
CpG_92	82,661,480	DBP	1.618252	1.663988	-1.643104	4.879608	0.335994
CpG_92	82,661,480	Total cholesterol	-0.017719	0.100845	-0.215371	0.179933	0.860962
CpG_92	82,661,480	HDL	0.058378	0.030979	-0.00234	0.119096	0.063096
CpG_92	82,661,480	LDL	-0.005757	0.078314	-0.159249	0.147735	0.941583
CpG_92	82,661,480	Triglycerides	-0.108212	0.062085	-0.229897	0.013473	0.085184
CpG_104	82,661,633	SBP	-0.249434	0.453464	-1.138207	0.639338	0.585057
CpG_104	82,661,633	DBP	0.287621	0.335908	-0.370746	0.945988	0.396499
CpG_104	82,661,633	Total cholesterol	-0.025405	0.025266	-0.074926	0.024116	0.317772
CpG_104	82,661,633	HDL	0.005111	0.007873	-0.010319	0.020541	0.518106
CpG_104	82,661,633	LDL	-0.015264	0.019523	-0.053528	0.022999	0.436656
CpG_104	82,661,633	Triglycerides	0.004567	0.015806	-0.026412	0.035546	0.773423
CpG_106	82,661,670	SBP	0.712323	0.587745	-0.439636	1.864282	0.231854
CpG_106	82,661,670	DBP	0.06633	0.44345	-0.802815	0.935475	0.881766
CpG_106	82,661,670	Total cholesterol	0.008265	0.032297	-0.055036	0.071565	0.798679
CpG_106	82,661,670	HDL	-0.009068	0.010089	-0.028842	0.010705	0.371388
CpG_106	82,661,670	LDL	0.011381	0.025055	-0.037726	0.060488	0.650878
CpG_106	82,661,670	Triglycerides	0.044855	0.019644	0.006353	0.083356	0.02506
CpG_107	82,661,696	SBP	-0.848701	1.000678	-2.809993	1.112591	0.400958
CpG_107	82,661,696	DBP	-0.332682	0.747135	-1.79704	1.131675	0.658303
CpG_107	82,661,696	Total cholesterol	0.059201	0.054635	-0.047881	0.166283	0.281851
CpG_107	82,661,696	HDL	0.017141	0.01765	-0.017452	0.051734	0.334424
CpG_107	82,661,696	LDL	0.022056	0.042587	-0.061414	0.105525	0.605983
CpG_107	82,661,696	Triglycerides	0.036652	0.035213	-0.032364	0.105667	0.301154
CpG_108	82,661,701	SBP	0.119477	0.238416	-0.34781	0.586764	0.619332

CpG_108	82,661,701	DBP	0.006257	0.180605	-0.347723	0.360237	0.972555
CpG_108	82,661,701	Total cholesterol	0.010222	0.01179	-0.012886	0.033331	0.388851
CpG_108	82,661,701	HDL	-0.000398	0.003748	-0.007744	0.006947	0.915642
CpG_108	82,661,701	LDL	0.012468	0.008766	-0.004713	0.029648	0.159305
CpG_108	82,661,701	Triglycerides	0.004377	0.007548	-0.010417	0.019171	0.563866
CpG_109	82,661,725	SBP	-1.78144	1.502681	-4.726641	1.163762	0.24204
CpG_109	82,661,725	DBP	-2.103862	1.089009	-4.238281	0.030556	0.059682
CpG_109	82,661,725	Total cholesterol	-0.067222	0.078156	-0.220404	0.08596	0.392271
CpG_109	82,661,725	HDL	0.008555	0.024619	-0.039697	0.056807	0.72911
CpG_109	82,661,725	LDL	-0.05939	0.060603	-0.178169	0.059389	0.330011
CpG_109	82,661,725	Triglycerides	0.016028	0.04923	-0.080461	0.112517	0.745599
CpG_116	82,661,812	SBP	0.021732	0.287048	-0.540871	0.584335	0.939986
CpG_116	82,661,812	DBP	-0.070342	0.212934	-0.487685	0.347	0.74267
CpG_116	82,661,812	Total cholesterol	0.017678	0.015801	-0.013292	0.048648	0.266544
CpG_116	82,661,812	HDL	0.005298	0.004962	-0.004428	0.015023	0.288851
CpG_116	82,661,812	LDL	0.026089	0.012019	0.002533	0.049646	0.032881
CpG_116	82,661,812	Triglycerides	-0.005521	0.010029	-0.025178	0.014135	0.583494

			CADCZ				
CpG site/unit[a]	Position at chr.16[b]	Trait	Beta	SE	CI_2.5%	CI_97.5%	*P* -value
CpG_1	82,660,317	SBP	-0.263373	0.189379	-0.634549	0.107804	0.166358
CpG_1	82,660,317	DBP	-0.129149	0.117327	-0.359107	0.100808	0.272753
CpG_1	82,660,317	Total cholesterol	-0.008137	0.011432	-0.030545	0.01427	0.477696
CpG_1	82,660,317	HDL	0.00661	0.003477	-0.000205	0.013424	0.059202
CpG_1	82,660,317	LDL	-0.014327	0.01038	-0.034672	0.006018	0.169558
CpG_1	82,660,317	Triglycerides	-0.009352	0.013022	-0.034875	0.01617	0.47374
CpG_2	82,660,328	SBP	-0.268157	0.193206	-0.646834	0.110519	0.167201
CpG_2	82,660,328	DBP	-0.065091	0.120058	-0.300399	0.170218	0.588505
CpG_2	82,660,328	Total cholesterol	0.002863	0.011766	-0.020197	0.025923	0.808063
CpG_2	82,660,328	HDL	0.001278	0.003614	-0.005805	0.008361	0.724021
CpG_2	82,660,328	LDL	-0.002868	0.010758	-0.023954	0.018218	0.790157
CpG_2	82,660,328	Triglycerides	0.006096	0.013395	-0.020158	0.03235	0.649696
CpG_3	82,660,376	SBP	-0.079248	0.225902	-0.522007	0.363512	0.726223
CpG_3	82,660,376	DBP	-0.005988	0.13968	-0.279755	0.267779	0.96586
CpG_3	82,660,376	Total cholesterol	-0.02869	0.013462	-0.055075	-0.002305	0.034681
CpG_3	82,660,376	HDL	0.010028	0.004117	0.001958	0.018098	0.016031
CpG_3	82,660,376	LDL	-0.022954	0.012373	-0.047205	0.001297	0.065528
CpG_3	82,660,376	Triglycerides	-0.035479	0.015293	-0.065453	-0.005506	0.021672
CpG_9	82,660,464	SBP	0.17842	0.145838	-0.107417	0.464258	0.223066
CpG_9	82,660,464	DBP	0.072464	0.090335	-0.104588	0.249516	0.423705
CpG_9	82,660,464	Total cholesterol	0.005457	0.008852	-0.011892	0.022807	0.538471
CpG_9	82,660,464	HDL	0.002557	0.00279	-0.00291	0.008024	0.360786
CpG_9	82,660,464	LDL	0.004802	0.008098	-0.01107	0.020674	0.554098
CpG_9	82,660,464	Triglycerides	-0.011302	0.010062	-0.031024	0.00842	0.263101
CpG_11	82,660,490	SBP	-0.051217	0.216455	-0.47546	0.373026	0.813282
CpG_11	82,660,490	DBP	-0.081348	0.135677	-0.34727	0.184573	0.549713
CpG_11	82,660,490	Total cholesterol	0.013682	0.012988	-0.011775	0.039138	0.293885
CpG_11	82,660,490	HDL	-0.000675	0.004138	-0.008786	0.007435	0.870593

CpG_11	82,660,490	LDL	0.02011	0.012091	-0.003588	0.043808	0.098388
CpG_11	82,660,490	Triglycerides	-0.002887	0.014061	-0.030447	0.024673	0.837611
CpG_12	82,660,505	SBP	0.542324	0.429483	-0.299447	1.384095	0.208617
CpG_12	82,660,505	DBP	0.179414	0.266279	-0.342484	0.701312	0.501474
CpG_12	82,660,505	Total cholesterol	0.019197	0.026051	-0.031863	0.070256	0.46232
CpG_12	82,660,505	HDL	-0.012114	0.008178	-0.028143	0.003915	0.140595
CpG_12	82,660,505	LDL	0.032155	0.023716	-0.014327	0.078637	0.177162
CpG_12	82,660,505	Triglycerides	0.008608	0.029743	-0.049688	0.066904	0.77266
CpG_13	82,660,554	SBP	0.67933	0.683252	-0.659818	2.018479	0.321676
CpG_13	82,660,554	DBP	0.59711	0.420631	-0.227311	1.421531	0.157784
CpG_13	82,660,554	Total cholesterol	0.03191	0.041191	-0.048823	0.112643	0.43972
CpG_13	82,660,554	HDL	-0.013385	0.012981	-0.038827	0.012057	0.304106
CpG_13	82,660,554	LDL	0.008374	0.037734	-0.065582	0.082331	0.824665
CpG_13	82,660,554	Triglycerides	0.014427	0.047036	-0.077762	0.106615	0.759475
CpG_15	82,660,630	SBP	0.746572	0.45478	-0.14478	1.637925	0.102808
CpG_15	82,660,630	DBP	0.418479	0.273931	-0.118417	0.955374	0.128741
CpG_15	82,660,630	Total cholesterol	0.033825	0.026587	-0.018284	0.085934	0.205274
CpG_15	82,660,630	HDL	-0.001987	0.007025	-0.015757	0.011782	0.777688
CpG_15	82,660,630	LDL	0.009508	0.024213	-0.037948	0.056965	0.695109
CpG_15	82,660,630	Triglycerides	-0.009255	0.029768	-0.067599	0.049089	0.756309
CpG_16-17	82,660,652-82,660,654	SBP	0.534412	0.37973	-0.209845	1.278669	0.161436
CpG_16-17	82,660,652-82,660,654	DBP	0.266154	0.228934	-0.182547	0.714856	0.246883
CpG_16-17	82,660,652-82,660,654	Total cholesterol	0.012362	0.022249	-0.031246	0.05597	0.579315
CpG_16-17	82,660,652-82,660,654	HDL	-0.006543	0.00583	-0.01797	0.004884	0.263557
CpG_16-17	82,660,652-82,660,654	LDL	0.018677	0.020075	-0.02067	0.058024	0.353728
CpG_16-17	82,660,652-82,660,654	Triglycerides	-0.027857	0.024704	-0.076276	0.020562	0.261306
CpG_22	82,660,690	SBP	0.702769	0.361155	-0.005081	1.410619	0.053575
CpG_22	82,660,690	DBP	0.325921	0.218407	-0.102149	0.753992	0.137773
CpG_22	82,660,690	Total cholesterol	0.03943	0.021039	-0.001805	0.080666	0.062878
CpG_22	82,660,690	HDL	-0.002886	0.005591	-0.013844	0.008072	0.606478
CpG_22	82,660,690	LDL	0.010085	0.01924	-0.027625	0.047794	0.600959
CpG_22	82,660,690	Triglycerides	0.003946	0.02371	-0.042525	0.050417	0.868055
CpG_23	82,660,705	SBP	0.416066	0.297718	-0.167451	0.999582	0.164364

CpG_23	82,660,705	DBP	0.281037	0.1788	-0.069405	0.631479	0.118148
CpG_23	82,660,705	Total cholesterol	0.03212	0.017254	-0.001698	0.065937	0.064646
CpG_23	82,660,705	HDL	-0.003753	0.004578	-0.012727	0.00522	0.41364
CpG_23	82,660,705	LDL	0.023218	0.015663	-0.007481	0.053917	0.140386
CpG_23	82,660,705	Triglycerides	-0.008695	0.019431	-0.046778	0.029388	0.65518
CpG_27-29	82,660,761-82,660,767	SBP	0.734126	0.550332	-0.344505	1.812758	0.184365
CpG_27-29	82,660,761-82,660,767	DBP	0.520789	0.327172	-0.120457	1.162035	0.113672
CpG_27-29	82,660,761-82,660,767	Total cholesterol	0.05131	0.031727	-0.010874	0.113493	0.108046
CpG_27-29	82,660,761-82,660,767	HDL	-0.007713	0.008481	-0.024336	0.008909	0.364639
CpG_27-29	82,660,761-82,660,767	LDL	0.042626	0.028321	-0.012882	0.098134	0.134537
CpG_27-29	82,660,761-82,660,767	Triglycerides	-0.008265	0.036123	-0.079064	0.062535	0.81936
CpG_30-31	82,660,774-82,660,779	SBP	0.495146	0.462789	-0.411903	1.402195	0.286422
CpG_30-31	82,660,774-82,660,779	DBP	0.269866	0.278299	-0.275589	0.815322	0.333799
CpG_30-31	82,660,774-82,660,779	Total cholesterol	0.020207	0.026966	-0.032645	0.073059	0.45483
CpG_30-31	82,660,774-82,660,779	HDL	-0.003469	0.007087	-0.017359	0.01042	0.625171
CpG_30-31	82,660,774-82,660,779	LDL	0.039763	0.02415	-0.007571	0.087097	0.101818
CpG_30-31	82,660,774-82,660,779	Triglycerides	-0.034416	0.030013	-0.093241	0.024408	0.253364
CpG_32	82,660,791	SBP	-0.191229	0.554607	-1.278238	0.89578	0.730797
CpG_32	82,660,791	DBP	-0.197709	0.331405	-0.84725	0.451833	0.551819
CpG_32	82,660,791	Total cholesterol	-0.05107	0.029381	-0.108656	0.006515	0.084507
CpG_32	82,660,791	HDL	0.007522	0.008434	-0.009007	0.024052	0.374044
CpG_32	82,660,791	LDL	-0.03806	0.026712	-0.090414	0.014294	0.156564
CpG_32	82,660,791	Triglycerides	-0.038336	0.032747	-0.102519	0.025847	0.243836
CpG_36	82,660,826	SBP	0.260839	0.47032	-0.660971	1.182648	0.580048
CpG_36	82,660,826	DBP	0.124796	0.283893	-0.431624	0.681216	0.660909
CpG_36	82,660,826	Total cholesterol	0.007255	0.02752	-0.046683	0.061192	0.79245
CpG_36	82,660,826	HDL	0.004084	0.007308	-0.010239	0.018408	0.57712
CpG_36	82,660,826	LDL	0.023804	0.024977	-0.025149	0.072758	0.342188
CpG_36	82,660,826	Triglycerides	-0.041749	0.030795	-0.102106	0.018607	0.17734
CpG_37	82,660,834	SBP	2.477404	1.478709	-0.420811	5.37562	0.095985
CpG_37	82,660,834	DBP	0.906371	0.894944	-0.847688	2.660429	0.312835
CpG_37	82,660,834	Total cholesterol	0.034614	0.086881	-0.135669	0.204898	0.690901
CpG_37	82,660,834	HDL	-0.019058	0.022798	-0.063741	0.025626	0.404534

CpG_37	82,660,834	LDL	0.039305	0.079345	-0.116209	0.194819	0.621081
CpG_37	82,660,834	Triglycerides	-0.025527	0.096809	-0.215269	0.164216	0.792394
CpG_38	82,660,843	SBP	0.246935	0.36795	-0.474234	0.968104	0.503306
CpG_38	82,660,843	DBP	-0.057459	0.227517	-0.503384	0.388465	0.801003
CpG_38	82,660,843	Total cholesterol	-0.000824	0.020528	-0.041058	0.03941	0.968029
CpG_38	82,660,843	HDL	-0.00753	0.005576	-0.018458	0.003399	0.179152
CpG_38	82,660,843	LDL	0.010678	0.018859	-0.026284	0.04764	0.572188
CpG_38	82,660,843	Triglycerides	-0.000527	0.022634	-0.044888	0.043835	0.981471
CpG_39-42	82,660,847-82,660,855	SBP	0.549104	0.281839	-0.003291	1.101499	0.053715
CpG_39-42	82,660,847-82,660,855	DBP	0.280932	0.168307	-0.048943	0.610807	0.097692
CpG_39-42	82,660,847-82,660,855	Total cholesterol	0.023106	0.015183	-0.006652	0.052864	0.130661
CpG_39-42	82,660,847-82,660,855	HDL	-0.001388	0.004283	-0.009782	0.007007	0.746506
CpG_39-42	82,660,847-82,660,855	LDL	0.012099	0.01323	-0.013831	0.038029	0.362262
CpG_39-42	82,660,847-82,660,855	Triglycerides	0.001501	0.015711	-0.029291	0.032294	0.924025
CpG_43	82,660,866	SBP	-0.084982	0.103983	-0.288785	0.118821	0.4151
CpG_43	82,660,866	DBP	-0.024806	0.062666	-0.147629	0.098017	0.692789
CpG_43	82,660,866	Total cholesterol	-0.012357	0.005976	-0.02407	-0.000643	0.040416
CpG_43	82,660,866	HDL	0.003104	0.001573	2.13E-05	0.006187	0.050301
CpG_43	82,660,866	LDL	-0.011973	0.005392	-0.02254	-0.001406	0.027902
CpG_43	82,660,866	Triglycerides	-0.009531	0.006707	-0.022676	0.003615	0.157427
CpG_45	82,660,890	SBP	0.435479	0.357369	-0.264952	1.13591	0.224973
CpG_45	82,660,890	DBP	0.373797	0.213437	-0.044533	0.792126	0.081991
CpG_45	82,660,890	Total cholesterol	0.023379	0.02072	-0.017231	0.063989	0.261014
CpG_45	82,660,890	HDL	-0.002778	0.005497	-0.013551	0.007995	0.613981
CpG_45	82,660,890	LDL	0.017955	0.018706	-0.018708	0.054618	0.338705
CpG_45	82,660,890	Triglycerides	-0.009883	0.023294	-0.055537	0.035772	0.671984
CpG_52	82,661,050	SBP	0.833668	0.704126	-0.546394	2.213729	0.238374
CpG_52	82,661,050	DBP	0.23099	0.431622	-0.614974	1.076954	0.593359
CpG_52	82,661,050	Total cholesterol	0.044263	0.042253	-0.038551	0.127076	0.296581
CpG_52	82,661,050	HDL	-0.011936	0.011681	-0.03483	0.010958	0.308544
CpG_52	82,661,050	LDL	0.049992	0.03828	-0.025036	0.12502	0.193657
CpG_52	82,661,050	Triglycerides	0.009285	0.046871	-0.082579	0.10115	0.843242
CpG_53	82,661,087	SBP	0.491559	0.4527	-0.395717	1.378835	0.279515

CpG_53	82,661,087	DBP	0.323055	0.266161	-0.198611	0.844721	0.226991
CpG_53	82,661,087	Total cholesterol	0.039868	0.025839	-0.010776	0.090512	0.125209
CpG_53	82,661,087	HDL	-0.010713	0.007194	-0.024812	0.003386	0.138784
CpG_53	82,661,087	LDL	0.025682	0.022729	-0.018866	0.07023	0.260546
CpG_53	82,661,087	Triglycerides	0.047994	0.029881	-0.01057	0.106559	0.110582
CpG_54	82,661,095	SBP	0.147554	0.690187	-1.205188	1.500295	0.831015
CpG_54	82,661,095	DBP	-0.176059	0.421263	-1.00172	0.649601	0.67662
CpG_54	82,661,095	Total cholesterol	-0.043605	0.041121	-0.124201	0.036991	0.290727
CpG_54	82,661,095	HDL	0.002436	0.011408	-0.019924	0.024795	0.831218
CpG_54	82,661,095	LDL	-0.033891	0.037379	-0.107153	0.039371	0.366093
CpG_54	82,661,095	Triglycerides	-0.026906	0.045571	-0.116223	0.062412	0.555833
CpG_57-58	82,661,139-82,661,141	SBP	-0.225779	0.607353	-1.416169	0.964612	0.710651
CpG_57-58	82,661,139-82,661,141	DBP	-0.055074	0.369086	-0.778468	0.668321	0.881599
CpG_57-58	82,661,139-82,661,141	Total cholesterol	-0.062828	0.034813	-0.131061	0.005405	0.073269
CpG_57-58	82,661,139-82,661,141	HDL	0.00977	0.009866	-0.009566	0.029106	0.323722
CpG_57-58	82,661,139-82,661,141	LDL	-0.048121	0.031029	-0.108937	0.012695	0.123216
CpG_57-58	82,661,139-82,661,141	Triglycerides	-0.055226	0.039691	-0.133018	0.022566	0.166306
CpG_62-64	82,661,175-82,661,188	SBP	0.108363	0.695459	-1.254712	1.471439	0.876413
CpG_62-64	82,661,175-82,661,188	DBP	-0.126918	0.4197	-0.949514	0.695678	0.762814
CpG_62-64	82,661,175-82,661,188	Total cholesterol	0.001013	0.039197	-0.075812	0.077838	0.979423
CpG_62-64	82,661,175-82,661,188	HDL	-0.010636	0.011095	-0.032382	0.01111	0.339461
CpG_62-64	82,661,175-82,661,188	LDL	-0.006097	0.035008	-0.07471	0.062517	0.862004
CpG_62-64	82,661,175-82,661,188	Triglycerides	0.031543	0.045404	-0.057447	0.120533	0.488425
CpG_66	82,661,202	SBP	0.142647	0.353305	-0.549818	0.835112	0.686999
CpG_66	82,661,202	DBP	0.066854	0.215859	-0.356223	0.489931	0.757231
CpG_66	82,661,202	Total cholesterol	0.033854	0.020631	-0.006582	0.074291	0.102993
CpG_66	82,661,202	HDL	-0.007375	0.005823	-0.018788	0.004039	0.207421
CpG_66	82,661,202	LDL	0.025397	0.018905	-0.011656	0.06245	0.181263
CpG_66	82,661,202	Triglycerides	0.033075	0.023135	-0.012268	0.078418	0.154972
CpG_67	82,661,217	SBP	0.166644	0.42106	-0.658618	0.991906	0.692871
CpG_67	82,661,217	DBP	0.044795	0.257703	-0.460295	0.549884	0.862254
CpG_67	82,661,217	Total cholesterol	0.004204	0.025613	-0.045996	0.054404	0.869856
CpG_67	82,661,217	HDL	0.010154	0.006983	-0.003532	0.023839	0.14811

CpG_67	82,661,217	LDL	0.011508	0.023225	-0.034012	0.057029	0.621018
CpG_67	82,661,217	Triglycerides	-0.032829	0.028142	-0.087986	0.022328	0.24535
CpG_69-70	82,661,244-82,661,248	SBP	0.560295	0.934932	-1.272138	2.392728	0.549984
CpG_69-70	82,661,244-82,661,248	DBP	-0.166063	0.56731	-1.277971	0.945844	0.770185
CpG_69-70	82,661,244-82,661,248	Total cholesterol	-0.045469	0.053532	-0.15039	0.059452	0.397163
CpG_69-70	82,661,244-82,661,248	HDL	0.000398	0.01501	-0.029021	0.029817	0.978904
CpG_69-70	82,661,244-82,661,248	LDL	-0.052197	0.047422	-0.145143	0.040748	0.272985
CpG_69-70	82,661,244-82,661,248	Triglycerides	0.00496	0.061823	-0.11621	0.12613	0.936175
CpG_71-72	82,661,267-82,661,275	SBP	0.068628	0.54072	-0.991163	1.12842	0.899188
CpG_71-72	82,661,267-82,661,275	DBP	-0.139369	0.328105	-0.782443	0.503705	0.671666
CpG_71-72	82,661,267-82,661,275	Total cholesterol	-0.032169	0.031069	-0.093062	0.028724	0.30227
CpG_71-72	82,661,267-82,661,275	HDL	-0.00876	0.008862	-0.026129	0.008608	0.324596
CpG_71-72	82,661,267-82,661,275	LDL	-0.028258	0.027501	-0.082159	0.025643	0.305974
CpG_71-72	82,661,267-82,661,275	Triglycerides	0.014181	0.035961	-0.0563	0.084663	0.693921
CpG_73-74	82,661,285-82,661,290	SBP	0.355753	0.349985	-0.330205	1.04171	0.311179
CpG_73-74	82,661,285-82,661,290	DBP	0.029526	0.21367	-0.389259	0.448311	0.890295
CpG_73-74	82,661,285-82,661,290	Total cholesterol	-0.01835	0.019998	-0.057546	0.020845	0.360419
CpG_73-74	82,661,285-82,661,290	HDL	-0.008066	0.005721	-0.019279	0.003146	0.160762
CpG_73-74	82,661,285-82,661,290	LDL	-0.006106	0.017934	-0.041257	0.029044	0.73401
CpG_73-74	82,661,285-82,661,290	Triglycerides	-0.006695	0.023151	-0.052071	0.038681	0.772865
CpG_75-76	82,661,313-82,661,326	SBP	0.564749	0.555803	-0.524605	1.654103	0.311443
CpG_75-76	82,661,313-82,661,326	DBP	0.119929	0.337393	-0.54135	0.781208	0.722815
CpG_75-76	82,661,313-82,661,326	Total cholesterol	0.025213	0.031641	-0.036803	0.087228	0.426968
CpG_75-76	82,661,313-82,661,326	HDL	0.003144	0.008616	-0.013743	0.020031	0.71575
CpG_75-76	82,661,313-82,661,326	LDL	0.045316	0.027783	-0.009138	0.099769	0.105259
CpG_75-76	82,661,313-82,661,326	Triglycerides	-0.047178	0.036515	-0.118746	0.024389	0.198586
CpG_77-81	82,661,336-82,661,349	SBP	0.869702	0.723515	-0.548361	2.287765	0.231402
CpG_77-81	82,661,336-82,661,349	DBP	-0.086942	0.440425	-0.950159	0.776276	0.843802
CpG_77-81	82,661,336-82,661,349	Total cholesterol	-0.033635	0.041544	-0.115059	0.047788	0.419529
CpG_77-81	82,661,336-82,661,349	HDL	-0.020498	0.011459	-0.042957	0.00196	0.075812
CpG_77-81	82,661,336-82,661,349	LDL	0.00234	0.036845	-0.069875	0.074555	0.949449
CpG_77-81	82,661,336-82,661,349	Triglycerides	-0.027873	0.047755	-0.121472	0.065725	0.560387
CpG_83	82,661,360	SBP	0.225214	0.32876	-0.419143	0.869571	0.494434

CpG_83	82,661,360	DBP	0.086087	0.199531	-0.304986	0.47716	0.666797
CpG_83	82,661,360	Total cholesterol	0.019272	0.019146	-0.018253	0.056796	0.315839
CpG_83	82,661,360	HDL	-0.008065	0.005369	-0.018587	0.002458	0.135281
CpG_83	82,661,360	LDL	0.02316	0.017006	-0.010172	0.056492	0.175413
CpG_83	82,661,360	Triglycerides	0.010418	0.021813	-0.032335	0.05317	0.633669
CpG_84-85	82,661,384-82,661,390	SBP	0.279558	0.366998	-0.439745	0.998861	0.447495
CpG_84-85	82,661,384-82,661,390	DBP	0.223755	0.221954	-0.211266	0.658776	0.315139
CpG_84-85	82,661,384-82,661,390	Total cholesterol	-0.024481	0.021395	-0.066414	0.017453	0.254471
CpG_84-85	82,661,384-82,661,390	HDL	-0.006707	0.006031	-0.018528	0.005114	0.268007
CpG_84-85	82,661,384-82,661,390	LDL	-0.015319	0.019108	-0.052771	0.022132	0.424077
CpG_84-85	82,661,384-82,661,390	Triglycerides	-0.002751	0.024361	-0.050498	0.044996	0.910239
CpG_86	82,661,399	SBP	-0.091269	0.282174	-0.64432	0.461782	0.746825
CpG_86	82,661,399	DBP	0.077914	0.172245	-0.259681	0.415508	0.651704
CpG_86	82,661,399	Total cholesterol	0.004094	0.016923	-0.029074	0.037262	0.809179
CpG_86	82,661,399	HDL	-0.010598	0.004595	-0.019604	-0.001592	0.022506
CpG_86	82,661,399	LDL	0.023295	0.015273	-0.006639	0.053229	0.129392
CpG_86	82,661,399	Triglycerides	-0.017275	0.018653	-0.053834	0.019284	0.355924
CpG_87-88	82,661,404-82,661,413	SBP	0.220375	0.371099	-0.506966	0.947716	0.553568
CpG_87-88	82,661,404-82,661,413	DBP	0.214288	0.226126	-0.22891	0.657487	0.344928
CpG_87-88	82,661,404-82,661,413	Total cholesterol	0.008764	0.022063	-0.03448	0.052008	0.691805
CpG_87-88	82,661,404-82,661,413	HDL	-0.009072	0.006095	-0.021017	0.002873	0.138843
CpG_87-88	82,661,404-82,661,413	LDL	0.024199	0.019813	-0.014634	0.063033	0.223989
CpG_87-88	82,661,404-82,661,413	Triglycerides	-0.012678	0.024571	-0.060837	0.035481	0.606679
CpG_89	82,661,421	SBP	0.466716	0.470231	-0.45492	1.388352	0.322691
CpG_89	82,661,421	DBP	0.182498	0.290497	-0.386866	0.751863	0.530901
CpG_89	82,661,421	Total cholesterol	0.070758	0.02846	0.014978	0.126538	0.014103
CpG_89	82,661,421	HDL	-0.005347	0.007946	-0.020921	0.010226	0.502082
CpG_89	82,661,421	LDL	0.07327	0.025654	0.022988	0.123551	0.004958
CpG_89	82,661,421	Triglycerides	0.006133	0.031979	-0.056544	0.06881	0.84819
CpG_90	82,661,438	SBP	0.089408	0.398745	-0.692118	0.870934	0.822902
CpG_90	82,661,438	DBP	0.192504	0.243001	-0.283769	0.668777	0.42955
CpG_90	82,661,438	Total cholesterol	0.017532	0.023851	-0.029215	0.06428	0.463482
CpG_90	82,661,438	HDL	-0.006984	0.006579	-0.019879	0.005911	0.290235

CpG_90	82,661,438	LDL	0.034154	0.021495	-0.007975	0.076284	0.114268
CpG_90	82,661,438	Triglycerides	-0.017663	0.02637	-0.069348	0.034022	0.504054
CpG_91	82,661,469	SBP	1.542745	0.63574	0.296717	2.788773	0.016557
CpG_91	82,661,469	DBP	0.789033	0.388214	0.028148	1.549919	0.044067
CpG_91	82,661,469	Total cholesterol	0.049163	0.037029	-0.023412	0.121738	0.186498
CpG_91	82,661,469	HDL	0.001039	0.011022	-0.020563	0.022641	0.925003
CpG_91	82,661,469	LDL	0.062813	0.033253	-0.002363	0.127988	0.061048
CpG_91	82,661,469	Triglycerides	-0.027463	0.044052	-0.113803	0.058877	0.534051
CpG_92	82,661,480	SBP	0.593052	0.544606	-0.474356	1.66046	0.278128
CpG_92	82,661,480	DBP	0.240867	0.3301	-0.406116	0.887851	0.466859
CpG_92	82,661,480	Total cholesterol	0.008931	0.03124	-0.052298	0.070159	0.775415
CpG_92	82,661,480	HDL	-0.000164	0.009179	-0.018155	0.017827	0.985761
CpG_92	82,661,480	LDL	0.01672	0.028487	-0.039112	0.072553	0.558222
CpG_92	82,661,480	Triglycerides	-0.020901	0.037118	-0.093651	0.051849	0.574302
CpG_104	82,661,633	SBP	0.110934	0.192085	-0.265546	0.487413	0.564609
CpG_104	82,661,633	DBP	0.032334	0.116681	-0.196357	0.261025	0.782144
CpG_104	82,661,633	Total cholesterol	-0.000111	0.010691	-0.021065	0.020843	0.991746
CpG_104	82,661,633	HDL	-0.004095	0.003186	-0.010339	0.002149	0.201017
CpG_104	82,661,633	LDL	0.005176	0.009523	-0.01349	0.023841	0.587768
CpG_104	82,661,633	Triglycerides	-0.001217	0.013035	-0.026765	0.024332	0.925791
CpG_106	82,661,670	SBP	0.506795	0.480051	-0.434087	1.447677	0.292999
CpG_106	82,661,670	DBP	0.212247	0.290894	-0.357895	0.782389	0.466886
CpG_106	82,661,670	Total cholesterol	-0.060506	0.02703	-0.113484	-0.007529	0.026825
CpG_106	82,661,670	HDL	-0.006759	0.008065	-0.022566	0.009048	0.403464
CpG_106	82,661,670	LDL	-0.028565	0.024823	-0.077217	0.020088	0.251901
CpG_106	82,661,670	Triglycerides	-0.046986	0.032484	-0.110654	0.016681	0.150372
CpG_107	82,661,696	SBP	0.019046	0.523099	-1.006208	1.044301	0.971013
CpG_107	82,661,696	DBP	-0.105573	0.310949	-0.715022	0.503876	0.734785
CpG_107	82,661,696	Total cholesterol	0.008017	0.029152	-0.049121	0.065155	0.783765
CpG_107	82,661,696	HDL	0.003881	0.008591	-0.012958	0.02072	0.652238
CpG_107	82,661,696	LDL	2.32E-05	0.02625	-0.051425	0.051472	0.999296
CpG_107	82,661,696	Triglycerides	0.007016	0.035394	-0.062355	0.076388	0.843181
CpG_108	82,661,701	SBP	0.140704	0.208824	-0.268584	0.549992	0.501658

CpG_108	82,661,701	DBP	0.094066	0.126686	-0.154235	0.342367	0.459138
CpG_108	82,661,701	Total cholesterol	-0.014666	0.011642	-0.037483	0.008151	0.210028
CpG_108	82,661,701	HDL	0.001342	0.003502	-0.005521	0.008205	0.702156
CpG_108	82,661,701	LDL	-0.006596	0.010506	-0.027187	0.013996	0.531264
CpG_108	82,661,701	Triglycerides	-0.017827	0.014192	-0.045642	0.009988	0.21132
CpG_109	82,661,725	SBP	0.283921	0.274002	-0.253112	0.820955	0.301974
CpG_109	82,661,725	DBP	0.047927	0.166289	-0.277993	0.373846	0.773628
CpG_109	82,661,725	Total cholesterol	-0.011911	0.015653	-0.042591	0.018768	0.448011
CpG_109	82,661,725	HDL	-0.000122	0.004608	-0.009154	0.008909	0.978855
CpG_109	82,661,725	LDL	0.002755	0.014141	-0.02496	0.03047	0.845819
CpG_109	82,661,725	Triglycerides	-0.028507	0.018493	-0.064751	0.007738	0.125528
CpG_116	82,661,812	SBP	0.036431	0.275117	-0.502788	0.575649	0.894851
CpG_116	82,661,812	DBP	-0.005844	0.166362	-0.331907	0.32022	0.972031
CpG_116	82,661,812	Total cholesterol	-0.042729	0.015267	-0.072651	-0.012807	0.00588
CpG_116	82,661,812	HDL	0.005516	0.004588	-0.003477	0.014509	0.231427
CpG_116	82,661,812	LDL	-0.037989	0.013787	-0.065012	-0.010966	0.006679
CpG_116	82,661,812	Triglycerides	-0.019331	0.0186	-0.055787	0.017124	0.300519

CpG site/unit[a]	Position at chr.16[b]	Trait	Meta-analysis[d]				
			Beta	SE	CI_2.5%	CI_97.5%	*P* -value
CpG_1	82,660,317	SBP	-0.30693	0.158644	-0.617866	0.004006	0.053026
CpG_1	82,660,317	DBP	-0.10574	0.099056	-0.299886	0.088405	0.285754
CpG_1	82,660,317	Total cholesterol	-0.004402	0.008907	-0.021859	0.013056	0.621175
CpG_1	82,660,317	HDL	0.005611	0.00275	0.000222	0.011001	0.041277
CpG_1	82,660,317	LDL	-0.009268	0.007487	-0.023942	0.005407	0.215799
CpG_1	82,660,317	Triglycerides	0.003989	0.009515	-0.01466	0.022638	0.675029
CpG_2	82,660,328	SBP	-0.398172	0.158584	-0.708991	-0.087353	0.012046
CpG_2	82,660,328	DBP	-0.149799	0.099418	-0.344656	0.045057	0.131874
CpG_2	82,660,328	Total cholesterol	0.004964	0.009061	-0.012794	0.022722	0.583786
CpG_2	82,660,328	HDL	0.001083	0.00282	-0.004444	0.00661	0.700972
CpG_2	82,660,328	LDL	-0.00305	0.007622	-0.017989	0.011889	0.689035
CpG_2	82,660,328	Triglycerides	0.003769	0.009719	-0.015279	0.022818	0.698127
CpG_3	82,660,376	SBP	-0.063662	0.19622	-0.448246	0.320921	0.745601
CpG_3	82,660,376	DBP	0.03179	0.121897	-0.207124	0.270703	0.794254
CpG_3	82,660,376	Total cholesterol	-0.020573	0.010901	-0.041939	0.000794	0.05914
CpG_3	82,660,376	HDL	0.005712	0.003374	-0.000901	0.012326	0.090483
CpG_3	82,660,376	LDL	-0.018709	0.009296	-0.03693	-0.000489	0.044165
CpG_3	82,660,376	Triglycerides	-0.021742	0.011735	-0.044742	0.001259	0.063926
CpG_9	82,660,464	SBP	0.146316	0.140346	-0.128757	0.42139	0.297162
CpG_9	82,660,464	DBP	0.069249	0.087045	-0.101356	0.239854	0.426293
CpG_9	82,660,464	Total cholesterol	0.011679	0.008356	-0.004698	0.028056	0.162211
CpG_9	82,660,464	HDL	0.003052	0.00264	-0.002121	0.008226	0.247505
CpG_9	82,660,464	LDL	0.011567	0.007467	-0.003067	0.026201	0.121342
CpG_9	82,660,464	Triglycerides	-0.006217	0.009353	-0.024549	0.012114	0.506212
CpG_11	82,660,490	SBP	-0.001132	0.163209	-0.321016	0.318751	0.994464
CpG_11	82,660,490	DBP	0.032657	0.102116	-0.167486	0.232801	0.749115
CpG_11	82,660,490	Total cholesterol	0.016321	0.009344	-0.001993	0.034634	0.080695
CpG_11	82,660,490	HDL	0.00095	0.002988	-0.004906	0.006807	0.750517

CpG_11	82,660,490	LDL	0.010598	0.00787	-0.004828	0.026023	0.178119
CpG_11	82,660,490	Triglycerides	-0.009171	0.009757	-0.028294	0.009952	0.347241
CpG_12	82,660,505	SBP	0.341013	0.375626	-0.3952	1.077226	0.363956
CpG_12	82,660,505	DBP	0.115955	0.233764	-0.342214	0.574124	0.619869
CpG_12	82,660,505	Total cholesterol	0.019561	0.021048	-0.021692	0.060815	0.352708
CpG_12	82,660,505	HDL	-0.011467	0.006639	-0.024478	0.001545	0.08413
CpG_12	82,660,505	LDL	0.033756	0.017871	-0.00127	0.068783	0.058908
CpG_12	82,660,505	Triglycerides	-0.008494	0.022717	-0.053018	0.03603	0.708465
CpG_13	82,660,554	SBP	0.698009	0.620878	-0.518889	1.914908	0.260916
CpG_13	82,660,554	DBP	0.573394	0.383725	-0.178692	1.32548	0.135101
CpG_13	82,660,554	Total cholesterol	0.026727	0.036189	-0.044202	0.097655	0.460189
CpG_13	82,660,554	HDL	-0.003148	0.011402	-0.025496	0.0192	0.782477
CpG_13	82,660,554	LDL	0.000413	0.031597	-0.061516	0.062342	0.989579
CpG_13	82,660,554	Triglycerides	0.012495	0.039794	-0.065499	0.090489	0.753521
CpG_15	82,660,630	SBP	0.590578	0.428602	-0.249467	1.430623	0.168229
CpG_15	82,660,630	DBP	0.251523	0.259681	-0.257441	0.760488	0.332751
CpG_15	82,660,630	Total cholesterol	0.041188	0.025132	-0.00807	0.090445	0.101242
CpG_15	82,660,630	HDL	-0.000693	0.006784	-0.01399	0.012604	0.918599
CpG_15	82,660,630	LDL	0.021752	0.022372	-0.022096	0.0656	0.330897
CpG_15	82,660,630	Triglycerides	-0.018004	0.027799	-0.072489	0.036481	0.517211
CpG_16-17	82,660,652-82,660,654	SBP	0.54396	0.366738	-0.174834	1.262754	0.138012
CpG_16-17	82,660,652-82,660,654	DBP	0.285912	0.221997	-0.149196	0.721019	0.197779
CpG_16-17	82,660,652-82,660,654	Total cholesterol	0.01483	0.021094	-0.026513	0.056173	0.482018
CpG_16-17	82,660,652-82,660,654	HDL	-0.006266	0.005642	-0.017325	0.004793	0.266781
CpG_16-17	82,660,652-82,660,654	LDL	0.012495	0.018654	-0.024067	0.049057	0.502967
CpG_16-17	82,660,652-82,660,654	Triglycerides	-0.016588	0.023167	-0.061993	0.028818	0.473979
CpG_22	82,660,690	SBP	0.600287	0.33581	-0.057888	1.258461	0.073844
CpG_22	82,660,690	DBP	0.24048	0.204644	-0.160615	0.641574	0.239949
CpG_22	82,660,690	Total cholesterol	0.041553	0.019483	0.003367	0.079738	0.032943
CpG_22	82,660,690	HDL	-0.000227	0.005322	-0.010658	0.010204	0.966012
CpG_22	82,660,690	LDL	0.015509	0.017281	-0.018362	0.04938	0.369492
CpG_22	82,660,690	Triglycerides	-0.002373	0.021568	-0.044646	0.0399	0.912392
CpG_23	82,660,705	SBP	0.34439	0.293511	-0.230881	0.919661	0.240657

CpG_23	82,660,705	DBP	0.229645	0.176568	-0.116421	0.575711	0.193393
CpG_23	82,660,705	Total cholesterol	0.032912	0.017008	-0.000423	0.066247	0.052978
CpG_23	82,660,705	HDL	-0.003545	0.004538	-0.012439	0.005348	0.434624
CpG_23	82,660,705	LDL	0.022686	0.015349	-0.007397	0.05277	0.139392
CpG_23	82,660,705	Triglycerides	-0.00884	0.019083	-0.046243	0.028562	0.643186
CpG_27-29	82,660,761-82,660,767	SBP	0.544607	0.489059	-0.41393	1.503144	0.265458
CpG_27-29	82,660,761-82,660,767	DBP	0.374742	0.295667	-0.204754	0.954238	0.204995
CpG_27-29	82,660,761-82,660,767	Total cholesterol	0.033222	0.028081	-0.021816	0.088261	0.236779
CpG_27-29	82,660,761-82,660,767	HDL	-0.015755	0.007795	-0.031032	-0.000478	0.043249
CpG_27-29	82,660,761-82,660,767	LDL	0.039738	0.0241	-0.007498	0.086973	0.099179
CpG_27-29	82,660,761-82,660,767	Triglycerides	0.005853	0.030977	-0.054861	0.066567	0.850146
CpG_30-31	82,660,774-82,660,779	SBP	0.507995	0.459972	-0.393533	1.409523	0.269417
CpG_30-31	82,660,774-82,660,779	DBP	0.287138	0.276811	-0.255401	0.829678	0.299592
CpG_30-31	82,660,774-82,660,779	Total cholesterol	0.018422	0.026799	-0.034104	0.070947	0.49183
CpG_30-31	82,660,774-82,660,779	HDL	-0.0035	0.00706	-0.017338	0.010337	0.62004
CpG_30-31	82,660,774-82,660,779	LDL	0.035291	0.023943	-0.011636	0.082219	0.140486
CpG_30-31	82,660,774-82,660,779	Triglycerides	-0.022796	0.029772	-0.081148	0.035557	0.443869
CpG_32	82,660,791	SBP	-0.54208	0.473913	-1.470932	0.386772	0.25269
CpG_32	82,660,791	DBP	-0.231357	0.290791	-0.801298	0.338583	0.426257
CpG_32	82,660,791	Total cholesterol	-0.045592	0.026462	-0.097457	0.006272	0.0849
CpG_32	82,660,791	HDL	0.013012	0.007632	-0.001947	0.027971	0.088225
CpG_32	82,660,791	LDL	-0.03992	0.023009	-0.085017	0.005176	0.082737
CpG_32	82,660,791	Triglycerides	-0.038376	0.028779	-0.094782	0.01803	0.182382
CpG_36	82,660,826	SBP	0.181269	0.391056	-0.585187	0.947725	0.64298
CpG_36	82,660,826	DBP	0.067154	0.240716	-0.404641	0.538949	0.780264
CpG_36	82,660,826	Total cholesterol	0.010064	0.023685	-0.036358	0.056486	0.670905
CpG_36	82,660,826	HDL	0.003976	0.006457	-0.00868	0.016632	0.538076
CpG_36	82,660,826	LDL	0.027781	0.020315	-0.012036	0.067598	0.171462
CpG_36	82,660,826	Triglycerides	-0.028317	0.025575	-0.078443	0.021809	0.268201
CpG_37	82,660,834	SBP	1.647919	1.36543	-1.028275	4.324114	0.227476
CpG_37	82,660,834	DBP	0.538005	0.833542	-1.095707	2.171717	0.518639
CpG_37	82,660,834	Total cholesterol	0.04097	0.078085	-0.112074	0.194014	0.599805
CpG_37	82,660,834	HDL	-0.02593	0.021305	-0.067687	0.015826	0.223557

CpG_37	82,660,834	LDL	0.011405	0.068471	-0.122794	0.145605	0.867705
CpG_37	82,660,834	Triglycerides	0.009432	0.085175	-0.157507	0.176371	0.911823
CpG_38	82,660,843	SBP	0.136913	0.313411	-0.477362	0.751188	0.662222
CpG_38	82,660,843	DBP	-0.024939	0.195998	-0.409089	0.359211	0.898748
CpG_38	82,660,843	Total cholesterol	-0.004471	0.01761	-0.038987	0.030045	0.799589
CpG_38	82,660,843	HDL	-0.003928	0.005034	-0.013795	0.005939	0.435223
CpG_38	82,660,843	LDL	0.002291	0.015247	-0.027592	0.032174	0.880571
CpG_38	82,660,843	Triglycerides	-0.014478	0.018924	-0.051568	0.022612	0.444229
CpG_39-42	82,660,847-82,660,855	SBP	0.424747	0.273014	-0.11035	0.959844	0.119762
CpG_39-42	82,660,847-82,660,855	DBP	0.227774	0.163777	-0.093224	0.548771	0.1643
CpG_39-42	82,660,847-82,660,855	Total cholesterol	0.02509	0.014714	-0.003748	0.053928	0.088146
CpG_39-42	82,660,847-82,660,855	HDL	-0.000165	0.004188	-0.008373	0.008043	0.968628
CpG_39-42	82,660,847-82,660,855	LDL	0.011831	0.012703	-0.013067	0.036729	0.35167
CpG_39-42	82,660,847-82,660,855	Triglycerides	0.001053	0.015196	-0.028731	0.030838	0.944735
CpG_43	82,660,866	SBP	-0.074058	0.102273	-0.274509	0.126393	0.468991
CpG_43	82,660,866	DBP	-0.030145	0.061757	-0.151187	0.090897	0.625463
CpG_43	82,660,866	Total cholesterol	-0.012281	0.005755	-0.023561	-0.001002	0.032843
CpG_43	82,660,866	HDL	0.003042	0.001537	3E-05	0.006055	0.047762
CpG_43	82,660,866	LDL	-0.011511	0.005117	-0.021541	-0.001481	0.024493
CpG_43	82,660,866	Triglycerides	-0.010248	0.006398	-0.022789	0.002292	0.109226
CpG_45	82,660,890	SBP	0.539311	0.279292	-0.008091	1.086712	0.053484
CpG_45	82,660,890	DBP	0.270013	0.172253	-0.067597	0.607623	0.116989
CpG_45	82,660,890	Total cholesterol	0.005592	0.015774	-0.025325	0.036508	0.722972
CpG_45	82,660,890	HDL	-0.000427	0.004569	-0.009382	0.008528	0.925562
CpG_45	82,660,890	LDL	0.015485	0.013162	-0.010313	0.041282	0.239419
CpG_45	82,660,890	Triglycerides	-0.014544	0.01694	-0.047745	0.018656	0.390557
CpG_52	82,661,050	SBP	0.776308	0.450689	-0.107028	1.659643	0.084981
CpG_52	82,661,050	DBP	0.460499	0.302604	-0.132594	1.053593	0.128062
CpG_52	82,661,050	Total cholesterol	0.03481	0.02461	-0.013424	0.083045	0.157222
CpG_52	82,661,050	HDL	-0.00174	0.008253	-0.017915	0.014435	0.833035
CpG_52	82,661,050	LDL	0.031213	0.021708	-0.011333	0.073759	0.150471
CpG_52	82,661,050	Triglycerides	-0.00416	0.021726	-0.046742	0.038422	0.848155
CpG_53	82,661,087	SBP	0.584293	0.383029	-0.166429	1.335016	0.127145

CpG_53	82,661,087	DBP	0.380154	0.235853	-0.08211	0.842417	0.107
CpG_53	82,661,087	Total cholesterol	0.024373	0.019575	-0.013992	0.062739	0.213078
CpG_53	82,661,087	HDL	-0.013952	0.006085	-0.025878	-0.002026	0.021855
CpG_53	82,661,087	LDL	0.016387	0.017162	-0.017249	0.050024	0.339645
CpG_53	82,661,087	Triglycerides	0.045285	0.018127	0.009757	0.080813	0.012483
CpG_54	82,661,095	SBP	0.258438	0.394574	-0.514914	1.031789	0.512482
CpG_54	82,661,095	DBP	-0.180386	0.26934	-0.708284	0.347511	0.503027
CpG_54	82,661,095	Total cholesterol	-0.009086	0.021446	-0.051118	0.032947	0.671812
CpG_54	82,661,095	HDL	-0.004719	0.007379	-0.019181	0.009743	0.522469
CpG_54	82,661,095	LDL	-0.009952	0.018833	-0.046864	0.02696	0.597201
CpG_54	82,661,095	Triglycerides	0.002613	0.01845	-0.033549	0.038775	0.887371
CpG_57-58	82,661,139-82,661,141	SBP	0.035831	0.257947	-0.469735	0.541397	0.889522
CpG_57-58	82,661,139-82,661,141	DBP	0.172002	0.178585	-0.178018	0.522023	0.335478
CpG_57-58	82,661,139-82,661,141	Total cholesterol	-0.004489	0.014189	-0.032298	0.02332	0.751724
CpG_57-58	82,661,139-82,661,141	HDL	0.001488	0.004857	-0.008032	0.011008	0.759376
CpG_57-58	82,661,139-82,661,141	LDL	-0.002789	0.01239	-0.027073	0.021495	0.821917
CpG_57-58	82,661,139-82,661,141	Triglycerides	-0.000191	0.011623	-0.022972	0.02259	0.986889
CpG_62-64	82,661,175-82,661,188	SBP	-0.637326	0.414659	-1.450042	0.17539	0.124296
CpG_62-64	82,661,175-82,661,188	DBP	-0.602219	0.273646	-1.138555	-0.065883	0.027756
CpG_62-64	82,661,175-82,661,188	Total cholesterol	-0.017463	0.02048	-0.057603	0.022677	0.393832
CpG_62-64	82,661,175-82,661,188	HDL	-0.000103	0.006792	-0.013415	0.013208	0.987874
CpG_62-64	82,661,175-82,661,188	LDL	-0.025614	0.01798	-0.060854	0.009625	0.15427
CpG_62-64	82,661,175-82,661,188	Triglycerides	0.006507	0.017476	-0.027746	0.040759	0.709652
CpG_66	82,661,202	SBP	-0.080206	0.282295	-0.633494	0.473081	0.776317
CpG_66	82,661,202	DBP	-0.05318	0.18247	-0.410815	0.304454	0.770709
CpG_66	82,661,202	Total cholesterol	0.022103	0.016156	-0.009563	0.053769	0.171289
CpG_66	82,661,202	HDL	-0.006893	0.005027	-0.016746	0.00296	0.170341
CpG_66	82,661,202	LDL	0.016494	0.014504	-0.011933	0.04492	0.255446
CpG_66	82,661,202	Triglycerides	0.02192	0.015518	-0.008496	0.052335	0.157801
CpG_67	82,661,217	SBP	0.053645	0.397008	-0.724477	0.831767	0.892515
CpG_67	82,661,217	DBP	-0.031513	0.247046	-0.515713	0.452688	0.898499
CpG_67	82,661,217	Total cholesterol	-0.01113	0.023758	-0.057695	0.035436	0.639463
CpG_67	82,661,217	HDL	0.009718	0.006714	-0.00344	0.022877	0.147752

CpG_67	82,661,217	LDL	-0.008702	0.021387	-0.050621	0.033216	0.684081
CpG_67	82,661,217	Triglycerides	-0.032505	0.024722	-0.08096	0.01595	0.188578
CpG_69-70	82,661,244-82,661,248	SBP	-0.254228	0.637022	-1.502769	0.994312	0.689828
CpG_69-70	82,661,244-82,661,248	DBP	-0.324185	0.415519	-1.138586	0.490217	0.435278
CpG_69-70	82,661,244-82,661,248	Total cholesterol	-0.07068	0.034625	-0.138543	-0.002818	0.041217
CpG_69-70	82,661,244-82,661,248	HDL	-0.003151	0.010873	-0.024463	0.01816	0.771945
CpG_69-70	82,661,244-82,661,248	LDL	-0.076236	0.030041	-0.135115	-0.017357	0.011156
CpG_69-70	82,661,244-82,661,248	Triglycerides	-0.016471	0.031481	-0.078172	0.045231	0.600839
CpG_71-72	82,661,267-82,661,275	SBP	-0.011715	0.455433	-0.904346	0.880917	0.979479
CpG_71-72	82,661,267-82,661,275	DBP	-0.136927	0.289296	-0.703935	0.430082	0.635992
CpG_71-72	82,661,267-82,661,275	Total cholesterol	-0.047923	0.02491	-0.096744	0.000899	0.054372
CpG_71-72	82,661,267-82,661,275	HDL	-0.001588	0.007756	-0.016789	0.013613	0.837759
CpG_71-72	82,661,267-82,661,275	LDL	-0.048714	0.021837	-0.091514	-0.005914	0.025696
CpG_71-72	82,661,267-82,661,275	Triglycerides	-0.011491	0.024684	-0.05987	0.036889	0.641564
CpG_73-74	82,661,285-82,661,290	SBP	0.154236	0.342114	-0.516295	0.824766	0.652111
CpG_73-74	82,661,285-82,661,290	DBP	-0.059846	0.210259	-0.471946	0.352254	0.775928
CpG_73-74	82,661,285-82,661,290	Total cholesterol	-0.019336	0.019271	-0.057106	0.018434	0.315664
CpG_73-74	82,661,285-82,661,290	HDL	-0.006502	0.005576	-0.017431	0.004426	0.243564
CpG_73-74	82,661,285-82,661,290	LDL	-0.008773	0.01722	-0.042523	0.024976	0.610399
CpG_73-74	82,661,285-82,661,290	Triglycerides	-0.005296	0.021482	-0.0474	0.036808	0.805265
CpG_75-76	82,661,313-82,661,326	SBP	0.195127	0.488148	-0.761626	1.151879	0.689357
CpG_75-76	82,661,313-82,661,326	DBP	0.062095	0.307293	-0.540187	0.664378	0.83986
CpG_75-76	82,661,313-82,661,326	Total cholesterol	0.006535	0.026003	-0.04443	0.057501	0.801562
CpG_75-76	82,661,313-82,661,326	HDL	0.008866	0.007592	-0.006013	0.023745	0.242863
CpG_75-76	82,661,313-82,661,326	LDL	0.007726	0.02271	-0.036784	0.052236	0.733707
CpG_75-76	82,661,313-82,661,326	Triglycerides	-0.024492	0.025733	-0.074928	0.025944	0.341215
CpG_77-81	82,661,336-82,661,349	SBP	0.18258	0.518009	-0.832699	1.197858	0.72449
CpG_77-81	82,661,336-82,661,349	DBP	-0.21093	0.33805	-0.873495	0.451635	0.532653
CpG_77-81	82,661,336-82,661,349	Total cholesterol	-0.061271	0.030547	-0.121143	-0.001399	0.044882
CpG_77-81	82,661,336-82,661,349	HDL	-0.018769	0.009591	-0.037567	3.03E-05	0.05037
CpG_77-81	82,661,336-82,661,349	LDL	-0.033423	0.026862	-0.086071	0.019224	0.213397
CpG_77-81	82,661,336-82,661,349	Triglycerides	-0.028023	0.02922	-0.085293	0.029246	0.337532
CpG_83	82,661,360	SBP	0.087189	0.236481	-0.376305	0.550684	0.712355

CpG_83	82,661,360	DBP	-0.075749	0.154258	-0.378088	0.22659	0.623386
CpG_83	82,661,360	Total cholesterol	-0.003121	0.010889	-0.024463	0.018221	0.774393
CpG_83	82,661,360	HDL	0.000126	0.003488	-0.006711	0.006962	0.971291
CpG_83	82,661,360	LDL	-0.003958	0.009403	-0.022388	0.014473	0.673846
CpG_83	82,661,360	Triglycerides	-0.013772	0.009481	-0.032354	0.00481	0.146325
CpG_84-85	82,661,384-82,661,390	SBP	0.09653	0.227182	-0.348738	0.541798	0.670907
CpG_84-85	82,661,384-82,661,390	DBP	0.164077	0.152133	-0.134098	0.462251	0.280807
CpG_84-85	82,661,384-82,661,390	Total cholesterol	-0.009715	0.0124	-0.034018	0.014588	0.43335
CpG_84-85	82,661,384-82,661,390	HDL	-0.002885	0.004193	-0.011104	0.005333	0.491392
CpG_84-85	82,661,384-82,661,390	LDL	-0.003437	0.010863	-0.024728	0.017853	0.751674
CpG_84-85	82,661,384-82,661,390	Triglycerides	-0.001907	0.01087	-0.023213	0.019398	0.860717
CpG_86	82,661,399	SBP	-0.067136	0.114733	-0.292008	0.157737	0.558449
CpG_86	82,661,399	DBP	-0.026801	0.08098	-0.18552	0.131918	0.740677
CpG_86	82,661,399	Total cholesterol	-0.002559	0.006991	-0.016262	0.011144	0.714377
CpG_86	82,661,399	HDL	-0.002476	0.002484	-0.007345	0.002393	0.318991
CpG_86	82,661,399	LDL	0.000276	0.006091	-0.011663	0.012214	0.963904
CpG_86	82,661,399	Triglycerides	-0.007769	0.00585	-0.019235	0.003697	0.184159
CpG_87-88	82,661,404-82,661,413	SBP	0.005633	0.254644	-0.493459	0.504725	0.982351
CpG_87-88	82,661,404-82,661,413	DBP	0.145994	0.169045	-0.185328	0.477316	0.387786
CpG_87-88	82,661,404-82,661,413	Total cholesterol	0.018831	0.014489	-0.009567	0.047228	0.193714
CpG_87-88	82,661,404-82,661,413	HDL	-0.004646	0.004722	-0.013901	0.00461	0.325261
CpG_87-88	82,661,404-82,661,413	LDL	0.021103	0.012742	-0.003871	0.046078	0.097693
CpG_87-88	82,661,404-82,661,413	Triglycerides	-0.001281	0.013196	-0.027145	0.024582	0.922639
CpG_89	82,661,421	SBP	0.119111	0.394339	-0.65378	0.892001	0.762613
CpG_89	82,661,421	DBP	0.015652	0.251784	-0.477836	0.50914	0.950432
CpG_89	82,661,421	Total cholesterol	0.033512	0.022865	-0.011302	0.078327	0.142743
CpG_89	82,661,421	HDL	-0.007679	0.006964	-0.021328	0.00597	0.270154
CpG_89	82,661,421	LDL	0.042427	0.020469	0.002308	0.082545	0.038197
CpG_89	82,661,421	Triglycerides	-0.013206	0.021949	-0.056226	0.029814	0.5474
CpG_90	82,661,438	SBP	-0.029149	0.32715	-0.67035	0.612053	0.929003
CpG_90	82,661,438	DBP	0.045671	0.209682	-0.365297	0.45664	0.827575
CpG_90	82,661,438	Total cholesterol	0.013897	0.018155	-0.021687	0.049481	0.444005
CpG_90	82,661,438	HDL	-0.00552	0.005614	-0.016523	0.005484	0.325542

CpG_90	82,661,438	LDL	0.019487	0.016116	-0.0121	0.051073	0.226603
CpG_90	82,661,438	Triglycerides	-0.007209	0.017185	-0.040891	0.026473	0.674865
CpG_91	82,661,469	SBP	0.392981	0.288586	-0.172637	0.958599	0.173277
CpG_91	82,661,469	DBP	0.298113	0.204261	-0.102231	0.698457	0.144435
CpG_91	82,661,469	Total cholesterol	0.005428	0.018084	-0.030017	0.040872	0.764079
CpG_91	82,661,469	HDL	-0.000943	0.005603	-0.011925	0.010039	0.866363
CpG_91	82,661,469	LDL	0.013874	0.014494	-0.014535	0.042282	0.338472
CpG_91	82,661,469	Triglycerides	-0.007586	0.012466	-0.032018	0.016847	0.542845
CpG_92	82,661,480	SBP	0.576062	0.529497	-0.461733	1.613857	0.27662
CpG_92	82,661,480	DBP	0.293021	0.32379	-0.341596	0.927637	0.36548
CpG_92	82,661,480	Total cholesterol	0.006597	0.029841	-0.05189	0.065084	0.825033
CpG_92	82,661,480	HDL	0.004561	0.008801	-0.012689	0.021811	0.604309
CpG_92	82,661,480	LDL	0.014094	0.026771	-0.038376	0.066563	0.598563
CpG_92	82,661,480	Triglycerides	-0.043891	0.031858	-0.106332	0.01855	0.168299
CpG_104	82,661,633	SBP	0.056109	0.176871	-0.290552	0.40277	0.751068
CpG_104	82,661,633	DBP	0.05982	0.110221	-0.156209	0.275849	0.587316
CpG_104	82,661,633	Total cholesterol	-0.003952	0.009846	-0.02325	0.015346	0.688149
CpG_104	82,661,633	HDL	-0.002799	0.002953	-0.008588	0.002989	0.343195
CpG_104	82,661,633	LDL	0.001247	0.008559	-0.015529	0.018022	0.884199
CpG_104	82,661,633	Triglycerides	0.001125	0.010056	-0.018586	0.020835	0.910965
CpG_106	82,661,670	SBP	0.589039	0.371796	-0.139669	1.317746	0.113124
CpG_106	82,661,670	DBP	0.168348	0.243232	-0.308378	0.645073	0.488856
CpG_106	82,661,670	Total cholesterol	-0.032179	0.020728	-0.072805	0.008448	0.120566
CpG_106	82,661,670	HDL	-0.00766	0.0063	-0.020006	0.004687	0.224027
CpG_106	82,661,670	LDL	-0.008777	0.017634	-0.04334	0.025785	0.618657
CpG_106	82,661,670	Triglycerides	0.020262	0.016809	-0.012683	0.053208	0.228041
CpG_107	82,661,696	SBP	-0.167186	0.46358	-1.075786	0.741415	0.718368
CpG_107	82,661,696	DBP	-0.139103	0.287079	-0.701767	0.423561	0.627997
CpG_107	82,661,696	Total cholesterol	0.01936	0.02572	-0.03105	0.06977	0.451615
CpG_107	82,661,696	HDL	0.006421	0.007725	-0.008719	0.021561	0.405849
CpG_107	82,661,696	LDL	0.006089	0.022346	-0.037708	0.049886	0.785244
CpG_107	82,661,696	Triglycerides	0.02191	0.024963	-0.027016	0.070837	0.380104
CpG_108	82,661,701	SBP	0.131489	0.157088	-0.176397	0.439375	0.402569

CpG_108	82,661,701	DBP	0.065109	0.103715	-0.138168	0.268386	0.530156
CpG_108	82,661,701	Total cholesterol	-0.00238	0.008284	-0.018616	0.013857	0.773922
CpG_108	82,661,701	HDL	0.000531	0.002559	-0.004484	0.005546	0.835641
CpG_108	82,661,701	LDL	0.004644	0.006731	-0.008548	0.017836	0.490227
CpG_108	82,661,701	Triglycerides	-0.000519	0.006664	-0.013581	0.012542	0.937883
CpG_109	82,661,725	SBP	0.217461	0.269557	-0.310862	0.745783	0.41982
CpG_109	82,661,725	DBP	-0.001102	0.164383	-0.323287	0.321083	0.99465
CpG_109	82,661,725	Total cholesterol	-0.014044	0.015348	-0.044127	0.016038	0.360168
CpG_109	82,661,725	HDL	0.000171	0.004529	-0.008706	0.009049	0.969821
CpG_109	82,661,725	LDL	-0.000454	0.013771	-0.027444	0.026536	0.973718
CpG_109	82,661,725	Triglycerides	-0.023	0.017311	-0.05693	0.01093	0.183981
CpG_116	82,661,812	SBP	0.029393	0.198621	-0.359897	0.418683	0.882353
CpG_116	82,661,812	DBP	-0.030291	0.131095	-0.287233	0.22665	0.817266
CpG_116	82,661,812	Total cholesterol	-0.013565	0.010979	-0.035084	0.007954	0.216643
CpG_116	82,661,812	HDL	0.005415	0.003369	-0.001188	0.012018	0.107956
CpG_116	82,661,812	LDL	-0.001579	0.00906	-0.019336	0.016178	0.861623
CpG_116	82,661,812	Triglycerides	-0.008632	0.008828	-0.025933	0.00867	0.32816

[a] Numbering and the precise localization of CpG sites/units within *CDH13* promoter region is provided in Figure S2. CpG sites 1-15 are located upstream from CpG island, sites 16-116 are within the CpG island and sites 18-26 are in the first exon.

[b] Genomic position on chromosome 16 is according to GRCh37/hg19.

[c] Effects (methylation on scale from 0-100% effect on cardio-metabolic traits) and *P* -values are calculated using linear regression, including age, gender and experiment series as covariates to the model.

[d] Results were combined using the inverse-variance method under fixed-effects model. Estimated Bonferroni threshold α=0.05/ [5 (independent parameters) x 46 (CpG sites/units) x 2 (HYPEST and CADCZ studies)]=1.09×10^{-4}.

SE, standard error; CI, confidence interval.

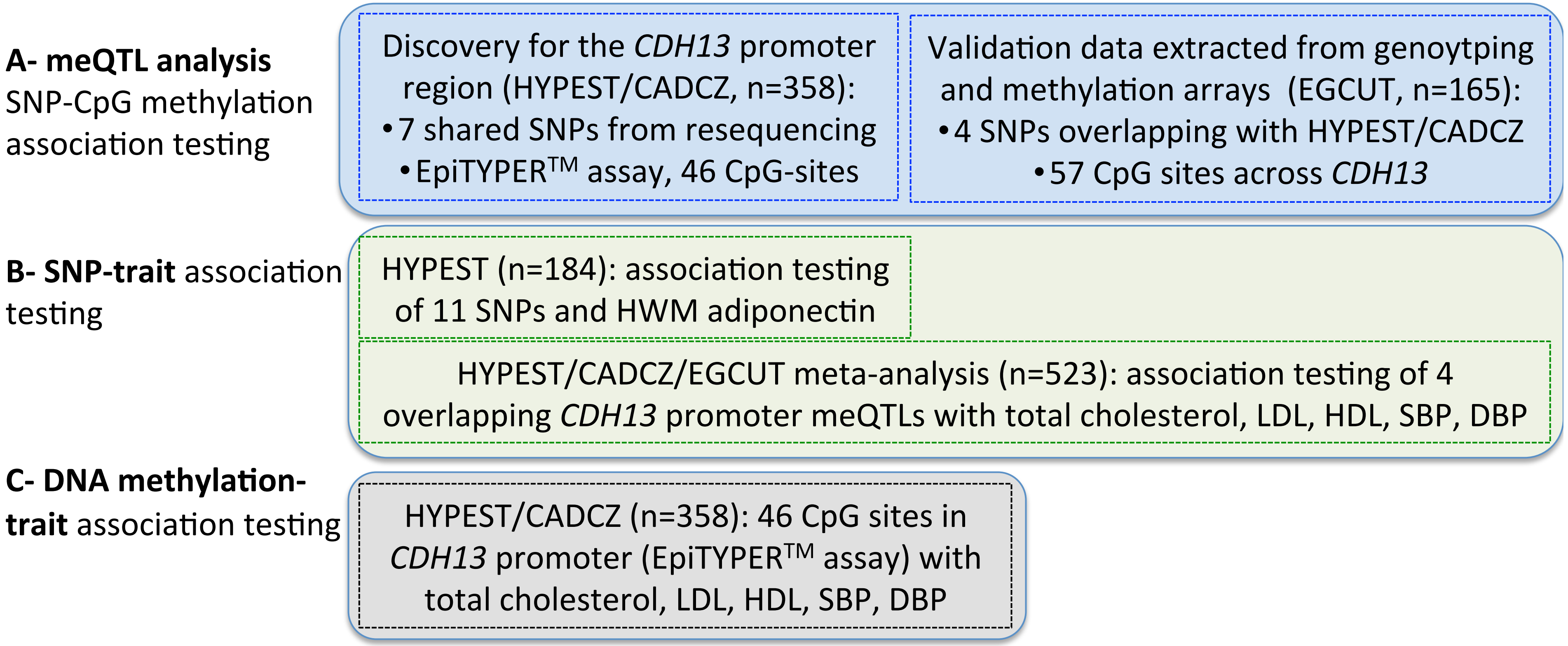
A- meQTL analysis
SNP-CpG methylation association testing
Discovery for the CDH13 promoter region (HYPEST/CADCZ, n=358):
•7 shared SNPs from resequencing
•EpiTYPERTM assay, 46 CpG-sites
Validation data extracted from genoytping and methylation arrays (EGCUT, n=165):
•4 SNPs overlapping with HYPEST/CADCZ
•57 CpG sites across CDH13
B- SNP-trait association testing
HYPEST (n=184): association testing of 11 SNPs and HWM adiponectin
HYPEST/CADCZ/EGCUT meta-analysis (n=523): association testing of 4 overlapping CDH13 promoter meQTLs with total cholesterol, LDL, HDL, SBP, DBP
C- DNA methylation-trait association testing
HYPEST/CADCZ (n=358): 46 CpG sites in CDH13 promoter (EpiTYPERTM assay) with total cholesterol, LDL, HDL, SBP, DBP

Supplemental Figure S1 Overview of the study data and analysis.

(A) In the discovery analysis, seven *CDH13* promoter SNP shared by HYPEST and CADCZ were tested for association with DNA methylation levels at the 46 studied *cis*-CpG-sites/units. For meQTL confirmation analysis in the EGCUT sample set, the SNPs in the *CDH13* promoter region overlapping with the HYPEST/CADCZ dataset (rs12443878, rs12444338, rs62040565, rs8060301) were assessed for the effect on the DNA methylation level at the CpG-sites measured across the *CDH13* gene (n=57) in EGCUT. **(B)** The effect of genotypes on cardio-metabolic traits (total cholesterol, LDL, HDL, triglycerides, SBP, DBP) was tested in the meta-analysis across HYPEST, CADCZ and ECGUT for the four SNPs (rs12443878, rs12444338, rs62040565, rs8060301) shared by the three datasets. Genetic association testing with serum HMW adiponectin levels was applicable for the HYPEST samples (not available for CADCZ and EGCUT) for 11 SNP detected in resequencing. **(C)** The effect of methylation levels at the 46 *CDH13* promoter CpG-sites/units on serum lipids and BP in HYPEST and CADCZ subjects was assessed by meta-analysis across the two samples. No validation was sought as the discovery analysis did not reach significant results.

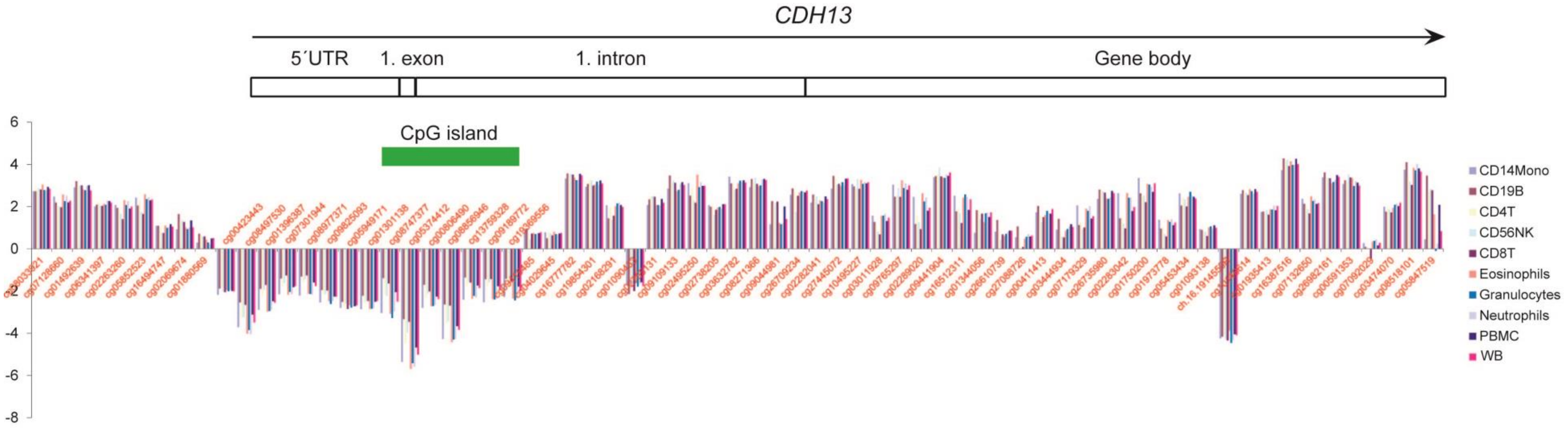

Supplemental Figure S2 *CDH13* DNA methylation in different blood cell types. DNA methylation of 69 CpG-sites (red codes) covering *CDH13* locus on Infinium HumanMethylation450 BeadChip is defined as M-value (described in Reinius et al., 2012). Vertical bars (10 per each CpG-site) represent nine blood cell types and whole blood (listed on the right). Positive values indicate to methylated and negative values to unmethylated state. Location of CpG-sites in the *CDH13* gene is shown at the top.

82,660,291 1 2

AGAATCTTTTTGCACTAGGCTGGAAGTGGCCGCCAGTCCCCCGTGCA

3

ATTCCATTCTCTGGAAAAGTGGAATCAGCTGGCATTGCCCAGCGTGA

TTTGTGAGGCTGAGCCCCAACAGTCCAAAGAAGCAAATGGGATGCCA

4 5 6 7 8 9

CCTCCGCGGGGCTCGCTCCTCGCGAGGTGCTCACCCCGTATCTGCCA

10 11 12

TGCAAAACGAGGGAGCGTTAGGAAGGAATCCGTCTTGTAAAGCCATT

13

GGTCCTGGTCATCAGCCTCTACCCAATGCTTTCGTGATGCTGCTGCT

14

GATCTATTTGGGAAGTTGGCTGGCTGGCGAGGCAGAGCCTCTCCTCA

15 1617

AAGCCTGGCTCCCACGGAAAATATGCTCAGTGCAGCCGCGTGCATGA

18 19 20 21 22 23

ATGAAAACGCCGCCGGGCGCTTCTAGTCGGACAAAATGCAGCCGAGA

24 25 26

ACTCCGCTCGTTCTGTGCGTTCTCCTGTCCCAGGTAGGGAAGAGGGG

27 2829 30 31 32 33 34

CTGCCGGGCGCGCTCTGCGCCCCGTTTCTGCATTCGGATCGCCCGGC

35 36 37 38 3940

ACGGGCAGGGTGAGGGGGCTTTCGGGGGGTCGGGGCCTCCGGTCGCG

41 42 43 44 45

GCGGCGAAGACAGATCGGGGCTCGGTAGGGAGGTCATTCCGAGCCCA

46 47

GAGATCCTAGGCACCCCCCACACACAGGCTCCCACTCTGGCGTGCGT

48

GTGTGTGTGTGTGTGTGTGTGTGTGTGTGTGTGTGTGTGTGTACGTT

49 50

CGTTAACGGGAGGAGGAGAGAGCTCCCAGTCCTTTTTTGCTAGCAGG

51 52

GGCGACATTCTCGCCCACATCAAGTGGGGTAACTTTGGTTCCCTCCT

53 54 55
CCGGAGGCTCGGTGCATTGGAGAAAGACTCAGTTAGAGGCGACTCCA
56 5758 59 60 61 62
ACGAGCCGCGGTTTTCCCCAGCCCAACGCCCAGCGGCCGAAGCGCTG
63 64 65 66 67
CTCGGGTCCGGATTGCGGGATGCGGGGCTGGAGAGGCCGAGCAGGCA
68 69 70 71
CCACCGACTTCCCAGGGCGCCCGGGCCCCCTGGTACAGCCCGGCTGC
72 73 74 75
CCGCTGGAAGGCGCCTCGGGGCAGCAGAGAGCCTCAGCCCGGCTGCT
76 77 78 79 80 81 82 83
GCTGTCGCTCAAAGGCGCCGGCGCCGGCCGCACCCGCATCGGGGTCC
84 85 86 87 88
TTTTGCTCCCAGACCCCGGGCCCGAAAGGGCCGGAGCGTGTCCCCCG
89 90
CCAGGGCGCAGGCCCCAGCCCCCCGCACCCCTATTGTCCAGCCAGCT
91 92 93
GGAGCTCCGGCCAGATCCCGGGCTGCCGCCTCTGCTGCCTTCCCTGA
94 95 96 97
GCGGGAGCGGAGCGCAGAGAAAAGTTCAAGCCTTGCCCACCCGGGCT
98 99 100101
GCAGCTGCTTGTTAACCCTCAGAGCGCCACGGCGCGAGGGAAGGGCA
102 103 104 105
CGCCAACCAGGAGAGGGGGCGAGGGAGATGCGGTCCGCCTGCAGTCA
106 107
CCTCTGCACCTCAGAGATTTCGGGAAGTTTGAGTGCAGGAAAGCAGC
108 109 110 111
GCTCCGAGGCCAGGCCTGGGGTGCTGGCCGCTGCGGGGGGCACGCCC
112 113 114
TGCGCTGCTCAGGGGCCTGTGGTTTCGGAGAGCACCCCGATCCAGTC
115 116
CCCCATCGCCTCTCTGGCAGGCGTTGGACTTGGAGTGAGCTGGCAG
82,661,877
CCTGCAAGTGGGTGGATAAGAGCCAGGGCAGGGCAGGGCCGGGCAGA

Supplemental Figure S3 *CDH13* promoter region targeted for DNA methylation analysis. Arrows indicate the start and end positions of the region on chromosome 16 targeted for methylation measurement. CpG sites marked bold and red are sites in the targeted region. Numbers coloured red indicate sites that passed QC and were used in the association analysis. The CpG island covers sites 16-116. The sequence of the first exon is underlined covering CpG sites 18-26. Assay 1-yellow background, assay 2- grey background, assay 3-light blue background, assay 4- purple background, overlapping assays- dark blue (**Table S2**).

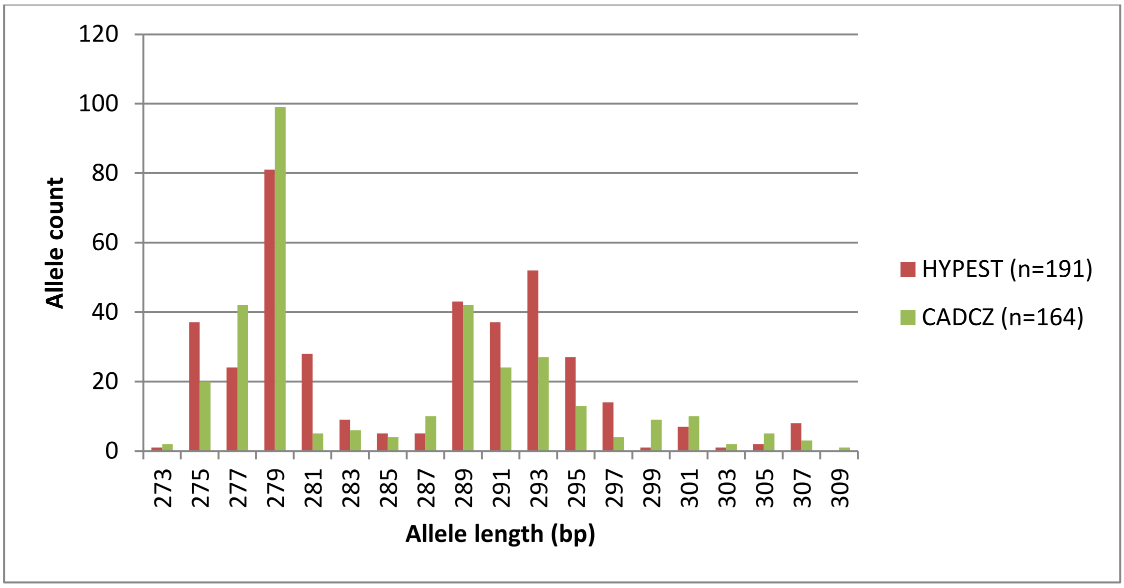

Supplemental Figure S4 Distribution of microsatellite length variants in HYPEST and CADCZ. On the x-axis the length (bp) of alleles identified by fragment analysis on 3730xl DNA Analyzer is shown. The y-axis shows the number of alleles identified in HYPEST (n=191) and CADCZ (n=164) study groups.

References to Supplemental Materials and Methods

1000 Genomes Project Consortium, Abecasis GR, Auton A, Brooks LD, DePristo MA, Durbin RM, Handsaker RE, Kang HM, Marth GT, McVean GA. An integrated map of genetic variation from 1,092 human genomes. *Nature*. 2012;491(7422):56-65.

Adalsteinsson BT, Gudnason H, Aspelund T, Harris TB, Launer LJ, Eiriksdottir G, Smith AV, Gudnason V. Heterogeneity in white blood cells has potential to confound DNA methylation measurements. *PLoS One*. 2012;7(10):e46705

Aryee MJ, Jaffe AE, Corrada-Bravo H, Ladd-Acosta C, Feinberg AP, Hansen KD, Irizarry RA: Minfi: a flexible and comprehensive Bioconductor package for the analysis of Infinium DNA methylation microarrays. *Bioinformatics* 2014

Bell JT, Tsai PC, Yang TP, Pidsley R, Nisbet J, Glass D, Mangino M, Zhai G, Zhang F, Valdes A, et al. Epigenome-wide scans identify differentially methylated regions for age and age-related phenotypes in a healthy ageing population. *PLoS Genet*. 2012;8(4):e1002629.

Ehrich M, Nelson MR, Stanssens P, Zabeau M, Liloglou T, Xinarianos G, Cantor CR, Field JK, van den Boom D. Quantitative high-throughput analysis of DNA methylation patterns by base-specific cleavage and mass spectrometry. *Proc Natl Acad Sci USA*. 2005;102(44):15785-90

Gomes MV, Toffoli LV, Arruda DW, Soldera LM, Pelosi GG, Neves-Souza RD, Freitas ER, Castro DT, Marquez AS. Age-related changes in the global DNA methylation profile of leukocytes are linked to nutrition but are not associated with the MTHFR C677T genotype or to functional capacities. *PLoS One*. 2012;7(12):e52570.

Howie BN, Donnelly P, Marchini J. A flexible and accurate genotype imputation method for the next generation of genome-wide association studies. *PLoS Genet*. 2009;5(6):e1000529.

Janosíková B, Pavlíková M, Kocmanová D, Vítová A, Veselá K, Krupková L, Kahleová R, Krijt J, Kraml P, Hyánek J, et al. Genetic variants of homocysteine metabolizing enzymes and the risk of coronary artery disease. *Mol Genet Metab*. 2003;79(3):167-75.

Leitsalu L, Haller T, Esko T, Tammesoo ML, Alavere H, Snieder H, Perola M, Ng PC, Mägi R, Milani L, et al. Cohort Profile: Estonian Biobank of the Estonian Genome Center, University of Tartu. *Int J Epidemiol*. 2014;[Epub ahead of print]

Maksimovic J, Gordon L, Oshlack A. SWAN: Subset-quantile within array normalization for illumina infinium HumanMethylation450 BeadChips. *Genome Biol*. 2012;13(6):R44.

Org E, Veldre G, Viigimaa M, Juhanson P, Putku M, Rosenberg M, Tomberg K, Uuetoa T, Laan M. HYPEST study: profile of hypertensive patients in Estonia. *BMC Cardiovasc Disord*. 2011;11:55.

Reinius LE, Acevedo N, Joerink M, Pershagen G, Dahlén SE, Greco D, Söderhäll C, Scheynius A, Kere J. Differential DNA methylation in purified human blood cells: implications for cell lineage and studies on disease susceptibility. *PLoS One*. 2012;7(7):e41361.

Tobin MD, Sheehan NA, Scurrah KJ, Burton PR. Adjusting for treatment effects in studies of quantitative traits: antihypertensive therapy and systolic blood pressure. *Stat Med*. 2005;24(19):2911-35.

Westra HJ, Jansen RC, Fehrmann RS, te Meerman GJ, van Heel D, Wijmenga C, Franke L. MixupMapper: correcting sample mix-ups in genome-wide datasets increases power to detect small genetic effects. *Bioinformatics*. 2011;27(15):2104-11.

Wu J, Province MA, Coon H, Hunt SC, Eckfeldt JH, Arnett DK, Heiss G, Lewis CE, Ellison RC, Rao DC, et al. An investigation of the effects of lipid-lowering medications: genome-wide linkage analysis of lipids in the HyperGEN study. *BMC Genet*. 2007;8:60.
